# Targeting KRAS^G13C^ with cyclic linker based inhibitors to explore warhead orientation

**DOI:** 10.1038/s41598-025-22145-5

**Published:** 2025-10-31

**Authors:** Tonia Kirschner, João Rodriguez, Emerson Gonçalves Moreira, Janina Niggenaber, Jonas D. Warmuth, Hugo Verli, Matthias P. Müller, Daniel Rauh

**Affiliations:** 1 Department of Chemistry and Chemical Biology, Drug Discovery Hub Dortmund (DDHD), TU Dortmund University, Zentrum für Integrierte Wirkstoffforschung (ZIW), Otto-Hahn-Strasse 4a, 44227 Dortmund, Germany; 2 Programa de Pos-Graduacao em Biologia Celular e Molecular (PPGBCM), Centro de Biotecnologia, Universidade Federal do Rio Grande do Sul (UFRGS), Av. Bento Goncalves, Porto Alegre, 9500, CEP 91501-970 RS Brazil

**Keywords:** KRAS, G13C, Nucleotide-based inhibitors, Computer-aided drug design, Cancer, Chemical biology, Computational biology and bioinformatics, Structural biology

## Abstract

The small GTPase KRAS is a key driver of carcinogenesis when mutated, and significant progress has been made in targeting KRAS^G12C^ and other oncogenic variants. Building on our previous work demonstrating the potential of nucleotide-based inhibitors with an acrylamide warhead to target KRAS^G13C^, we designed and synthesized a library of nucleotide-based compounds with cyclic linkers to explore the effect of warhead orientation on reactivity toward Cys13. Using mass spectrometry, kinetic studies, and protein X-ray crystallography, we validated the binding and reactivity of these modulators. In addition, computational predictions of the conformational space of the linkers and warheads provided insights into their reactivity, which agreed well with the experimental data. These findings advance our understanding of the structure-reactivity relationship in these nucleotide-based KRAS inhibitors and will be the basis for further optimization.

**Supplementary Information:**

The online version contains supplementary material available at 10.1038/s41598-025-22145-5.

## Introduction

**Fig. 1:**
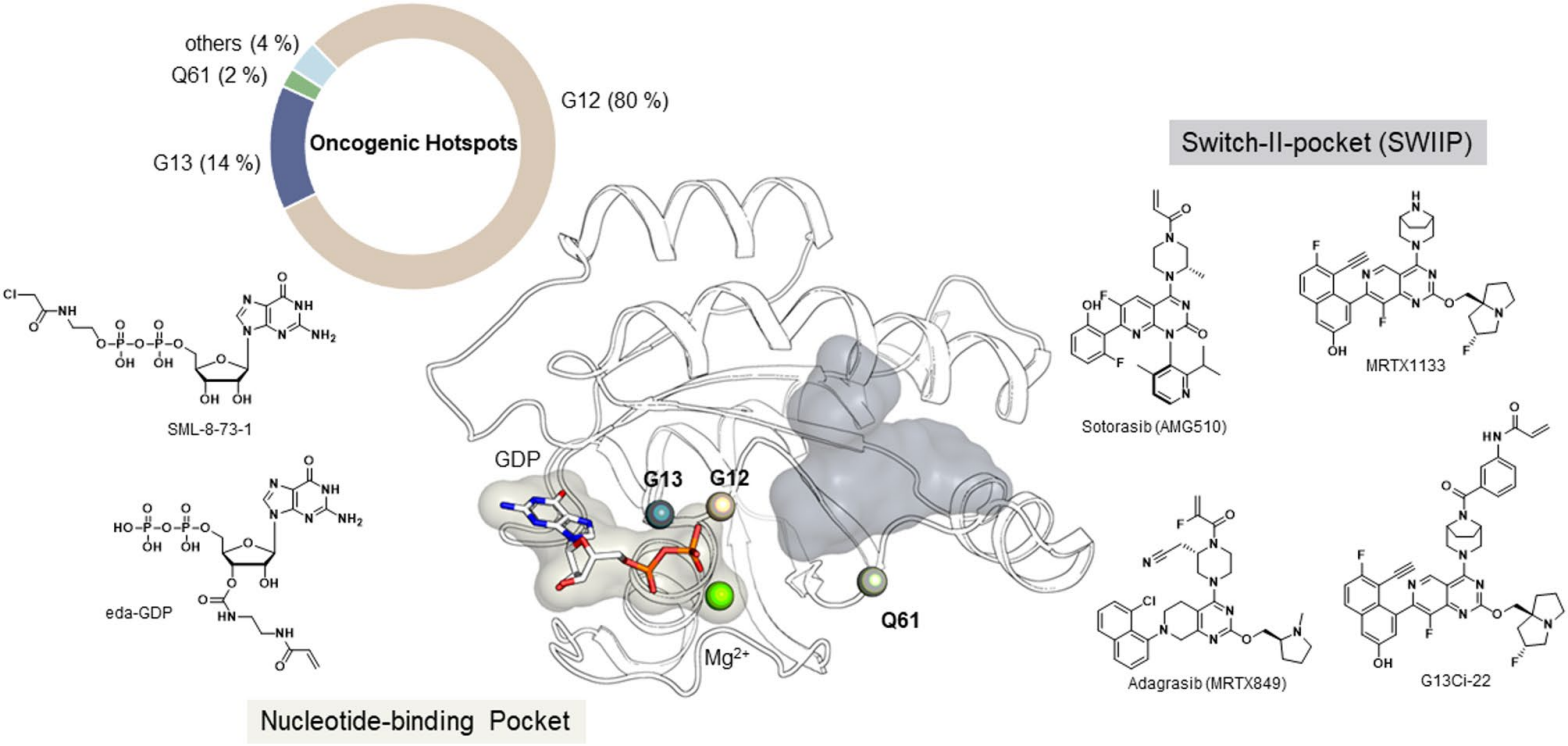
Overview of important binding pockets and oncogenic mutations in KRAS. Mutational frequencies of mutations occurring in KRAS (retrieved from the COSMIC database; 5th February 2025^37^). The nucleotide-binding pocket (beige) is shown on the left and the SWIIP is shown on the right (grey), including the position of the oncogenic mutations at positions G12, G13 and Q61 (grey, beige and green spheres, respectively; structure based on PDB ID 7RPZ), including exemplified molecules binding to the pockets as mentioned above. PyMOL (version 3.1.0, W.L. DeLano, The PyMOL Molecular Graphics System) was used for generating the 3D figures.

 Kirsten rat sarcoma (KRAS) is one of the most prominent oncogenes in human tumors, found in approximately 30 % of cases^1^. Oncogenic mutations in KRAS cause a shift of equilibrium from the inactive guanosine diphosphate (GDP)-bound state to the active guanosine triphosphate (GTP)-bound state, thereby amplifying proliferative signaling pathways and promoting uncontrolled cell growth^2–4^. Historically considered “undruggable,” targeting KRAS experienced a breakthrough led by Kevan Shokat’s research group^5^. Their discovery of an allosteric binding site, known as the switch II pocket (SWIIP; Fig. 1), enabled the development of small-molecule inhibitors that modulate this small GTPase^6^. This breakthrough led to the development of covalently binding molecules such as sotorasib (AMG510, LUMYKRAS^®^, Amgen)^7^ and adagrasib (MRTX849, KRAZATI^®^, Bristol Myers Squibb)^8,9^, which received FDA approval in 2021 and 2022, respectively. This revolutionized the treatment of KRAS^G12C^-mutated non-small cell lung cancer (NSCLC), a condition previously lacking effective targeted therapy.

Initially, acrylamide-based inhibitors targeting the acquired cysteine residue specifically addressed G12C mutations. However, recent years have seen the emergence of novel strategies utilizing SWIIP binders incorporating mutant-specific moieties for G12D^10^,–^12^G12S^13^, and G12R mutations^14^. These advancements promise innovative approaches to address cancer types harboring mutations beyond G12C^15^. Another significant mutation hotspot within the KRAS gene is codon 13, where mutations occur in approximately 14 % of tumors^16^. Within this subset, 6 % feature a glycine-to-cysteine substitution (KRAS^G13C^), presenting a potential anchor point for the design of covalent inhibitors^17,18^. Currently, the first KRAS^G13C^ inhibitor, RMC-8839, is in preclinical development utilizing Tri-complex (“molecular glue-like” compounds) inhibitors, initially reported by Zhang et al.. and subsequently advanced by Revolution Medicines^19,20^. Additional pockets and grooves on the surface of RAS have also been described^21,22^ (e.g. the switch I/II pocket, Y32, D38, A59 site) and potent small molecules have been developed that target these (e.g. BI-2852^23,^^24^, KRB-456^25^, compound 3144^26^), but have not advanced to clinical application. In addition to the possibility of allosteric inhibition, there is also the option of occupying the guanine nucleotide-binding pocket with an inhibitor that locks the GTPase in an inactive state. Small GTPases, such as KRAS, have an extremely high affinity for GDP and GTP^27,28^. Unlike kinases, which have a more moderate affinity for adenosine triphosphate (ATP) and where ATP-competitive inhibitors have been successfully developed^29^, orthosteric inhibition of KRAS initially appeared challenging^30^. However, Lim et al. and Hunter et al. first demonstrated a nucleotide-based KRAS^G12C^ inhibitor (SML 8–73−1) that competes for the nucleotide-binding pocket (Fig. 1) and irreversibly reacts with the acquired Cys12, thereby competing with the natural substrates. These modulators, however, exhibited significantly reduced protein affinity due to modifications of the *β*-phosphate group. Recently, the Shokat lab reported the successful targeting of KRAS^G13C^ using the scaffold of the KRAS^G12D^ inhibitor MRTX1133, using an extended linker to reach Cys13 (G13Ci-22, Fig. 1)^33^.

Our previous work introduced nucleotide-based KRAS^G13C^ inhibitors featuring a linker and acrylamide moiety, specifically targeting this mutation^34^. Compared to the compound set by Lim et al.^31^, our modifications at the 2’- or 3’-position of the ribose with the linker and the warhead preserved the high reversible affinity of the nucleotide. In this study, we utilized the acrylamide warhead, renowned for its biocompatibility due to its low non-specific reactivity towards thiol groups, which necessitates an optimal orientation of the acrylamide warhead towards the target cysteines after the initial association step and in the reversibly bound state^3,35^.

However, our previously developed molecules displayed comparatively low reactivity towards the targeted Cys13 in KRAS, potentially due to an unfavorable preorientation of the aliphatic linear linker. In this study, we report the synthesis, reactivity, and structural profiling of novel compounds featuring cyclic aliphatic linkers. Additionally, we employed *in silico* methods to predict the conformational space of the linker and warhead in the reversibly bound state for nucleotide-based compounds to explore the molecular basis for the compounds’ reactivities and to guide future linker design.

## Results and discussion

### Chemistry and biological experiments

#### Design and synthesis of acryl-bearing GDP-based inhibitors

**Fig. 2:**
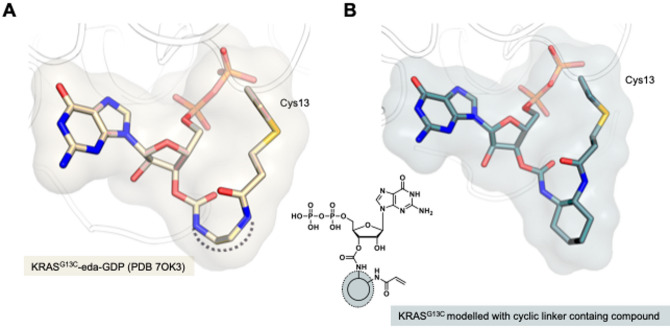
Design of novel nucleotide-based inhibitors with cyclic linkers (**A**): Co-crystal structure of KRAS^G13C^ covalently modified by eda-GDP (PDB 7OK3) indicating a bend conformation of the linker regions. (**B)**: Modelled pose of a GDP-based inhibitor featuring a cyclic linker in KRAS^G13C^ (modelling carried out in LigandScout^37^, based on PDB ID 7OK3). PyMOL (version 3.1.0, W.L. DeLano, The PyMOL Molecular Graphics System) was used for generating the 3D figures.

We started with a detailed structural analysis based on the previously published co-crystal structures of KRAS^G13C^ in complex with eda-GDP and bda-GDP (PDB IDs 7OK3 and 7OK4, respectively). Closer examination revealed that the linker regions of both molecules adopt a distinctly bent conformation to facilitate covalent interaction with Cys13 (illustrated for eda-GDP (7OK3) in Fig. 2). This flexibility is attributed to the aliphatic linear nature and, thus, increased adaptability of the linkers. Based on these findings, we aimed to rigidify the linker by replacing the linear design with cyclic linkers. This modification is expected to promote improved pre-orientation of the warhead, thereby increasing covalent binding efficiency.

**Fig. 3:**
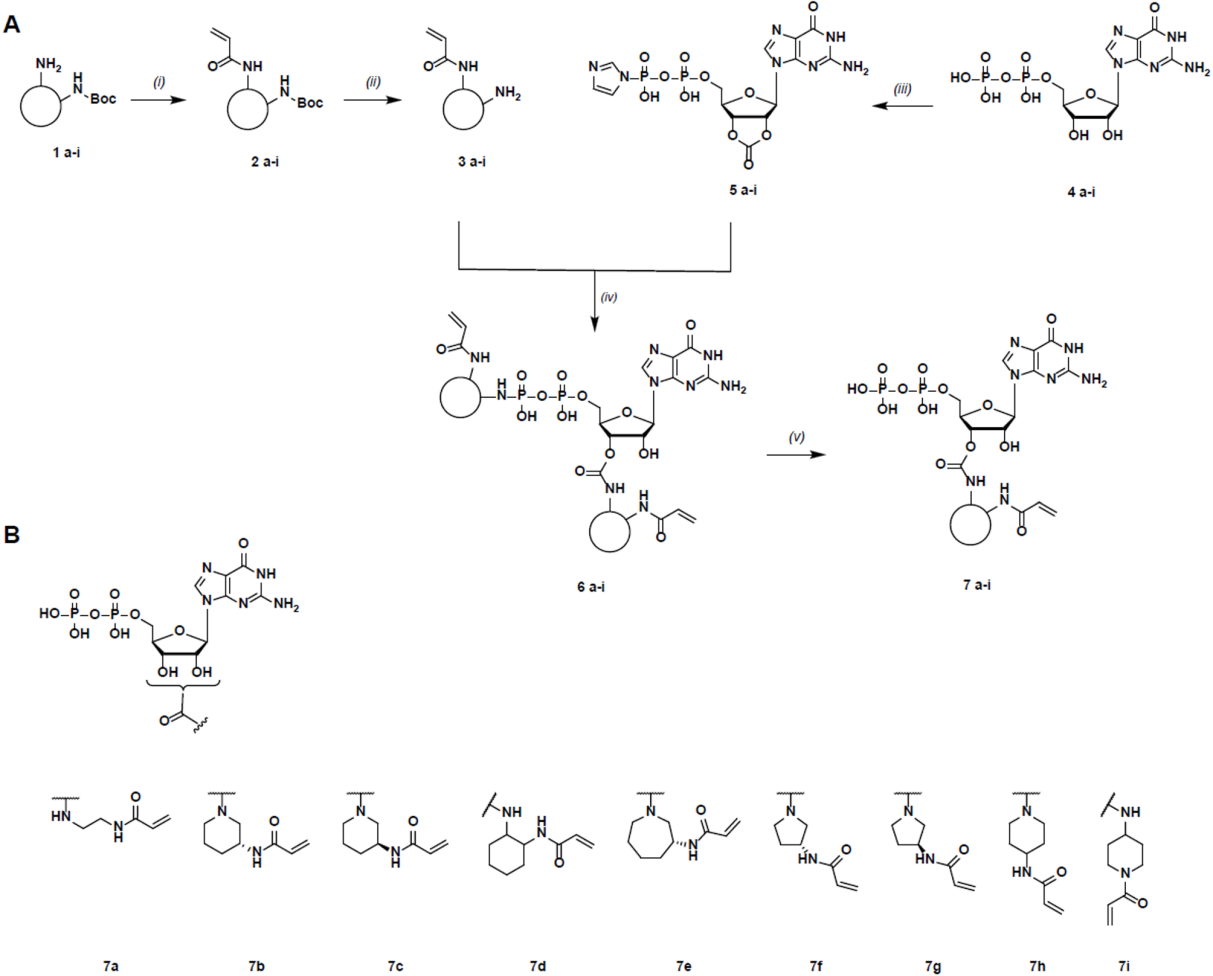
Convergent synthesis scheme of novel nucleotide-based inhibitors.^a^ (**A**):^a^ Reagents and conditions: (*i*) Acryloyl chloride, DIPEA, THF, rt, 12 h; (*ii*) DCM/TFA (3:1), rt. (*iii*) CDI, DMF, 4 °C, 12 h; (*iv*) DIPEA, DMF, rt, 12 h; (*v*) aq. HCl (pH 1.5) 1**–**7. (**B)**: Acrylamide-bearing GDP-based inhibitors **7a**-**7i**.

We synthesized a focused library of eight compounds featuring cyclic linkers (Fig. 3). To achieve this, we introduced a new synthetic strategy compared to the previously established protocol by Goebel et al.^35^, where the GDP nucleotide was protected with carbonyldiimidazole (CDI). Concurrently, the linker, a cyclic diamine, was modified with acryloyl chloride, and these two units were subsequently assembled, and the phosphate-moiety further deprotected to form the desired nucleotide-based inhibitors as 2’ and 3’-isomers (Fig. 3A, the synthesis and analysis of intermediates are described in detail in the methods section and Tables 1 and 2).

#### Selective covalent modification of KRAS^G13C^

**Fig. 4:**
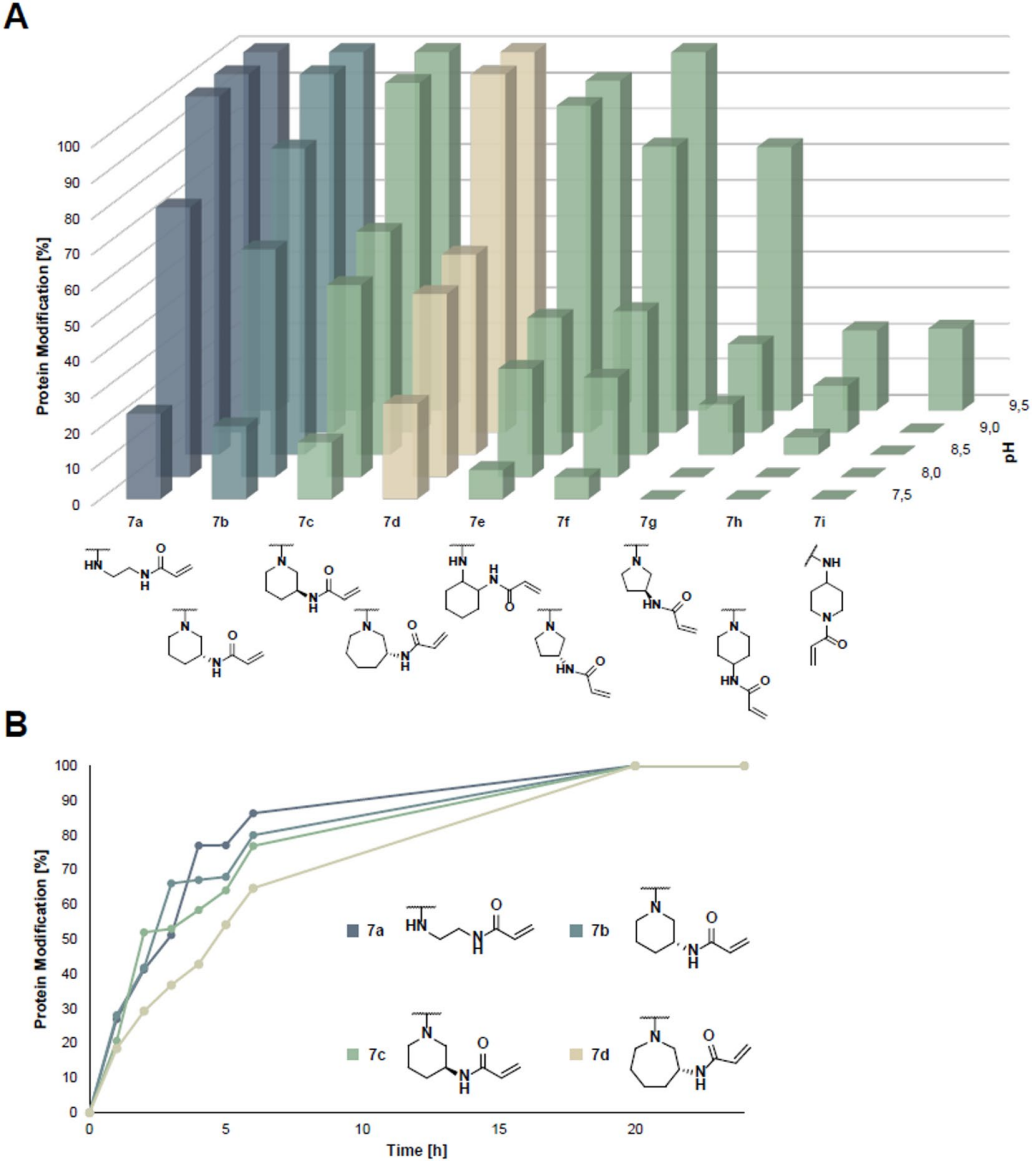
Novel nucleotide-based inhibitors modify KRAS^G13C^ covalently. (**A**): pH-dependent covalent modification of KRAS^G13C^_1−169_ (Cys-light) proteins at pH 7.5–9.5 after 24 h at rt (the graph was prepared in Excel). (**B)**: Time-dependent analysis of the covalent modification of KRAS^G13C^_1−169_ (Cys-light) at pH 9.5 and rt.

In mass spectrometry (MS) studies with KRAS^G13C^_1−169_ (Cys-light), we observed covalent modification of the desired cysteine 13 for all compounds and that the reactivity increased with rising pH levels, which can be attributed to the enhanced nucleophilicity of the cysteine. The starting molecule, acrylic-eda-GDP **7a**, exhibited a reactivity profile similar to molecule **7b**. Furthermore, linkers modified at the 1,3-positions on 6-membered rings (**7b** and **7c**) showed the highest reactivity, followed by those on 5- and 7-membered rings (**7d** and **7f**). This observation suggests that the positioning of the warhead in the 1,3 configuration promotes a preferred orientation, thereby facilitating the covalent reaction. Conversely, 1,4-modified 6-membered rings (**7h** and **7i**) were the least effective, likely due to their high rigidity and linear nature. Additionally, the *R* configuration at the warhead-carrying position was slightly favored (Fig. 4A). However, it is important to note that all molecules reacted rapidly at elevated pH, suggesting potential nonspecific interactions between the deprotonated Cys13 and the warhead of the nucleotides, irrespective of their binding to the nucleotide-binding pocket. In addition to KRAS^G13C^, we also investigated the reactivities towards KRAS^G12C^_1−169_ (Cys-light) and KRAS^wt^_1−169_, where no to very low modification was observed, indicating good overall selectivity towards Cys13 (Figure S1). Time-resolved studies of compounds **7b**, **7c**, and **7d** demonstrated modification rates comparable to the parental molecule **7a** (Fig. 4B), with compound **7b** exhibiting slightly superior performance compared to **7c** and **7d**.

#### Inhibitory effects of novel covalent nucleotide-based inhibitors

**Fig. 5:**
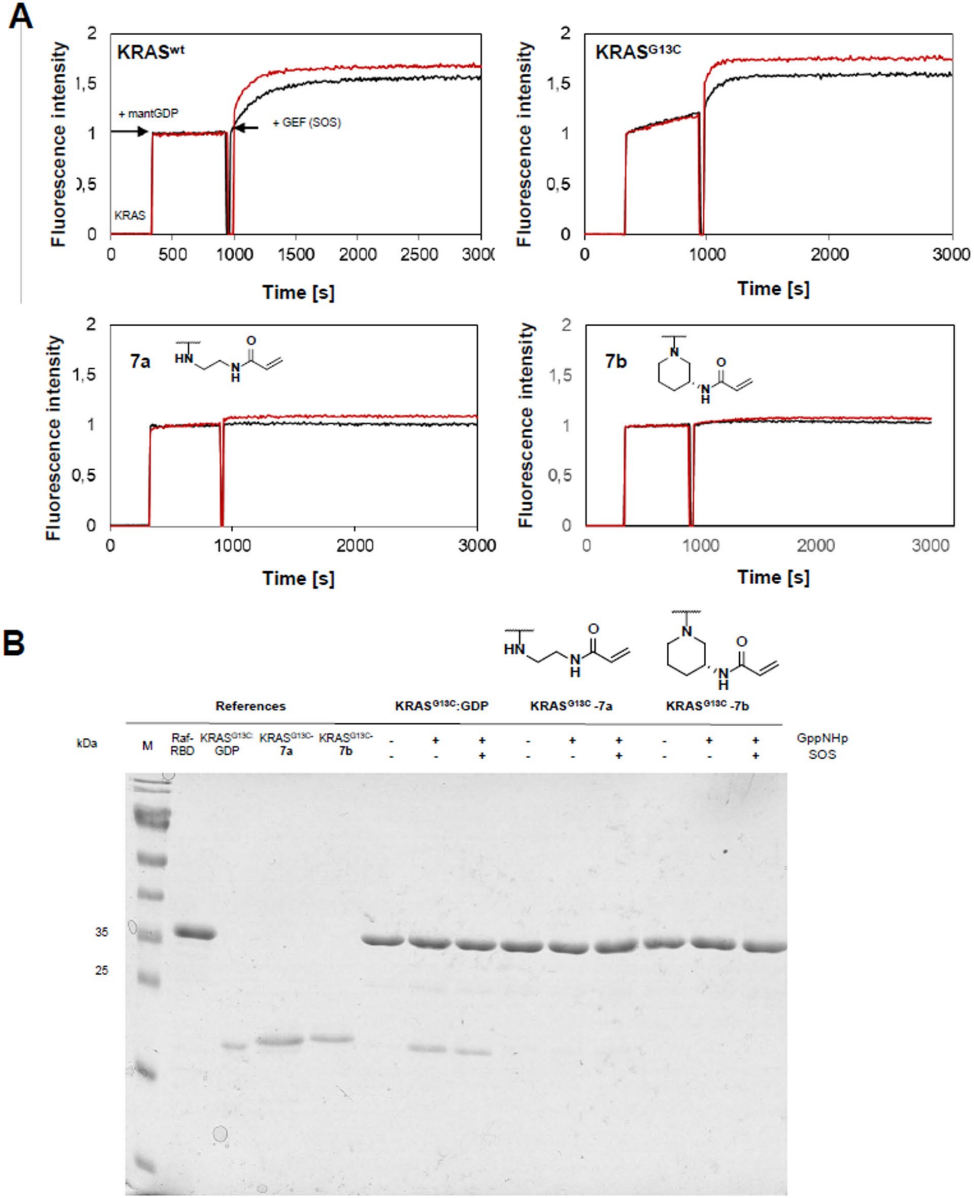
The nucleotide-analogues lock RAS in an inactive state. (**A**): GEF-catalyzed nucleotide exchange of KRAS^WT^:GDP, KRAS^G13C^:GDP and KRAS^G13C^-**7a** and **7b**. In the assay, 5 µM of the preparatively modified or unmodified KRAS (residues 1–169) was incubated with 10 µM mant-dGDP, followed by the addition of SOS at varying concentrations (0.25 µM and 0.5 µM; black and red curves, respectively). (**B)**: Pull-down-Assay. Whereas KRAS^G13C^:GDP can be pulled down by GST-tagged Raf RBD in the presence of GppNHp and GppNHp/SOS, KRAS^G13C^-**7a/7b**:GDP cannot become activated and consequently cannot bind to the Raf RBD, neither in the presence of GppNHp only nor GppNHp and SOS. The unprocessed SDS gel is shown in Figure S2.

It was also interesting to assess the synthesized compounds’ inhibitory activity. For this purpose, we utilized a son of sevenless (SOS)-catalyzed nucleotide exchange assay. Consistent with previous reports, we observed an increased intrinsic exchange rate in the KRAS^G13C^ mutant compared to wild-type KRAS, presumably contributing to its oncogenic nature^34^. Upon modification of the protein with the covalent nucleotide analogs, no nucleotide exchange was observed for compounds **7a** and **7b**, including the linear and cyclic linkers, respectively, that showed the highest reactivities in the previous experiments. This indicates that the protein is effectively blocked in its inactive state and can no longer be activated (Fig. 5A). In a second assay, we investigated whether SOS-catalyzed nucleotide exchange to a non-hydrolyzable GTP analog (guanosine-5’-[(*β*,*γ*)-imido]triphosphate, GppNHp) and effector binding were still possible after covalent modification with the ligand. A pull-down experiment was conducted using a glutathione S-transferase (GST)-tagged rapidly accelerated fibrosarcoma (Raf) RAS-binding domain (Raf RBD) to assess this. Adding GppNHp (with and without SOS) enabled the pull-down of unmodified KRAS^G13C^, demonstrating the activation of the GTPase and subsequent effector binding. In contrast, KRAS^G13C^ covalently modified with molecules **7a** and **7b** cannot become activated in the presence of GppNHp and SOS and consequently did not interact with the Raf RBD (Fig. 5B).

#### Co-crystal structure of 7b in complex with KRAS^G13C^ mutant

**Fig. 6:**
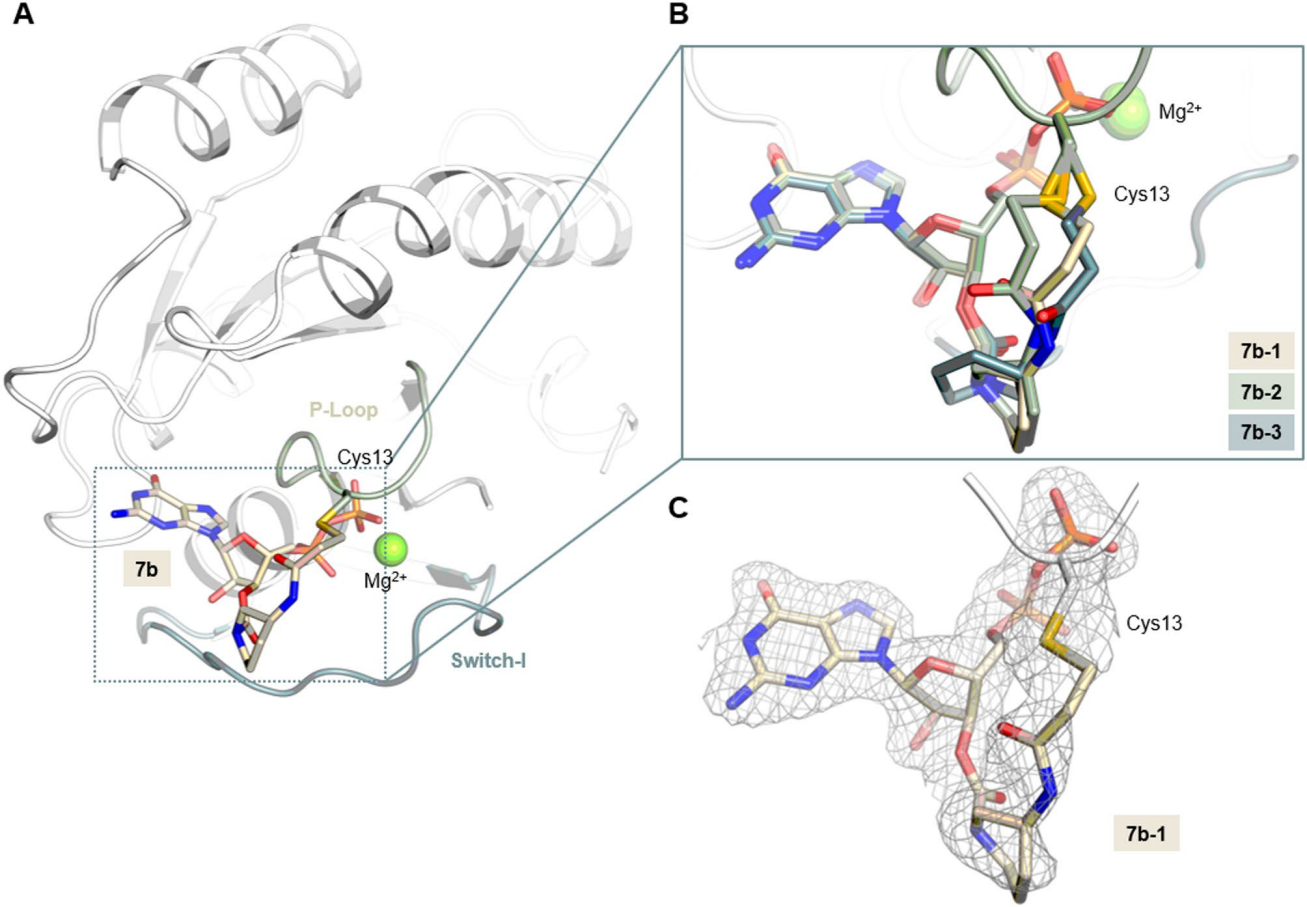
X-Ray crystallographic model of KRAS^G13C^ covalently modified with **7b**. (**A)**: Compound **7b** covalently bound to Cys13 of KRAS^G13C^. (**B)**: 3 copies of covalently modified RAS were observed in the asymmetric unit. The figure indicates that the GDP-core adopts a similar orientation in all of these, whereas the linker is flexible and adopts different conformations and is solvent exposed (also see Figure S3, A). (**C)**: The 2Fo-Fc electron density map is contoured at an r.m.s.d. of 1. PyMOL (version 3.1.0, W.L. DeLano, The PyMOL Molecular Graphics System) was used for generating the 3D figures.

To further characterize the novel nucleotides with the cyclic linkers, KRAS^G13C^ was co-crystallized with compound **7b** to obtain detailed structural insights into ligand binding within the binding pocket (resolution:1.85 Å, R_work_: 18.98, R_free_: 22.78; Fig. 6A; data statistics are shown in Table 3).

Overall, the obtained structure is highly similar to previously published GDP-bound (inactive) wild-type structures (for example PDB ID 4OBE; comparison in Figure S3, B)^32^, highlighting that our design approach preserved the binding geometry of the nucleotide scaffold, which remains similarly oriented and engages in the same reversible interactions important for the high affinity of the nucleotide towards RAS. In contrast to the GDP core, the linker is solvent exposed and not involved in additional interactions with the protein. The molecule is generally also similarly oriented compared to the previously published compound **7a** (PDB 7OK3; Figure S3, C). However, whereas a small shift of the 2’- and 3’-OH groups of the ribose by approximately 1 Å can be observed for compound **7a** that is possibly induced by the highly strained and bend conformation of the linker, this is not the case for compound **7b** (Figure S3, A and B). Interestingly, three molecules of RAS were found in the asymmetric unit, with the orientation of the linker near the covalently modified Cys13 modeled differently in these 3 instances (Fig. 6B), and the electron density less well-defined than in the remaining molecule (Figure S3). This suggests flexibility of the linker, which is consistent with the results of MS studies and indicates that the linker is less strained compared to compound **7a**. However, it also suggests that pre-organization of the linker still needs to be optimized by further rigidification to contribute to an efficient covalent reaction.

## Computational experiments

To gain a better understanding of the chemical nature of the linkers and their associated reactivities, computational predictions of the conformational space of the linkers and the warhead within the nucleotide-based inhibitors and KRAS^G13C^ were performed. We employed a strategy consisting of: (i) using DockTScore within the DockThor docking engine^38^ to generate reliable reversible complexes between acrylamide-containing GDP-based inhibitors **7a**-**i** and KRAS^G13C^, and (ii) refining of these complexes using microsecond MD simulations to explore the proximity of the warhead to Cys13 under native conditions.

**Fig. 7:**
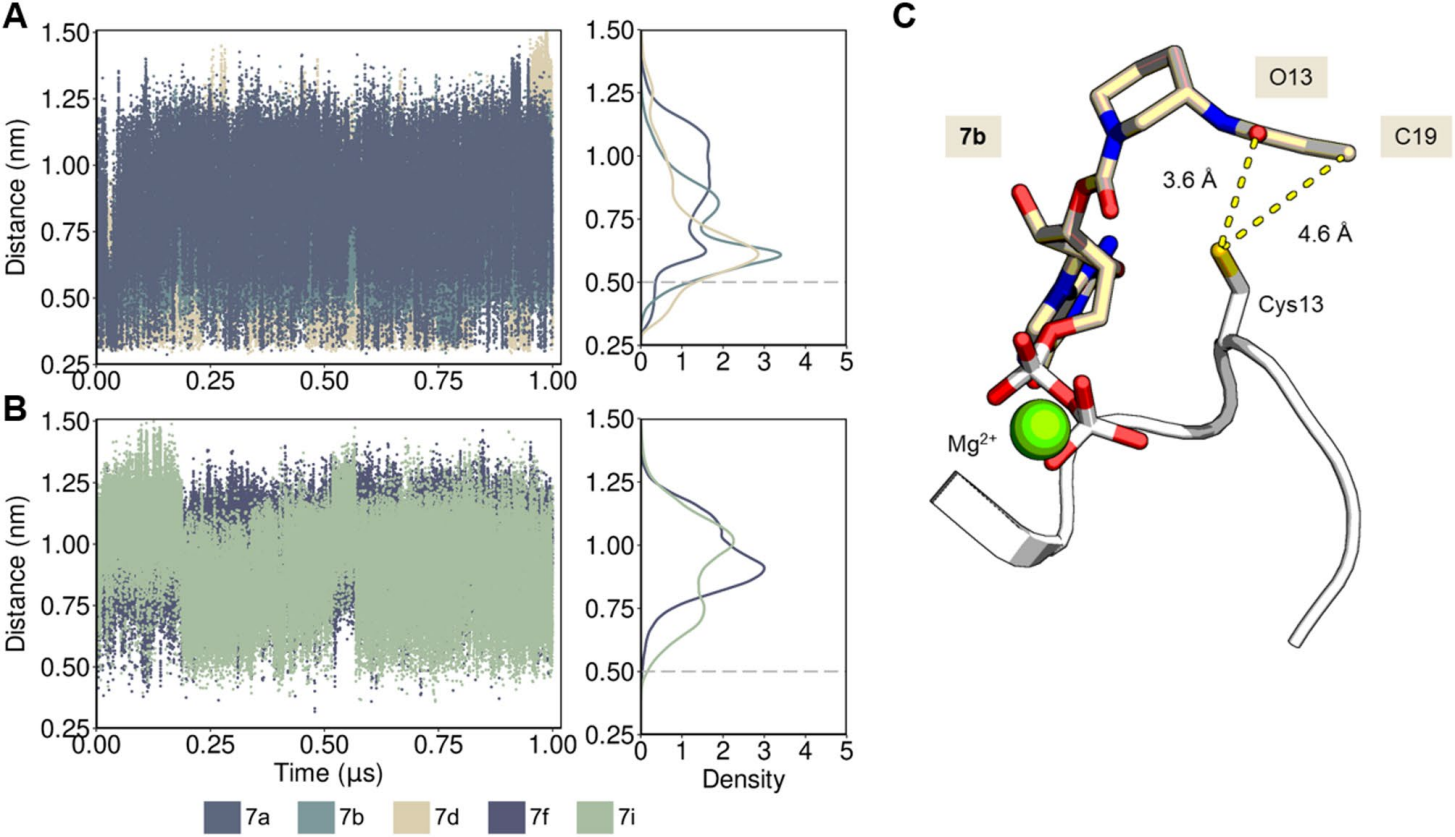
Compound’s solution ensemble when in a reversible complex with KRAS^G13C^, as observed in MD simulations. (**A)** and (**B)**: fluctuation of the distance between Cys13 sulfur atom and the oxygen atom from the α-β-unsaturated carbonyl group is presented for each compound, while the respective distributions of these interatomic distances are indicated in the upper right graph for compounds **7a**, **7b** and **7d**, representing compound with higher activity, and in the lower right graph for compounds **7f** and **7i**, representing compounds with lower activity (see population of conformers below 0.5 nm). The figures show that the less reactive compounds **7f** and **7i** adopt conformations of the warhead close to Cys13 far less often than compounds **7a**, **7b**, and **7c**. C, KRAS^G13C^ in complex with ligand **7d** presenting the warhead in a conformation within 4 Å to the sulfur atom from Cys13. The distance fluctuation (shown in dots) demonstrates that this interatomic distance is in equilibrium during simulation. Also, this ensemble is well reproduced in independent simulations (Figure S8).

The behavior of GDP and Mg^2+^ during simulations with KRAS^G13C^was observed to be highly stable (Figure S4), consistent with the high reversible affinities of nucleotides and Ras described previously in the literature^28,^^30^. Presumably due to the water solvent dynamics and flexibility of switch-I (residues His27-Asp38), we observed a slight shift of Mg^2+^ during the simulations, slightly towards the α-phosphate O3 (Figure S5, Table S1). In contrast, the linker region between GDP and the warhead shows greater structural dynamics (Fig. 7). The reaction of α,β-unsaturated carbonyl covalent inhibitors with KRAS^G13C^ under physiological conditions likely occurs by a concerted mechanism involving proton transfer, nucleophilic addition, and solvent-assisted tautomerization^39^. To understand the differences in reactivity among the studied molecules, we evaluated the interatomic distance between the sulfur atom of Cys13 in KRAS^G13C^ and the oxygen atom of the α,β-unsaturated carbonyl group of each molecule, which characterizes the initial proton transfer step. The synthesized compounds, once bound to KRAS^G13C^, adopt conformations with their warheads mostly far away from Cys13 (distances > 0.5 nm, Fig. 7), while the nucleotide (GDP) remains firmly inserted in the nucleotide-binding pocket (Figure S6). Notably, compounds **7a**, **7b**, and **7d** (Fig. 7, A) adopted warhead conformations near the sulfur atom of Cys13 more frequently than compound 7f and 7i (Fig. 7, B), suggesting that the higher observed reactivity results from this improved pre-orientation. However, the total population of these states with conformations close to Cys13 (distances below 0.5 nm) is in the range of 5.4 % (**7a**) 6.2 % (**7b**), 8.3 % (**7c**) and 14.1 % (**7d**) to 0.5 % (**7i**) and 0.2 % (**7f**) (Table S2), indicating that further compound optimization is still necessary and possible to further increase the reactivity. These results are thus well in line with the high reversible affinity of the modified nucleotides while at the same time being slow to react with the thiol group to form the covalent bond. As shown in Figure S7, key differences in the binding modes of non-cyclic ligand **7a** and cyclic ligands **7b**–**7i** were revealed through analysis of their intermolecular interaction profiles, with **7a** forming numerous but low-frequency contacts - likely due to its flexible warhead - while the more rigid cyclic ligands engaged in fewer but higher-frequency interactions, indicating slightly greater binding specificity and complex stability.

### Conclusion and outlook

In this study, we synthesized a focused library of covalently binding nucleotide analogs featuring cyclic diamine linkers to systematically investigate how linker rigidity and geometry impact reactivity with the Cys13 residue in mutant KRAS. The compounds were shown to disrupt KRAS activity by inhibiting guanine nucleotide exchange factor (GEF)-catalyzed nucleotide exchange and blocking effector binding, effectively stabilizing the protein in its inactive state. While these new analogs did not exhibit enhanced reactivity compared to the parent molecules, structural characterization using X-ray crystallography and *in silico* modeling provided new insights and revealed considerable conformational flexibility, explaining the low reactivity at physiological pH. Compared to the previously published compound **7a** containing a short linear ethylendiamine linker, our X-ray crystallographic data suggest a less strained conformation of the cyclic linker in compound **7b** and reduced impact on the ribose, while retaining a similar reactivity towards Cys13. However, our *in silico* modeling, developed to predict the conformational landscape of these molecules, indicates that only a minor population of the analogs adopt conformations of the warhead near Cys13 to achieve covalent interaction. Thus, while we were not successful in obtaining more reactive analogs at this point, this approach allows us to explain the observed low reactivity at physiological pH and offers a valuable tool for guiding the future design of nucleotide-based inhibitors with optimized preorientation and reactivity profiles, providing a systematic framework for enhancing these compounds’ reactivities in a targeted manner. Various further linker motifs are currently being evaluated via this *in silico* approach and promising molecules containing both cyclic and linear aliphatic linkers, will be synthesized and tested accordingly.

## Methods

### Chemistry

All reagents and solvents were purchased from Acros, Activate Scientific, Alfa Aesar, Apollo Scientific, Merck, Sigma-Aldrich, TCI Chemicals or VWR and used without further purification. Dry solvents were purchased as anhydrous reagents from commercial suppliers. ^1^H and^13^C NMR spectra were recorded on a Bruker Avance DRX AV400 (400 and 101 MHz), AV500 (500 and 125 MHz), AV600 (600 and 151 MHz). 1H chemical shifts are reported in *δ* (ppm) as s (singlet), d (doublet), dd (doublet of doublet), t (triplet), q (quartet), m (multiplet), br (broad singlet), and app (apparent) and are referenced to the residual solvent signal: CDCl_3_ (7.26), DMSO-*d*_6_ (2.50), or CD_3_OD (3.34). ^13^C spectra are referenced to the residual solvent signal: CDCl_3_ (77.1), DMSO-*d*_6_ (39.52), or CD_3_OD (49.86). High-resolution electrospray ionization mass spectra (ESI-FTMS) were recorded on a Thermo LTQ Orbitrap (high-resolution mass spectrometer from Thermo Electron) coupled to an Accela HPLC system supplied with a Hypersil GOLD column (Thermo Electron). LCMS (ESI-MS) analysis was performed using an Agilent HPLC system (1100 series) with a CC 125/4 Nucleodur C18 gravity column (3 μm) from Macherey Nagel coupled to a Thermo Scientific Finnigan LCQ Advantage Max Ion Trap and ESA Corona detector. Compounds were purified by flash chromatography on a Biotage Isolera One using Büchi Reveleris Silica Cartridges (4–120 g) monitored by UV at λ = 210 and 280 nm. Unless otherwise noted, all final products were synthesized and used as racemic mixtures of the corresponding *trans*-isomers.

*General Procedure A for Synthesis of Modified Amines* ***3a*** *to* ***3i*** – The Boc-protected amine (1.0 equiv) was dissolved in tetrahydrofuran (THF, c = 0.5 M) and treated with N, N-diisopropylethylamine (DIPEA, 2.0 equiv). The solution was cooled to 0 °C in an ice bath. A solution of acryloyl chloride (1.0 equiv) in THF (c = 2.3 M) was then added dropwise with caution to the stirred mixture under cooling. The reaction was allowed to proceed for 4 h at 0 °C. Upon completion, the mixture was extracted three times with dichloromethane (DCM) and an aqueous solution of sodium bicarbonate (3 × 50 mL). The combined organic layers were dried and concentrated under reduced pressure.

The crude product was redissolved in DCM (3 parts), cooled to 0 °C, and treated dropwise with trifluoroacetic acid (TFA, 1 part). The reaction mixture was stirred on ice for 30 min. Afterward, the solution was neutralized with 10 M NaOH, and the solvent was removed under reduced pressure. The residue was purified by automated flash column chromatography on silica gel using DCM/MeOH containing 1% NH₃ to yield the desired acrylamide product.

*General Procedure B for Protection of GDP* ***5*** – Using a strongly acidic cation exchanger (Ion Exchanger I from Merck), the nucleotide was initially converted into a DMF-soluble form. Therefore, the pre-swollen column material was loaded into a column with a diameter of 2 cm and a packing height of 15 to 20 cm and then incubated for one hour in a pyridine/water mixture (1:1). After washing the column material with a total of 250 mL of distilled water, a solution of Guanosine diphosphate (GDP, C_10_H_15_N_5_O_11_P_2_, 443.2 g/mol, 100 mg, 0.5 mmol, 1 equiv.) in distilled water (3 mL, *c* = 0.167 M) was applied to the column using a Pasteur pipette. The nucleotide was eluted with a mixture of methanol/water (1:1) into a round-bottom flask preloaded with tetrabutylammonium hydroxide (TBAH, C_16_H_37_NO, 259.48 g/mol, *c* = 1 M in CH_3_OH, 1 mL, 260 mg, 1 mmol, 2 equiv.). After removing the solvent mixture using a rotary evaporator, the residue was treated three times with 20 mL of DMF each and then concentrated again. The residue was redissolved in DMF (20 mL, *c* = 25 mM), and 1,1’-Carbonyldiimidazol (CDI, C_7_H_6_N_4_O, 162.15 g/mol, 405 mg, 2.5 mmol, 5 equiv.) was added at 0 °C under argon. The reaction mixture was then stirred overnight at 0 °C and upon completion of the reaction, it was quenched by adding 150 µL of methanol. The compound formed in this reaction was used directly in the subsequent reaction without further purification.

*General Procedure C for Coupling the modified Amine* ***3a*** *to* ***3i*** *to GDP* ***6a*** *to* ***6i*** –The amine (***3a*** *− 3****i***, 2.5 mmol, 5 equiv.) was dissolved in dry DMF (5 mL, *c* = 0.5 M), and slowly added to a solution of GDP ***5*** (*c* = 25 mM in DMF–CH_3_OH (134:1), 1 equiv.) and N, N-Diisopropylethylamine (DIPEA, C_8_H_19_N, 129.25 g/mol, 0,76 g/mL, 2.5 mmol, 5 equiv). The reaction solution was stirred overnight, upon which it became cloudy and inhomogeneous. The resulting precipitate was centrifuged at 10,000 rpm for 10 min and then washed twice with 30 mL of dry DMF each, followed by centrifugation again. Finally, the pellet was dissolved in 30 mL of distilled water. The products formed during the reaction were used directly in the subsequent reaction without further purification.

*General Procedure D for Deprotection of GDP* ***6a*** *to* ***6i*** *to GDP* ***7a to 7i*** – The solution of *GDP* ***6a*** *to* ***6i*** was adjusted to a pH of 1.5 by carefully adding an aqueous solution of HCl (*c* = 0.25 M) and then stirred overnight at 4 °C. Following the completion of the reaction, the pH was adjusted to 7.5 by adding an aqueous solution of NaOH (*c* = 0.25 M), before diluting the nucleotide solution to a total volume of 100 mL with milliQ H_2_O. The sample was filtered using a syringe filter and subjected to purification via a Q-Sepharose column on the FPLC system. After loading the nucleotide solution onto the column equilibrated with Buffer A (50 mM TEAB), elution was performed at a flow rate of 1 mL/min using a linear gradient of 0–100% Buffer B (1 M TEAB) over 600 min. The collected fractions were analyzed by HPLC using TBAB as the counterion in the running buffer (50 mM KPi pH 6.6, 10 mM TBAB, 16% ACN; column: ProntoSIL^®^ 120-5-C18-AQ, Bischoff), and fractions containing the product were combined. Following the removal of the solvent mixture using a rotary evaporator, the residue was dissolved in 10 mL milliQ H_2_O and lyophilized.

*N*-(2-aminoethyl)acrylamide **3a**. According to the general procedure A, 100 mg of *tert*-butyl (2-aminoethyl)carbamate (0.62 mmol, 1 equiv) was reacted. **3a** was obtained after purification by column chromatography in a yield of 68.51 mg (0.59 mmol, 96%). ^**1**^**H NMR** (600 MHz, CD_3_OD) *δ* 6.26 (app d, J = 0.9 Hz, 1 H), 6.25 (app br. s, 1 H), 5.71 (t, J = 6.0 Hz, 1 H), 3.53 (t, J = 6.0 Hz, 1 H), 3.27 (s, 2 H), 3.09 (t, J = 6.0 Hz, 1 H); ^**13**^**C NMR** (151 MHz, CD_3_OD) *δ* 131.8, 127.6, 40.9, 38.4, 38.1;. The analytical data for amine 3a was in agreement with the previously published analytic data.

(*R*)-*N*-(pyrrolidin-3-yl)acrylamide **3f**. According to the general procedure A, 100 mg of *tert-*butyl (*R*)−3-aminopyrrolidine-1-carboxylate (0.53 mmol, 1 equiv) was reacted. **3f** was obtained after purification by column chromatography in a yield of 36 mg (0.26 mmol, 48.5%).^**1**^**H NMR** (600 MHz, CD_3_OD) *δ* 6.26 (app s, 1 H), 6.25 (app d, J = 1.9 Hz, 1 H), 5.70 (dd, J = 5.0, 6.9 Hz, 1 H), 4.48 (ddd, J = 5.0, 7.0, 11.8 Hz, 1 H), 3.54 (dd, J = 6.9, 12.2 Hz, 1 H), 3.48 (app td, J = 7.6, 11.8 Hz, 1 H), 3.38 (ddd, J = 6.5, 8.2, 11.8 Hz, 1 H), 3.27 (dd, J = 4.7, 12.2 Hz, 1 H), 2.34 (ddd, J = 7.3, 14.0, 15.2 Hz, 1 H), 2.06 (app ddt, J = 1.4, 6.8, 13.2 Hz, 1 H); ^**13**^**C NMR** (151 MHz, CD_3_OD) *δ* 168.5, 131.6, 127.7, 56.0, 51.3, 50.5, 45.7, 31.0; **LC-MS** (ESI-MS): [*t*_R_] = 1.6 min, [M + H]^+^ calcd. for C_7_H_13_N_2_O: 141.1; found 141.1.

(*S*)-*N*-(pyrrolidin-3-yl)acrylamide **3 g**. According to the general procedure A, 100 mg of *tert-*butyl (*S*)−3-aminopyrrolidine-1-carboxylate (0.53 mmol, 1 equiv) was reacted. **3 g** was obtained after purification by column chromatography in a yield of 48 mg (0.34 mmol, 64.6%). ^**1**^**H NMR** (600 MHz, CD_3_OD) *δ* 6.26 (app s, 1 H), 6.25 (app d, J = 2.5 Hz, 1 H), 5.70 (dd, J = 4.7, 7.2 Hz, 1 H), 4.48 (ddd, J = 5.0, 7.2, 12.4 Hz, 1 H), 3.54 (dd, J = 6.9, 12.3 Hz, 1 H), 3.48 (app td, J = 7.6, 11.8 Hz, 1 H), 3.37 (ddd, J = 6.5, 8.2, 11.8 Hz, 1 H), 3.26 (dd, J = 4.7, 12.2 Hz, 1 H), 2.34 (ddd, J = 7.0, 14.0, 15.0 Hz, 1 H), 2.06 (app ddt, J = 1.2, 6.7, 13.3 Hz, 1 H); **13 C NMR** (151 MHz, CD_3_OD) *δ* 168.5, 131.6, 127.7, 55.8, 51.3, 50.5, 45.8, 31.0; **LC-MS** (ESI-MS): [*t*_R_] = 1.3 min, [M + H]^+^ calcd. for C_7_H_13_N_2_O: 141.1; found 141.4.

*N*-(2-aminocyclohexyl)acrylamide **3e**. According to the general procedure A, 100 mg of *tert*-butyl (2-aminocyclohexyl)carbamate (0.46 mmol, 1 equiv) was reacted. **3e** was obtained after purification by column chromatography in a yield of 32.8 mg and as a racemic mixture of *cis*- and *trans*-diastereomers (0.19 mmol, 42.4%). The diastereomeric ratio (dr) was determined by ^1^H NMR spectroscopy based on the integration of the olefinic proton signals at *δ* = 5.63 ppm and *δ* = 5.61 ppm, showing a dr of 9:10. Due to concentration limitations and the diastereomeric mixture, no reliable purity testing could be performed using 1H-NMR. In this regard, identity verification by LC/MS for **amine 3e** and HRMS of **GDP 7e** confirmed the formation of a product with the desired m/z ratio. Due to this, only the spectra is shown in the supplementary information but no chemical shifts are provided in this part. **LC-MS** (ESI-MS): [*t*_R_] = 4.5 min, [M + H]^+^ calcd. for C_9_H_17_N_2_O: 169.1; found 169.1.

(*R*)-*N*-(piperidin-3-yl)acrylamide **3b**. According to the general procedure A, 100 mg of *tert*-butyl *(R*)−3-aminopiperidine-1-carboxylate (0.49 mmol, 1 equiv) was reacted. **3b** was obtained after purification by column chromatography in a yield of mg 61.3 mg (0.4 mmol, 81.1%). ^**1**^**H NMR** (400 MHz, CD_3_OD) *δ* 6.77 (dd, J = 12.4, 16.4 Hz, 1 H), 6.22 (dd, J = 1.5, 16.81 Hz, 1 H), 5.78 (dd, J = 1.8, 10.6 Hz, 1 H), 4.30 (app d, J = 8.4 Hz, 1 H), 3.94–4.19 (m, 1 H), 3.84 (app d, J = 14.8 Hz, 1 H), 3.34–3.52 (m, 2 H), 2.05–2.19 (m, 1 H), 1.85 (app br. s, 1 H), 1.52–1.79 (m, 2 H); The^13^**C NMR** signals were in accordance with the spectral data of **amine 3c LC-MS** (ESI-MS): [*t*_R_] = 1.8 min, [M + H]^+^ calcd. for C_8_H_15_N_2_O: 155.1; found 155.1.

(*S*)-*N*-(piperidin-3-yl)acrylamide **3c**. According to the general procedure A, 100 mg of *tert*-butyl *(S*)−3-aminopiperidine-1-carboxylate (0.49 mmol, 1 equiv) was reacted. **3c** was obtained after purification by column chromatography in a yield 68.8 mg (0.4 mmol, 91.1%). ^**1**^**H NMR** (700 MHz, CD_3_OD) *δ* 6.77 (dd, J = 11.2, 15.9 Hz, 1 H), 6.22 (app d, J = 16.8 Hz, 1 H), 5.78 (app d, J = 10.5 Hz, 1 H), 4.31 (app br. s., 1 H), 4.07 (app br. s, 1 H), 3.84 (app d, J = 13.3 Hz, 1 H), 3.39 (app br. s., 1 H), 3.27 (app br. s., 1 H), 2.07–2.18 (m, 1 H), 1.85 (app br. s., 1 H), 1.67–1.76 (m, 1 H), 1.62 (app br. s., 1 H); ^**13**^**C NMR** (151 MHz, MeOD) δ 168.3, 131.6, 127.7, 51.3, 50.5, 45.8, 31.0; **LC-MS (ESI-MS)**: [*t*_R_] = 1.4 min, [M + H]^+^ calcd. for C_8_H_15_N_2_O: 155.1; found 155.1.

*N*-(piperidin-4-yl)acrylamide hydrochloride **3 h**. According to the general procedure A, 100 mg of *tert-*butyl 4-aminopiperidine-1-carboxylate (0.49 mmol, 1 equiv) was reacted. Instead of using TFA for deprotection, a solution of HCl in 1,4-Dioxan (*c* = 4 M, 5 equiv) was used, whereas **3i** could be obtained after purification by column chromatography as a hydrochloride salt in a yield of 61.3 mg (0.4 mmol, 80%). ^**1**^**H NMR** (500 MHz, DMSO-d_6_) *δ* 8.59 (br. s., 1 H), 8.42 (br. s., 1 H), 8.23 (d, J = 7.3 Hz, 1 H), 6.20 (dd, J = 10.1, 17.1 Hz, 1 H), 6.10 (dd, J = 2.2, 17.0 Hz, 1 H), 5.60 (dd, J = 2.4, 10.0 Hz, 1 H), 3.90 (dddd, J = 3.6, 7.2, 7.4, 11.0 Hz, 1 H), 3.26 (app d, J = 13.0 Hz, 2 H), 3.00 (app dd, J = 10.8, 22.8 Hz, 2 H), 1.92 (app dd, J = 3.1, 13.8 Hz, 2 H), 1.56 (ddd, J = 3.8, 11.8, 24.4 Hz, 2 H); ^**13**^**C NMR** (126 MHz, DMSO) *δ* 164.0, 131.5, 125.6, 43.4, 42.1, 28.2; **LC-MS** (ESI-MS): [*t*_R_] = 1.2 min, [M + H] + calcd. for C_8_H_15_N_2_O: 155.1; found 155.1.

1-(4-aminopiperidin-1-yl)prop-2-en-1-one **3i**. According to the general procedure A, 100 mg of *tert-*butyl 4-aminopiperidine-1-carboxylate (0.49 mmol, 1 equiv) was reacted. **3 h** was obtained after purification by column chromatography in a yield of 61.3 mg (0.39 mmol, 80%). ^**1**^**H NMR** (700 MHz, CD_3_OD) *δ* 6.78 (dd, J = 10.8, 16.8 Hz, 1 H), 6.20 (dd, J = 1.5, 16.9 Hz, 1 H), 5.76 (dd, J = 1.8, 10.7 Hz, 1 H), 4.63 (app d, J = 12.7 Hz, 1 H), 4.21 (app d, J = 13.6 Hz, 1 H), 3.41 (app t, J = 12.4 Hz, 1 H), 3.22 (tt, J = 3.9, 12.6 Hz, 1 H), 2.80 (app t, J = 12.7 Hz, 1 H), 2.09 (app t, J = 13.3 Hz, 2 H), 1.52 (app dt, J = 11.3, 11.8 Hz, 2 H); ^**13**^**C NMR** (176 MHz, MeOD) *δ* 167.8, 129.0, 129.0, 56.0, 45.2, 41.6, 31.8, 30.9; **LC-MS (ESI-MS)**: [*t*_R_] = 1.2 min, [M + H] + calcd. for C_8_H_15_N_2_O: 155.1; found 155.1.

(*R*)-*N*-(azepan-3-yl)acrylamide **3d**. According to the general procedure A, 100 mg of *tert-*butyl 4-aminopiperidine-1-carboxylate (0.47 mmol, 1 equiv) was reacted. **3d** was obtained after purification by column chromatography in a yield of 62 mg (0.4 mmol, 78.5%). ^**1**^**H NMR** (600 MHz, CD_3_OD) *δ* 6.26 (app s, 1 H), 6.25 (app d, J = 1.8 Hz, 1 H), 5.70 (dd, J = 5.1, 6.9 Hz, 1 H), 4.15 (app tt, J = 4.4, 9.1 Hz, 1 H), 3.40 (ddd, J = 0.7, 3.9, 13.6 Hz, 1 H), 3.35 (s, 1 H), 3.29 (app dd, J = 4.6, 6.2 Hz, 1 H), 3.18–3.23 (m, 2 H), 2.03–2.11 (m, 1 H), 1.95–2.02 (m, 1 H), 1.84–1.95 (m, 2 H), 1.73–1.81 (m, 1 H), 1.61–1.70 (m, 1 H); ^**13**^**C NMR** (151 MHz, MeOD) *δ* 167.9, 131.7, 127.7, 50.5, 49.0, 48.5, 33.7, 26.3, 23.7; **LC-MS** (ESI-MS): [*t*_R_] = 2 min, [M + H] + calcd. for C_9_H_17_N_2_O: 169.1; found 169.1.

CDI-protected GDP **5**. According to General Procedure B, **5** was obtained after purification. LC-MS (*m/z*): Calculated for C_14_H_14_N_7_O_11_P_2_^−^: 518.0 [M-H]^−^, found: 518.0.

According to the General Procedure C, the modified amines (1.2 mmol, 2 equivalents) were reacted with **5** in the presence of DIPEA (0.5 ml, 3.0 mmol, 5 equivalents). The following products were obtained (Table 1):

**Table 1:** Overview of the GDP-modified compounds obtained according to general procedure C and the observed *m/z* values.

Nucleotide		#	Exact Mass (calc.)	for [M-H]^−^	LC-MS (m/z)
		**6a**	678.1	C_21_H_30_N_9_O_13_P_2_^−^	677.9
		**6f**	730.1	C_25_H_34_N_9_O_13_P_2_^−^	729.9
		**6 g**	730.1	C_25_H_34_N_9_O_13_P_2_^−^	730.1
		**6e**	786.2	C_29_H_42_N_9_O_13_P_2_^−^	786.1
		**6b**	758.2	C_27_H_38_N_9_O_13_P_2_^−^	758.1
		**6c**	758.2	C_27_H_38_N_9_O_13_P_2_^−^	758.1
		**6 h**	758.2	C_27_H_38_N_9_O_13_P_2_^−^	758.1
		**6i**	758.2	C_27_H_38_N_9_O_13_P_2_^−^	758.0
		**6d**	786.2	C_29_H_42_N_9_O_13_P_2_^−^	786.1

According to the General Procedure D, molecules **6a**–**i** were reacted. The following products were obtained (Table 2):

**Table 2:** Overview of the GDP-modified compounds obtained according to General Procedure D and the observed *m/z* values.

Nucleotide		#	Exact Mass (calc.)	for [M-H]^−^	LC-MS (m/z)	HRMS (m/z) [M + H]^+^
		**7a**	582.0756	C_16_H_22_N_7_O_13_P_2_^−^	581.9	
		**7f**	608.0913	C_18_H_24_N_7_O_13_P_2_^−^	608.0	
		**7 g**	608.0931	C_18_H_24_N_7_O_13_P_2_^−^	608.0	
		**7e**	636.1226	C_20_H_28_N_7_O_13_P_2_^−^	636.0	
		**7b**	622.1069	C_19_H_26_N_7_O_13_P_2_^−^	622.0	624.1220
		**7c**	622.1069	C_19_H_26_N_7_O_13_P_2_^−^	622.1	624.1219
		**7 h**	622.1069	C_19_H_26_N_7_O_13_P_2_^−^	622.0	
		**7i**	622.1069	C_19_H_26_N_7_O_13_P_2_^−^	622.0	
		**7d**	636.1226	C_20_H_28_N_7_O_13_P_2_^−^	636.0	638.1373

All corresponding LC/MS, HRMS and NMR spectra for the synthetic part are available in the Supplementary Information (Figures S9 – S45).

### Biological experiments

#### Protein expression and purification

KRAS^G13C^_1−169_ Cys-light (C51S C80L C118S) were expressed in *E. coli* BL21 (DE3) at 37 °C. Protein expression was induced at A600 nm of 0.5 by the addition of 0.2–0.3 mM isopropyl-b-D-thiogalactoside (IPTG), and growth was continued at 19 °C overnight. The bacteria were collected by centrifugation, and the obtained pellet resuspended in Ni-NTA buffer (50 mM Tris pH 8.0, 250 mM NaCl, 40 mM imidazole, 4 mM MgCl_2_, 10 µM GDP, 1 mM Tris(2-chlorehyl)phosphate (TCEP), and 5 % glycerol). The cells were lysed with a microfluidizer, and after addition of protease inhibitor cocktail (Roche complete EDTA free) and 1 % CHAPS (w/v) stirring was continued for 1 h at 4 °C. The lysate was cleared by centrifugation (35,000 x g, 1 h, 4 °C), and the supernatant was loaded onto a Ni-affinity chromatography column (Qiagen Ni-NTA Superflow, 5 mL) pre-equilibrated with Ni-NTA buffer. The protein was eluted with a linear gradient of imidazole buffer (40 mM – 500 mM). For cleavage of the N-terminal hexahistidine-tag, TEV protease was added to the pooled elution fractions and dialyzed overnight into dialysis buffer at 4 °C (25 mM Tris pH 8.0, 100 mM NaCl, 4 mM MgCl_2_, 10 µM GDP, 1 mM TCEP, and 5 % glycerol). The cleaved protein was then applied to a reverse Ni-affinity chromatography column. The eluted protein fractions were concentrated to around 10 mL followed by dilution with salt-free AEX buffer (25 mM Tris pH 8.0, 2 mM MgCl_2_, 10 µM GDP, 1 mM TCEP, and 5 % glycerol) for better binding conditions onto AEX column (Cytiva, QFF HiTrap, 2 × 1 mL). The protein was eluted with a linear gradient (0 mM – 1000 mM NaCl). Finally, the protein was purified by size-exclusion chromatography (GE HiLoad 16/60 Superdex 75 pg) in a final buffer containing 20 mM HEPES pH 7.5, 100 mM NaCl, 2 mM MgCl_2_, 10 µM GDP, 1 mM TCEP, and 5 % glycerol.

### Covalent modification of proteins

To assess the extent of covalent modification of KRAS^G13C^_1–169_, 50 µM Ras protein was incubated with a 10-fold molar excess of acryl-bearing nucleotides in a buffer containing 100 mM HEPES (pH 7, 7.5 and 8) or 100 mM CHES (pH 8.5, 9 and 9.5), 50 mM NaCl, 1 mM TCEP, 5% glycerol and 1 mM EDTA. After incubation at room temperature for the appropriate duration, the extent of protein modification was analyzed by ESI-MS. MS spectra were acquired on a VelosPro IonTrap mass spectrometer (Thermo Scientific) using an AdvanceBio Desalting-RP, 2.1 × 2.5 mm column (Agilent Technologies). A gradient elution was employed, transitioning from mobile phase A (0.1% formic acid in water) to mobile phase B (0.1% formic acid in acetonitrile). The software MagTran was used for deconvolution.

### Effector binding (pull-down assays)

Pull-down experiments were performed using a buffer containing 20 mM HEPES (pH 7.5), 50 mM NaCl, and 2 mM MgCl₂. For each assay, 10 µg KRAS^G13C^:GDP or covalently modified KRAS^G13C^-**7a** and **7b** and 20 µg GST-tagged cRaf-RBD (amino acids 51–131) were incubated either in the presence or absence of 100 µM GppNHp and 1 µg SOS overnight at room temperature. Following incubation, 50 µL of glutathione magnetic beads were added to each sample and incubated for 30 min. Beads were then washed with 500 µL buffer, separated using a magnetic rack, and the supernatant was carefully removed. The beads were resuspended in 50 µL of 4x SDS-loading buffer, and samples were analyzed by SDS-PAGE.

### Preparative modification of RAS

The KRAS^G13C^ protein was diluted to 2 mg/mL and incubated with a 3-fold excess of the acryl-bearing nucleotide in 100 mM CHES (pH 9.5), 50 mM NaCl, 1 mM EDTA, 1 mM TCEP, and 5% glycerol at room temperature. After incubation for 24 h, the protein modification was controlled by ESI-MS. The MS spectra were recorded on an VelosPro IonTrap (Thermo Scientific) with an AdvanceBio Desalting-RP, 2.1 × 2.5 mm column (Agilent Technologies) and a gradient of the mobile phase A (0.1 % formic acid in water) to B (0.1 % formic acid in acetonitrile). The protein: inhibitor complex was purified by size-exclusion chromatography (Superdex Increase 75 pg 10/300 GL) in a final buffer containing 20 mM HEPES pH 7.5, 100 mM NaCl, 2 mM MgCl2, 5 mM TCEP, and 5 % glycerol to remove excess of nucleotides.

### Guanine nucleotide-exchange factor assay

SOS-catalyzed nucleotide exchange was measured at 25 °C using a FluoroMax-3 spectrofluorometer. Fluorescence was monitored with an excitation wavelength of 360 nm and an emission wavelength of 440 nm. The assay buffer consisted of 20 mM HEPES (pH 7.5), 100 mM NaCl, 2 mM MgCl₂, and 1 mM TCEP. In the assay, 5 µM of the preparatively modified or unmodified KRAS (residues 1–169) was incubated with 10 µM mant-dGDP, followed by the addition of SOS at varying concentrations (0.25 µM and 0.5 µM; black and red curves, respectively). Fluorescence changes were recorded to monitor nucleotide exchange.

### Crystallization

The KRAS^G13C^ protein was diluted to 2 mg/mL and incubated with a 3-fold excess of the acryl-bearing nucleotide **7b** in 100 mM CHES (pH 9.5), 50 mM NaCl, 1 mM EDTA, 1 mM TCEP, and 5 % glycerol at room temperature. After incubation for 24 h, the protein modification was controlled by ESI-MS. The MS spectra were recorded on an VelosPro IonTrap (Thermo Scientific) with an AdvanceBio Desalting-RP, 2.1 × 2.5 mm column (Agilent Technologies) and a gradient of the mobile phase A (0.1 % formic acid in water) to B (0.1 % formic acid in acetonitrile). The protein: inhibitor complex was purified by size-exclusion chromatography (Superdex Increase 75 pg 10/300 GL) in a final buffer containing 20 mM HEPES pH 7.5, 100 mM NaCl, 2 mM MgCl2, 5 mM TCEP, and 5 % glycerol, and subsequently concentrated to 44 mg/mL and 28 mg/mL and used for crystallization.

A former identified initial crystallization condition (JCSG Core III, B5) was further optimized and used for crystallization at 4, 12 and 18 °C. The protein: inhibitor complex was mixed in a 1:1 ratio (1 µL protein: inhibitor complex and 1 µL reservoir solution containing 100 mM sodium acetate pH 8.0–9.0, 200 mM Tris-HCl pH 8.0–9.0, 25–35 % (w/v) PEG4000). The crystals grew within 24 h with the higher protein concentration and within weeks with the lower concentration. The fast-grown crystals were much more intergrown; therefore, the slow-grown crystals were used for the following shrinking step. The crystals were transferred in a new drop containing SEC buffer condition and crystallization conditions mixed in a 1:1 ratio plus an additional 30 % PEG3350/PEG4000, 20% glycerol, 20 % ethylene glycol, or 20 % PEG 400 for 24 h at 18 °C. After incubation, the crystals were fished and flash cooled in liquid nitrogen. The data sets were collected at the ID30B beamline of the ESRF (European Synchrotron Radiation Facility, Grenoble, France, DOI: 10.15151/ESRF-ES-1581727707) The data were processed using XDS and scaled using XSCALE.

### Structure determination and refinement

The complex crystal structure was solved by molecular replacement with PHASER using structure PDB ID: 7ok3 as template^40^. The molecules in the asymmetric units were manually adjusted using the program COOT^41^. The refinement was performed with Phenix.refine 1.21.1^42^. Inhibitor topology files were generated using eLBOW of the Phenix 1.21.1 program package. Refined structures were validated with the PDB validation server. Data collection, structure refinement statistics, PDB-ID codes, and further details for data collection are provided in Table 3. PyMOL (W.L. DeLano, The PyMOL Molecular Graphics System) was used for generating the figures.

**Table 3:** Data statistics for KRAS^G13C^ covalently bound to compound **7b** (PDB ID 9I7Y).

Data collection	
Space group	*C* 1 2 1(5)
Cell dimensions	
a, b, c [Å]	70.01, 84.76, 88.77
α, β, γ [°]	90.0, 113.26, 90.0
Resolution [Å]	50.00-1.85.00.85.00.85.00.85.00.85 (1.90–1.85)
R_meas_ [%]	4.6 (126.9)
*I*/σ*I*	18.37 (1.37)
Completeness [%]	99.8 (99.7)
CC_1/2_	99.9 (61.8)
Redundancy	6.86 (6.43)
Refinement	
Resolution [Å]	42.38–1.85 (1.90–1.85)
No. reflections	40,651
R_work_/R_free_	18.98/22.78 (55.79/54.51)
No. atoms	
Protein	chain A = 1249 chain B = 1247 chain C = 1140 total: 3636
Ligand	3 × 41
Ions (Mg^2+^)	3
Water	128
*B*-factors	
Protein	chain A = 56.28 chain B = 62.13 chain C = 61.55
Ligand	chain A = 52.72 chain B = 52.18 chain C = 62.00
Ions (Mg^2+^)	52.85
Water	55.83
R.m.s. deviations	
Bond lengths [Å] Bond angles [°]	0.011 1.249
Ramachandran [%]	
Outliers Allowed Favored	0 1.75 98.25
Rotamer [%]	
Outliers Allowed Favored	0.78 2.85 96.37

### Molecular modeling

In the present work, we hypothesized that the availability of ligand conformations in close proximity to Cys13 would relate to the inhibitory efficiency of the compounds. In this sense, the crystallographic structure of KRAS^G13C^ covalently bound to inhibitor 7a was retrieved from PDB under ID 7ok3, and used as a basis for the following steps of the study. Compound 7a was removed from the crystallographic complex and used to model compounds 7b-I using Maestro (Maestro version 2024.1, Schrödinger, LLC, New York, NY, 2024). The molecules were submitted to an energy minimization step, which was performed by 75 iterations using the OPLS4 force field, and used as inputs for molecular docking to the target enzyme. After an accurate redocking of 7a, each compound was docked to KRAS^G13C^using DockThor web-server^38^, maintaining the ligand’s flexibility. To access the solution ensemble of the reversible states of compounds **7a**-**i** and explore the proximity of the warhead to Cys13, each docked complex was submitted to molecular dynamics (MD) simulations using GROMACS 2019.4^43^ and CHARMM36 force field^44^. The ligand topology was generated using the Ligand Reader & Modeler tool available on the CHARMM-GUI web server. Each complex was prepared using the Solution Builder tool available on the CHARMM-GUI web server^45^, inserted in cubic boxes with a minimum distance of 10 Å between the solute and the box edges, solvated with TIP3P water molecules, and neutralized with a 0.15 M NaCl concentration. Simulations were carried out at physiological pH and temperature (298 K) using an integration step of 2 fs. The LINCS algorithm was used to constrain the lengths of hydrogen bonds, while the PME method was used to calculate the long-range electrostatic interactions. The V-rescale thermostat with two coupling groups was used to maintain the temperature of the system, and the Parrinello-Rahman barostat was used to maintain the pressure of the system. The systems were initially equilibrated for 1ns for a canonic ensemble (NVT) with the atomic positions restrained using a 5,000 kJ mol^−1^ force on both protein and ligand atoms. After this step, five 1 ns simulations under the isothermal–isobaric ensemble (NPT) were performed (1 atm), progressively lowering the above-mentioned force in steps of 1,000 kJ.mol^−1^. After this careful equilibration process, production runs were performed for 1 µs, each complex being simulated with two independent simulations (replicas), starting from random velocities, in order to reinforce the reproducibility of the performed simulations.

## Supplementary Information

Below is the link to the electronic supplementary material.

Supplementary Material 1

## Data Availability

The data supporting the findings of this study are available in the paper and its Supplementary Information. The crystal structure data generated in this study have been deposited in the PDB database under accession code 9I7Y (DOI: [https://doi.org/10.2210/pdb9I7Y/pdb](https:/doi.org/10.2210/pdb9I7Y/pdb)). The simulation trajectories and configuration files have been deposited in the Zenodo server under doi 10.5281/zenodo.17136854.

## Abbreviations

ATP: Adenosinetriphosphate
CADD: Computer aided drug design
CDI: Carbonyldiimidazole
DIPEA: N, N-diisopropylethylamine
DMF: Dimethylformamide
GDP: Guanosinediphosphate
GN: Guanosine nucleotide
GppNHp: Guanosine-5’-[(β,γ)-imido]triphosphate
GST: Glutathione-S-transferase
GTP: Guanosinetriphosphate
KRAS: Kirsten rat sarcoma
MD: Molecular dynamics
MS: Mass spectrometry
RBD: Ras binding domain
SOS: Son of sevenless
SWIIP: Switch-II-pocket
