# Supplementary material for: Targeting KRASG13C with cyclic linker based inhibitors to explore warhead orientation
Source: Sci Rep. 2025 Oct 31;15:38213. doi: 10.1038/s41598-025-22145-5 (PMC12578930; doi:10.1038/s41598-025-22145-5)
Supplement: Supplementary file 1 — Supplementary Material 1 [file 41598_2025_22145_MOESM1_ESM.pdf]

Supporting Information

Targeting KRAS^{G13C} with Cyclic Linker Based Inhibitors to Explore Warhead Orientation

Tonia Kirschner[1], João Rodriguez[2], Emerson Gonçalves Moreira[2], Janina Niggenaber[1], Jonas D. Warmuth[1], Hugo Verli[#2], Matthias P. Müller[#1], Daniel Rauh[#1]

[1]Department of Chemistry and Chemical Biology, TU Dortmund University and Drug Discovery Hub Dortmund (DDHD), Zentrum für Integrierte Wirkstoffforschung (ZIW), Otto-Hahn-Strasse 4a, 44227 Dortmund, Germany

[2]Programa de Pos-Graduacao em Biologia Celular e Molecular (PPGBCM), Centro de Biotecnologia, Universidade Federal do Rio Grande do Sul (UFRGS), Av. Bento Goncalves, 9500, Porto Alegre, CEP 91501-970, RS, Brazil

[#]Corresponding authors:

Daniel Rauh (daniel.rauh@tu-dortmund.de)
Matthias P. Müller (matthias3.mueller@tu-dortmund.de)
Hugo Verli (hugoverli@gmail.com)

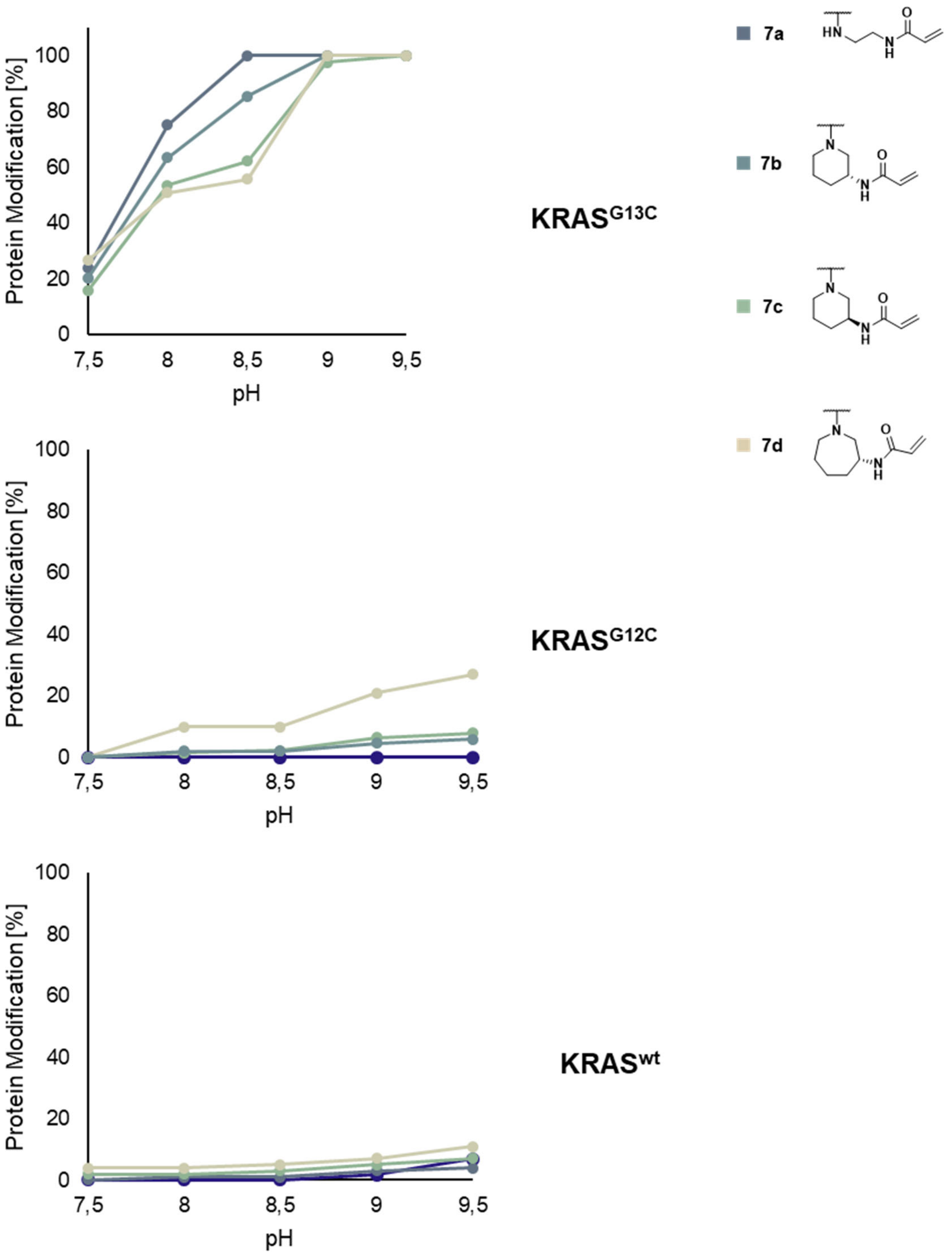

Figure S1. **Comparative analysis of modification of KRAS with the three compounds 7b–d** with the control compound eda-GDP (**7a**) was performed using the proteins $KRAS^{G13C}_{1–169}$ (Cys-light), $KRAS^{G12C}_{1–169}$ (Cys-light), and $KRAS^{wt}_{1–169}$ (from top to bottom). The covalent, percentage-based protein modification was plotted against the pH value.

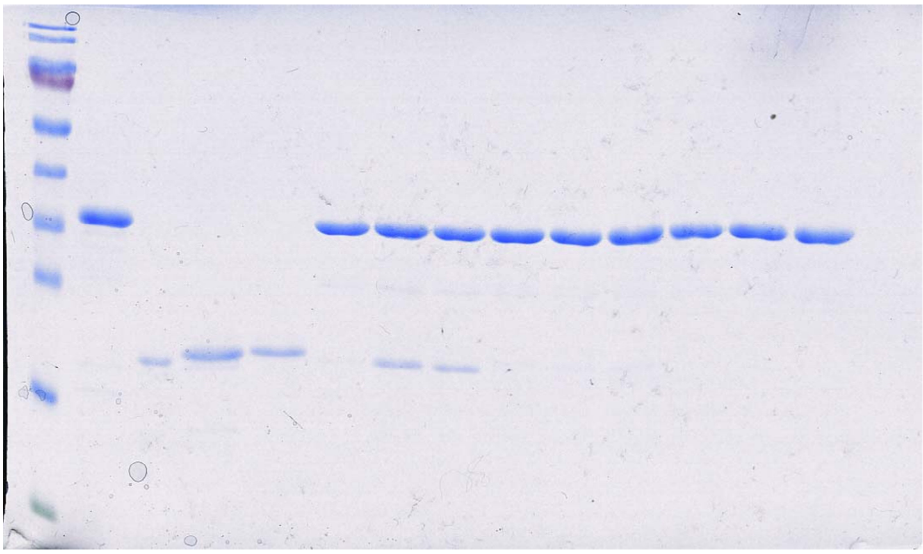

Figure S2. Unprocessed SDS gel image for Figure 5, B.

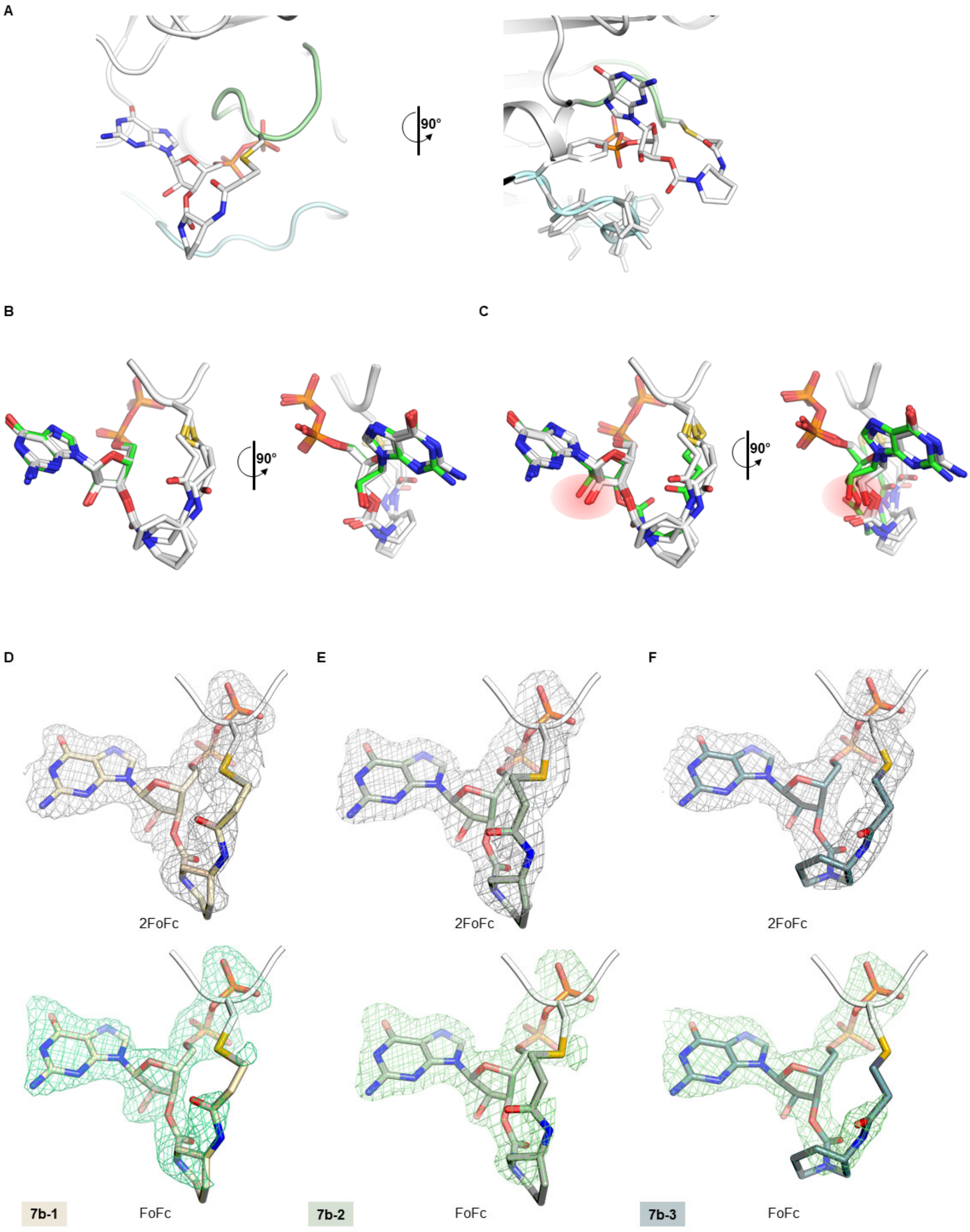

Figure S3. A: Additional rotated view compared to Figure 6 showing that the linker is solvent-exposed. **B**: Comparison of KRAS:GDP (PDB id 4OBE; GDP is shown in green) and the 3 conformations of the linker modelled in this study. **C**: Comparison of compound 7a (PDB id 7OK3; compound 7a is shown in green) and the 3 conformations of the linker. Note the slight shift of the 2'- and 3'-OH groups likely induced by the strained linker

(highlighted in red) and not observed in the panel above. **D**-**F**: Representation of the different modelled linker conformations of **7b** within the three molecules in the asymmetric unit. The 2Fo-Fc and Fo-Fc electron density map is contoured at an r.m.s.d. of 1 and 2.8, respectively. PyMOL (version 3.1.0, W.L. DeLano, The PyMOL Molecular Graphics System) was used for generating the 3D figures.

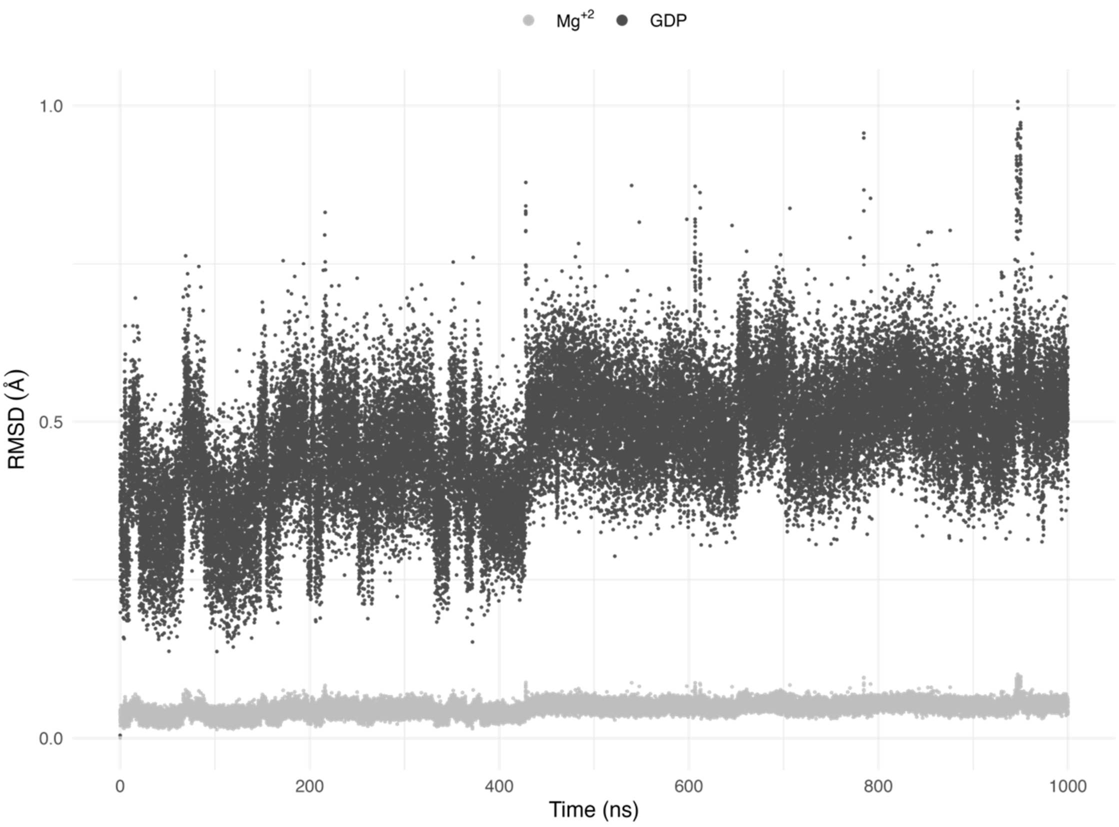

Figure S4. Stability of GDP and Mg^{2+} during MD simulations of $KRAS^{G13C}$ non-covalently bound to compound **7a** (RMSD), built based on the 7OK3 crystal structure, demonstrating the accuracy of the CHARMM36 forcefield to represent the studied molecular systems, that is, the crystallographic geometry is well maintained during simulations. The same behavior was observed for the performed MD simulations for the remaining compounds.

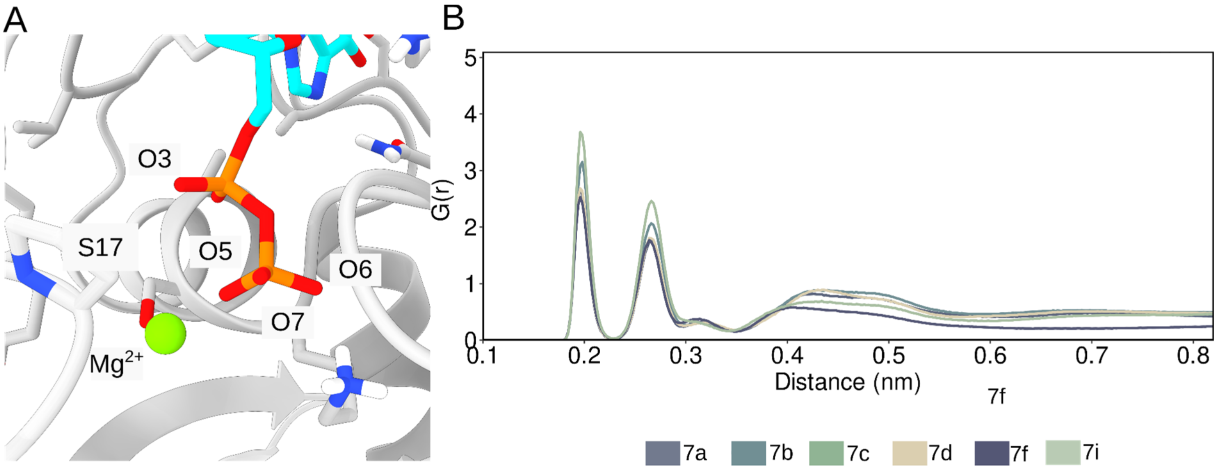

Figure S5. **A**: Interaction between Mg^{2+} and nearby atoms involved in metal coordination. **B**: Radial distribution functions, g(r), for Mg^{2+} and oxygen atoms from water molecules up to 0.8 nm distance. Number of water molecules within 0.23 nm for each complex: **7a**: 2; **7b**: 2.5; **7c**: 2; **7d**: 2; **7f**: 2; **7i**: 2.8, compared to 3 water molecules on 4OBE crystal structure. PyMOL (version 3.1.0, W.L. DeLano, The PyMOL Molecular Graphics System) was used for generating the 3D figures.

Table S1. Distance between Mg^{2+} and the atoms of nearby residues involved in the coordination, for each ligand.

**Atoms**	**Distance (nm)**						
	**PDB ID: 4OBE**	**7a**	**7b**	**7c**	**7d**	**7f**	**7i**
**Mg – PO3**	0.41	0.19±0.01	0.19±0.01	0.40±0.00	0.19±0.01	0.19±0.01	0.19±0.01
**Mg – PO5**	0.21	0.38±0.01	0.23±0.08	0.19±0.00	0.18±0.00	0.18±0.01	0.21±0.06
**Mg – PO6**	0.43	0.19±0.01	0.39±0.02	0.18±0.00	0.38±0.01	0.38±0.01	0.39±0.02
**Mg – PO7**	0.35	0.19±0.01	0.39±0.02	0.18±0.00	0.20±0.04	0.19±0.01	0.19±0.01
**Mg –Ser37**	0.20	0.21±0.01	0.33±0.02	0.33±0.13	0.21±0.01	0.20±0.01	0.39±0.14

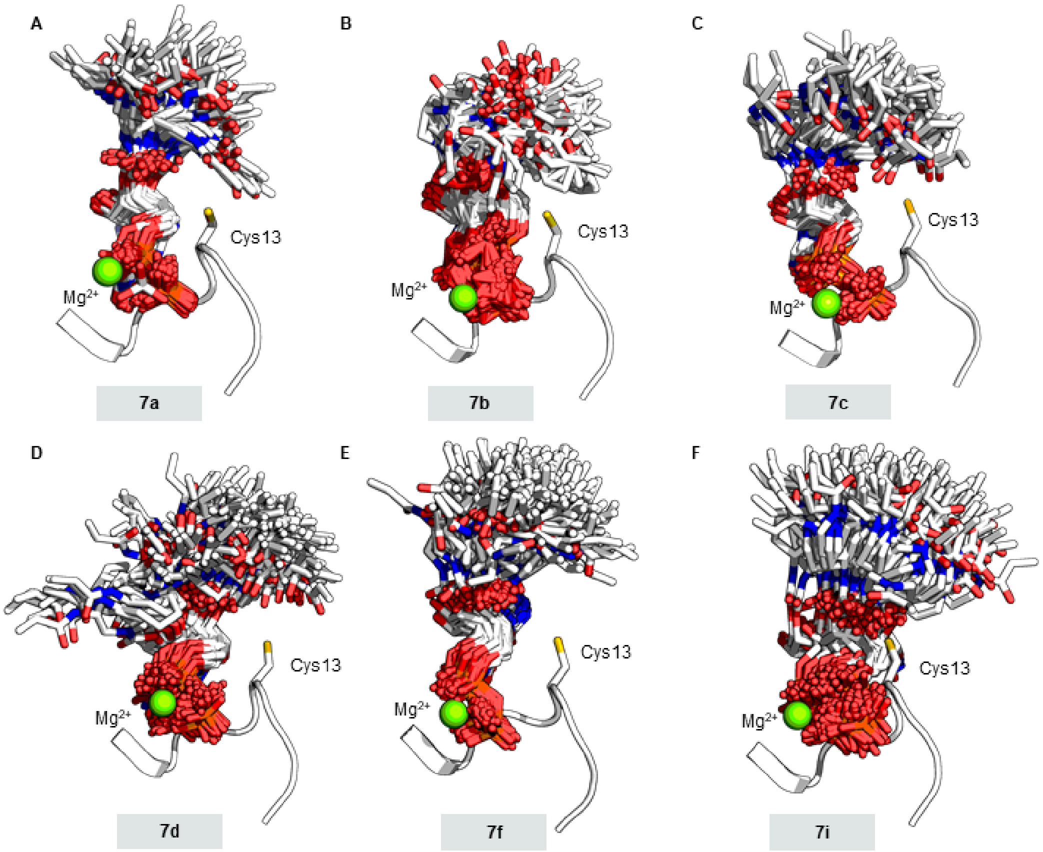

Figure S6. **A-F**: Conformational distribution of compounds **7a, b, c, d, f and i** observed in MD simulations, represented by a superimposition of conformations obtained from every 10,000 simulation steps. PyMOL (version 3.1.0, W.L. DeLano, The PyMOL Molecular Graphics System) was used for generating the 3D figures.

Table S2. Area under the curve for compounds **7a, b, c, d, f and i**, defined by a distance below to 0.5 nm Å between Cys13 and the oxygen atom from the α,β-unsaturated carbonyl group, as sampled during MD simulations.

NAME	AUC
**7a**	**5.4%**
**7b**	**6.2%**
**7c**	**8.3%**
**7d**	**14.1%**
**7f**	**0.2%**
**7i**	**0.5%**

7a

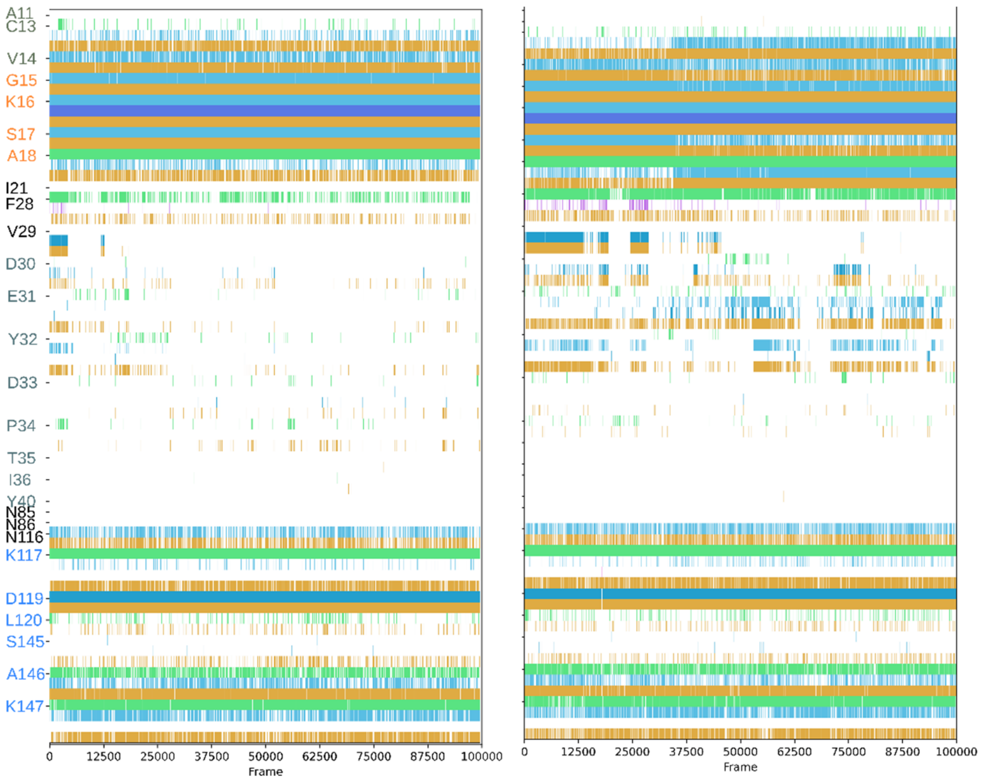
A11
C13
V14
G15
K16
S17
A18
I21
F28
V29
D30
E31
Y32
D33
P34
T35
I36
Y40
N85
N86
N116
K117
D119
L120
S145
A146
K147
0
12500
25000
37500
50000
62500
75000
87500
100000
Frame

7b

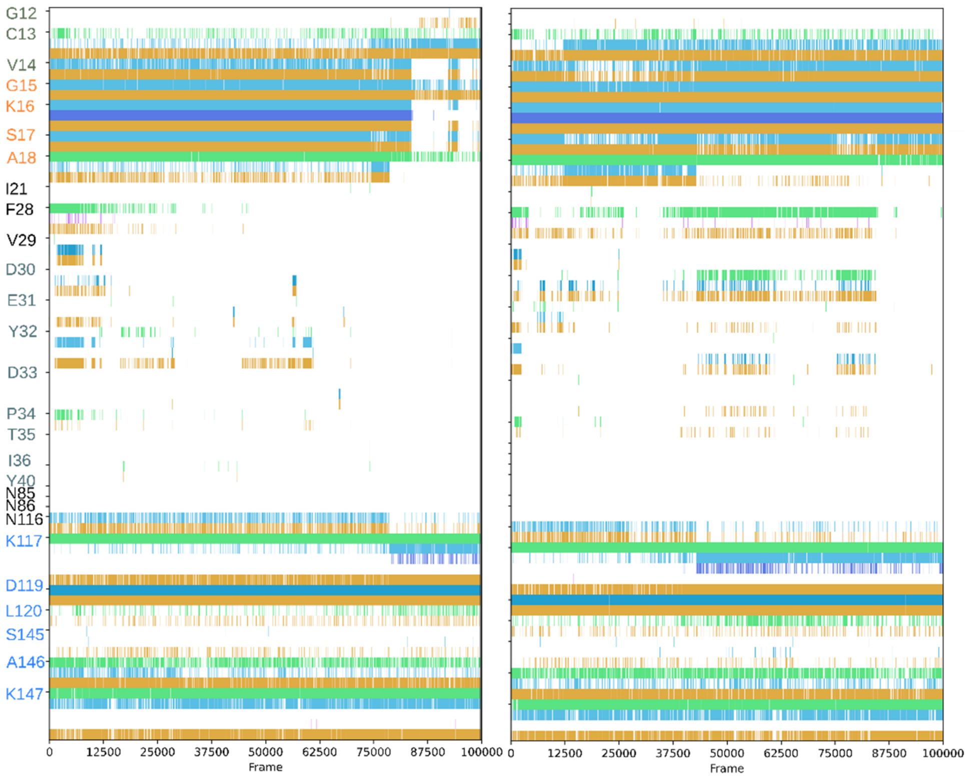
G12
C13
V14
G15
K16
S17
A18
I21
F28
V29
D30
E31
Y32
D33
P34
T35
I36
Y40
N85
N86
N116
K117
D119
L120
S145
A146
K147
0
12500
25000
37500
50000
62500
75000
87500
100000
Frame

7c

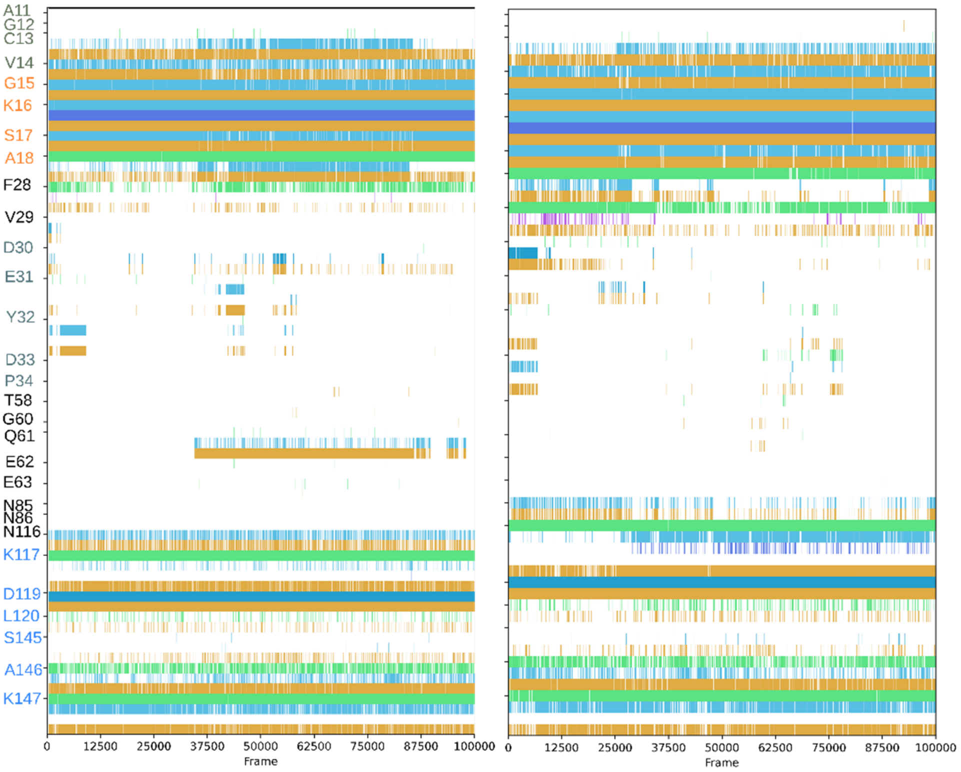
A11
G12
C13
V14
G15
K16
S17
A18
F28
V29
D30
E31
Y32
D33
P34
T58
G60
Q61
E62
E63
N85
N86
N116
K117
D119
L120
S145
A146
K147
0
12500
25000
37500
50000
62500
75000
87500
100000
Frame

7d

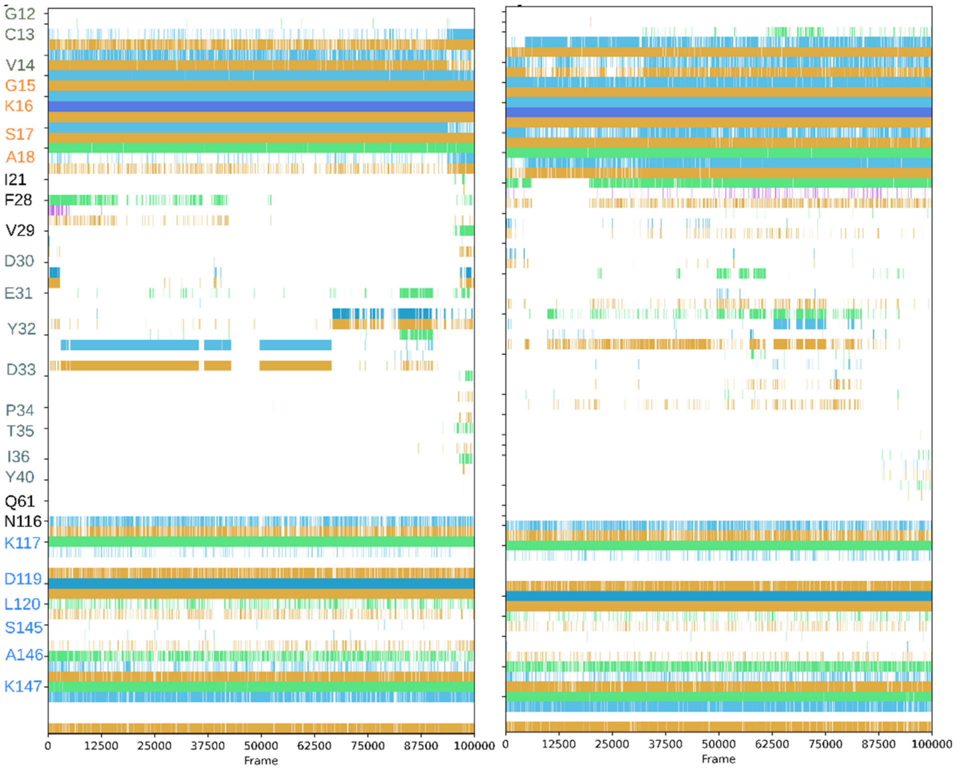
G12
C13
V14
G15
K16
S17
A18
I21
F28
V29
D30
E31
Y32
D33
P34
T35
I36
Y40
Q61
N116
K117
D119
L120
S145
A146
K147
0
12500
25000
37500
50000
62500
75000
87500
100000
Frame

7f

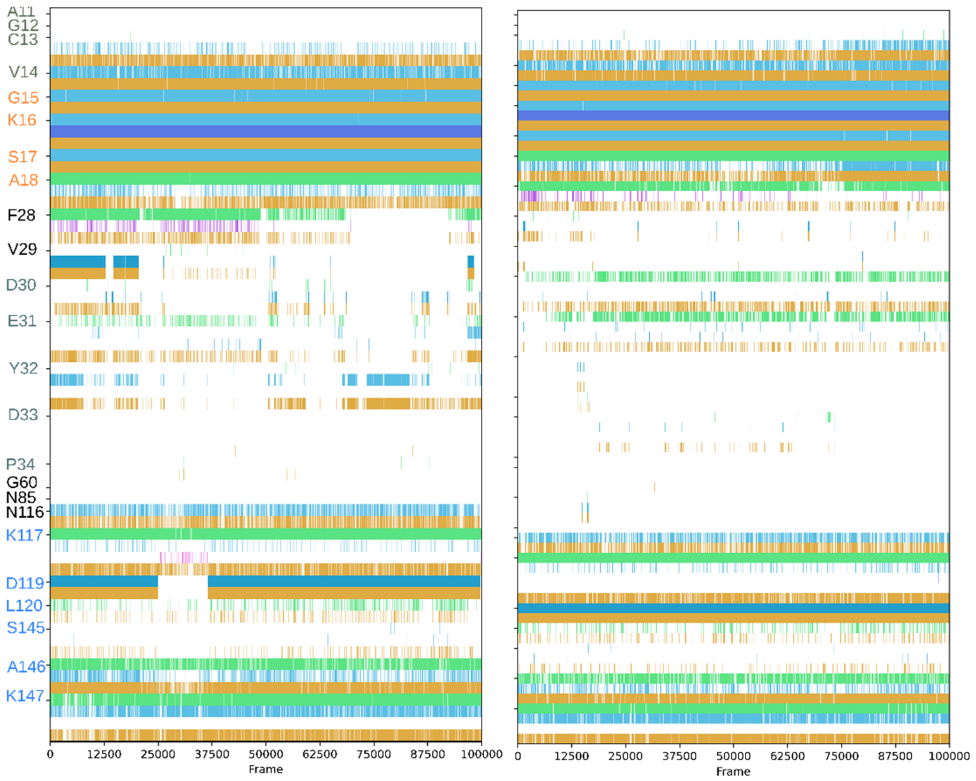

7i

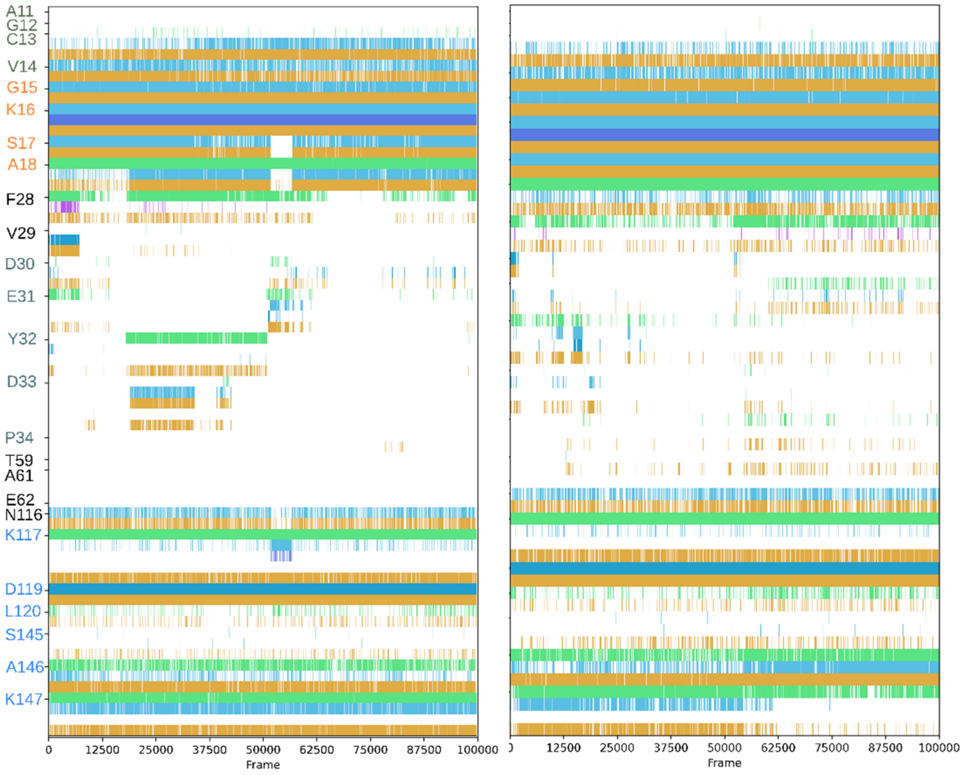

Figure S7: Intermolecular Interaction Profile Between KRAS Protein and Ligands 7a, 7b, 7c, 7d, 7f and 7i for two independent simulations each (left and right). The figure, produced with ProLIF (Protein-Ligand Interaction Fingerprints, https://github.com/cbouy/ProLIF) illustrates the intermolecular interaction profile as a function of

simulation time, indicated as the frames produced during the trajectory (that is, the conformations). The interactions are represented by the following colors: hydrophobic contact (green), van der Waals contact (yellow), hydrogen bond acceptor (light cyan), hydrogen bond donor (dark cyan), anionic interaction (blue), cation-π interaction (pink), and π-stacking interaction (purple). The residues marked in green highlight the P-loop region, those in orange indicate residues interacting with the phosphate group, residues in gray-green correspond to the Switch Loop 1, and those in blue represent residues interacting with GDP.

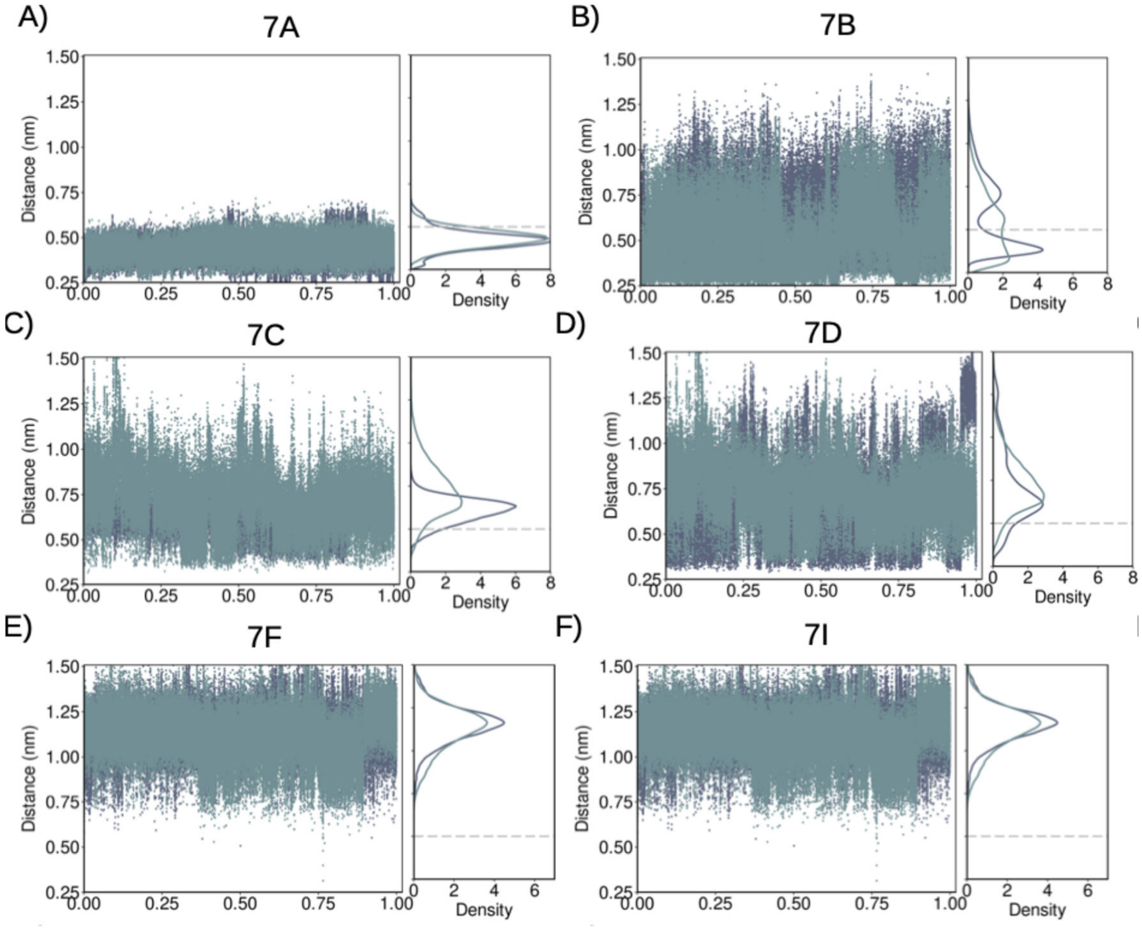

Figure S8: Comparison of two independent simulation replicas for compounds 7A-F. Each panel shows the fluctuation and the distribution of the distance between Cys13 sulfur atom and the oxygen atom from the α-β unsaturated carbonyl group. The graphics demonstrate that both replicas offer equivalent ensembles, demonstrating the statistical robustness and reproducibility of the performed simulations.

LC/MS Spectra – Amine *3b to 3i*

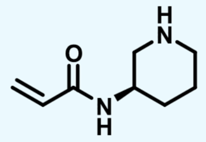

3b

$[M+H]^+$ calculated for $C_8H_{15}N_2O$: 155.1

Retention time: t_R = 1.8 min

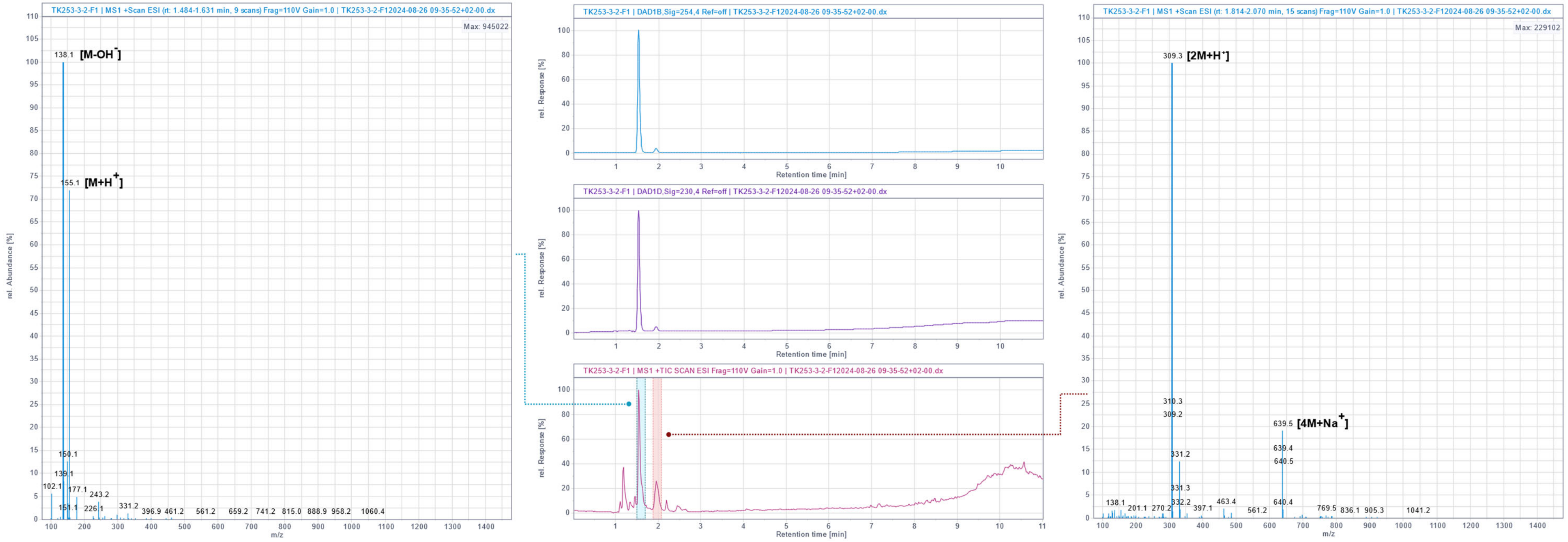

Figure S9: LC/MS Spectra of (*R*)-*N*-(piperidin-3-yl)acrylamide 3b

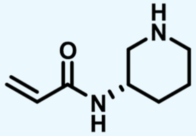

3c

$[M+H]^+$ calculated for $C_8H_{15}N_2O$: 155.1

Retention time: t_R = 1.4 min

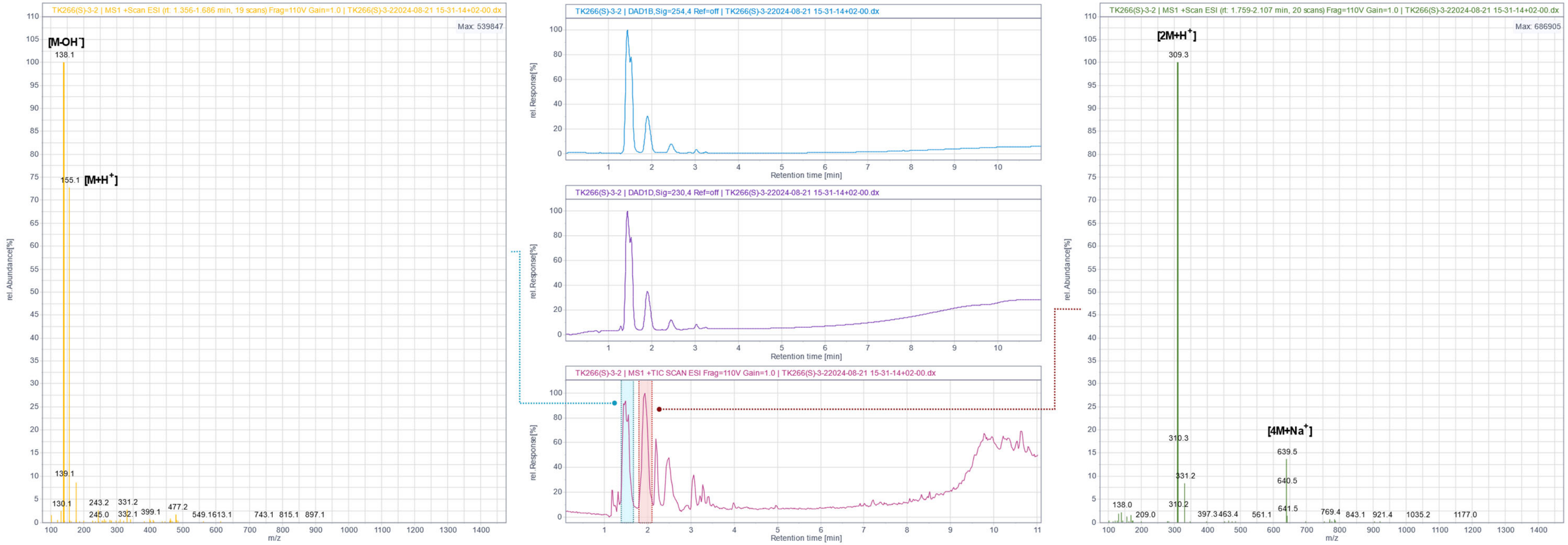

Figure S10: LC/MS Spectra of (*S*)-*N*-(piperidin-3-yl)acrylamide 3c

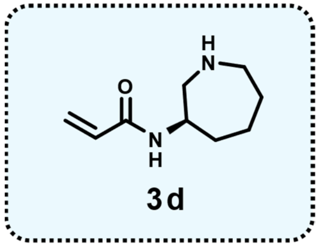

[M+H]$^+$ calculated for $C_9H_{17}N_2O$: 169.1

Retention time: t_R = 2 min

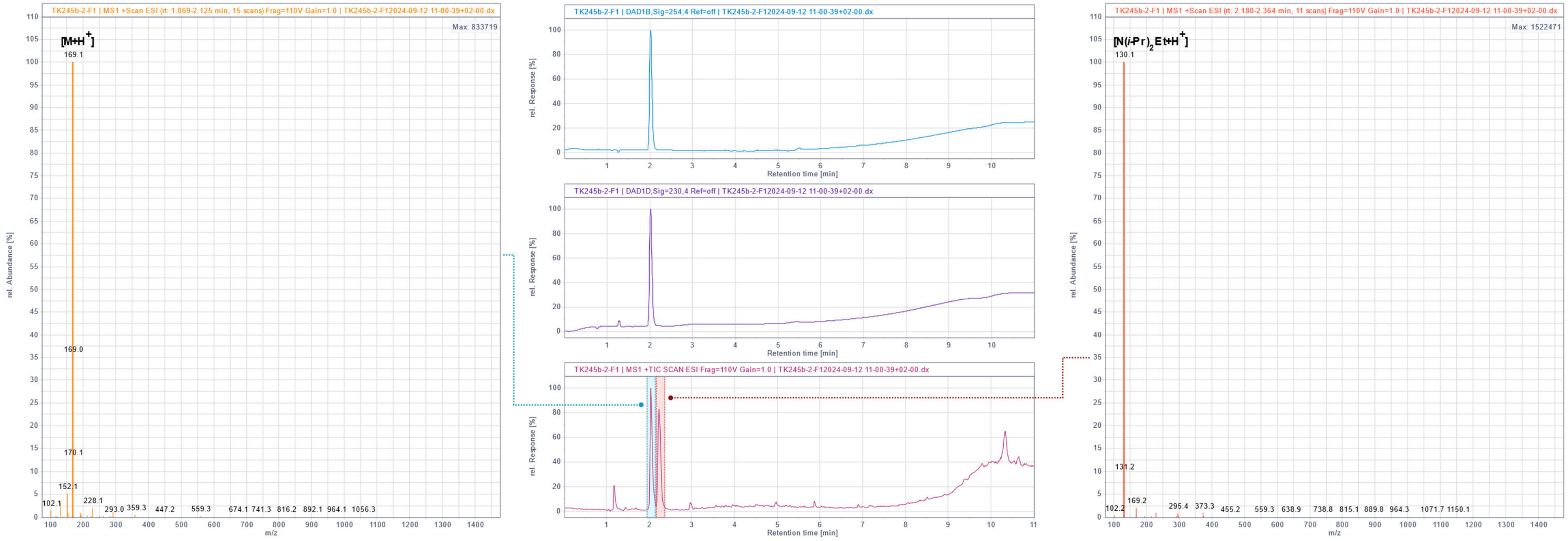

Figure S11: LC/MS Spectra of (*R*)-*N*-(azepan-3-yl)acrylamide 3d

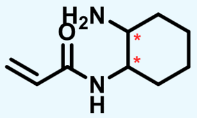

3e

$[M+H]^+$ calculated for $C_9H_{17}N_2O$: 169.1

Retention time: t_R = 4.5 min

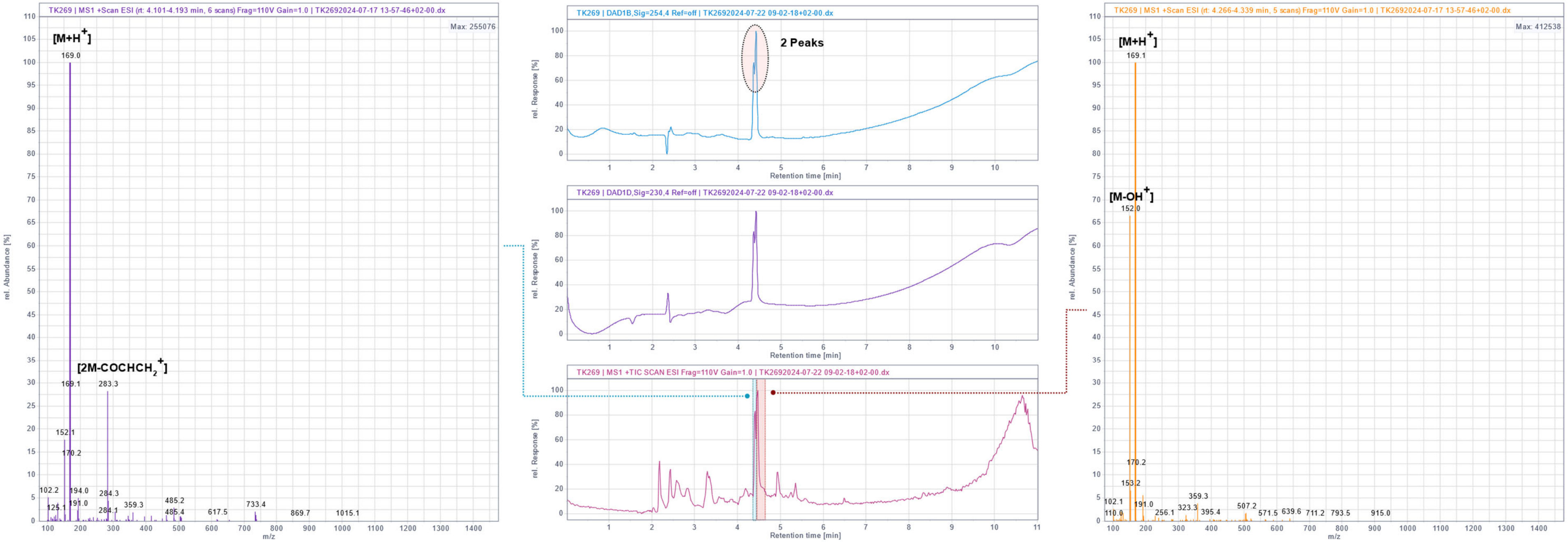

Figure S12: LC/MS Spectra of *N*-(2-aminocyclohexyl)acrylamide 3e

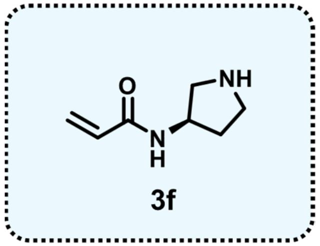

3f

$[M+H]^+$ calculated for $C_7H_{13}N_2O$: 141.1

Retention time: t_R = 1.6 min

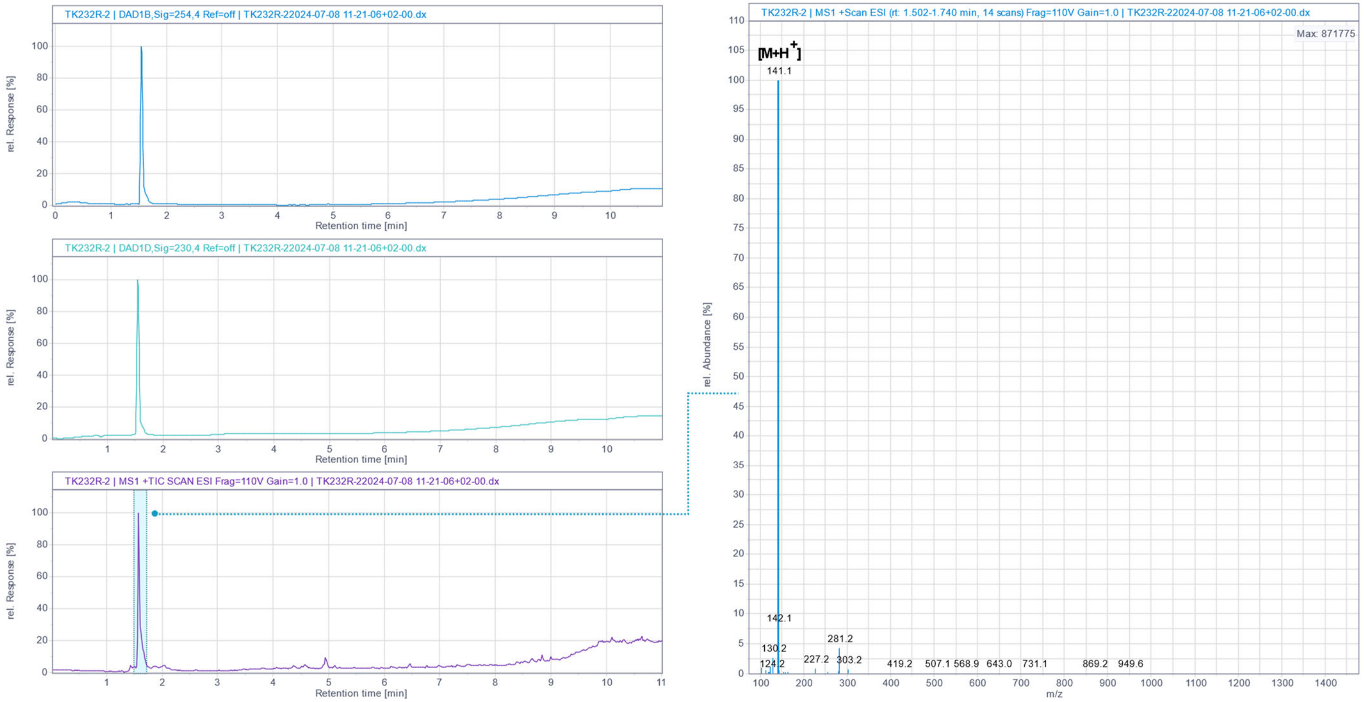

Figure S13: LC/MS Spectra of (*R*)-*N*-(pyrrolidin-3-yl)acrylamide 3f

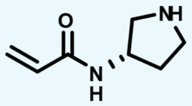

3g

$[M+H]^+$ calculated for $C_7H_{13}N_2O$: 141.1

Retention time: t_R = 1.3 min

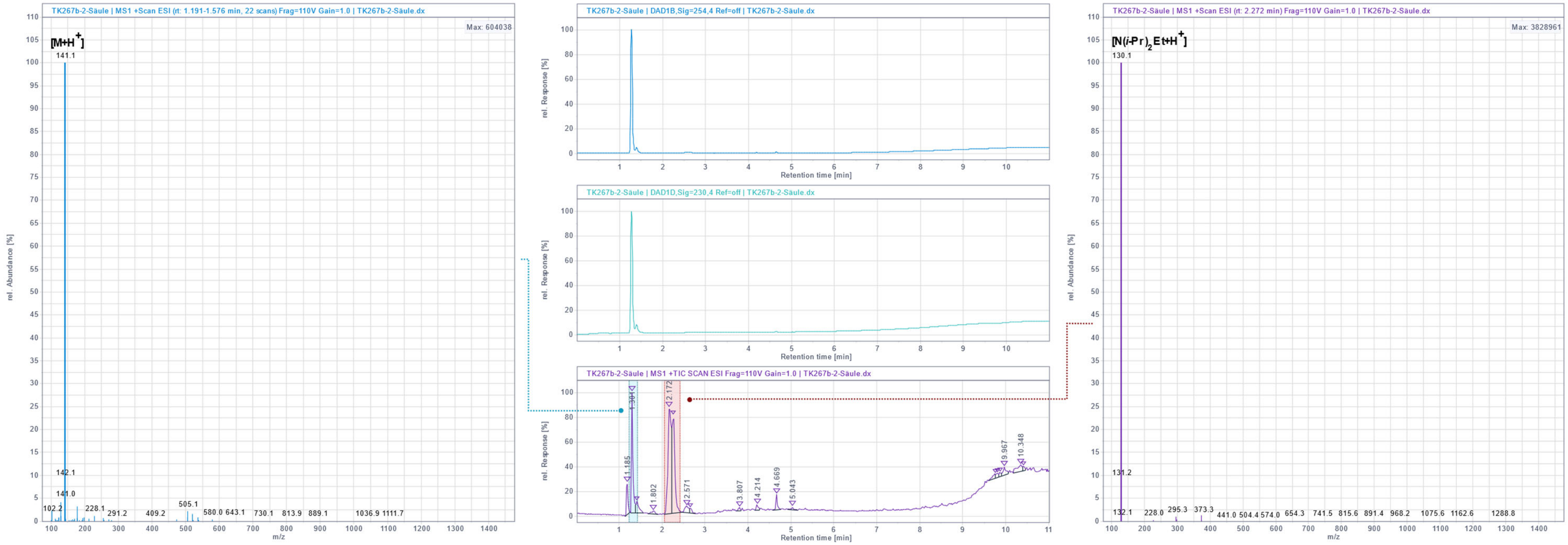

Figure S14: LC/MS Spectra of (*S*)-*N*-(pyrrolidin-3-yl)acrylamide 3g

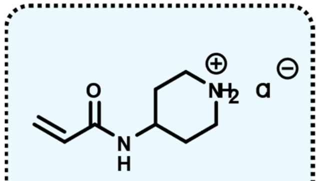

3h

[M+H]+ calculated for $C_8H_{15}N_2O$: 155.1

Retention time: t_R = 1.2 min

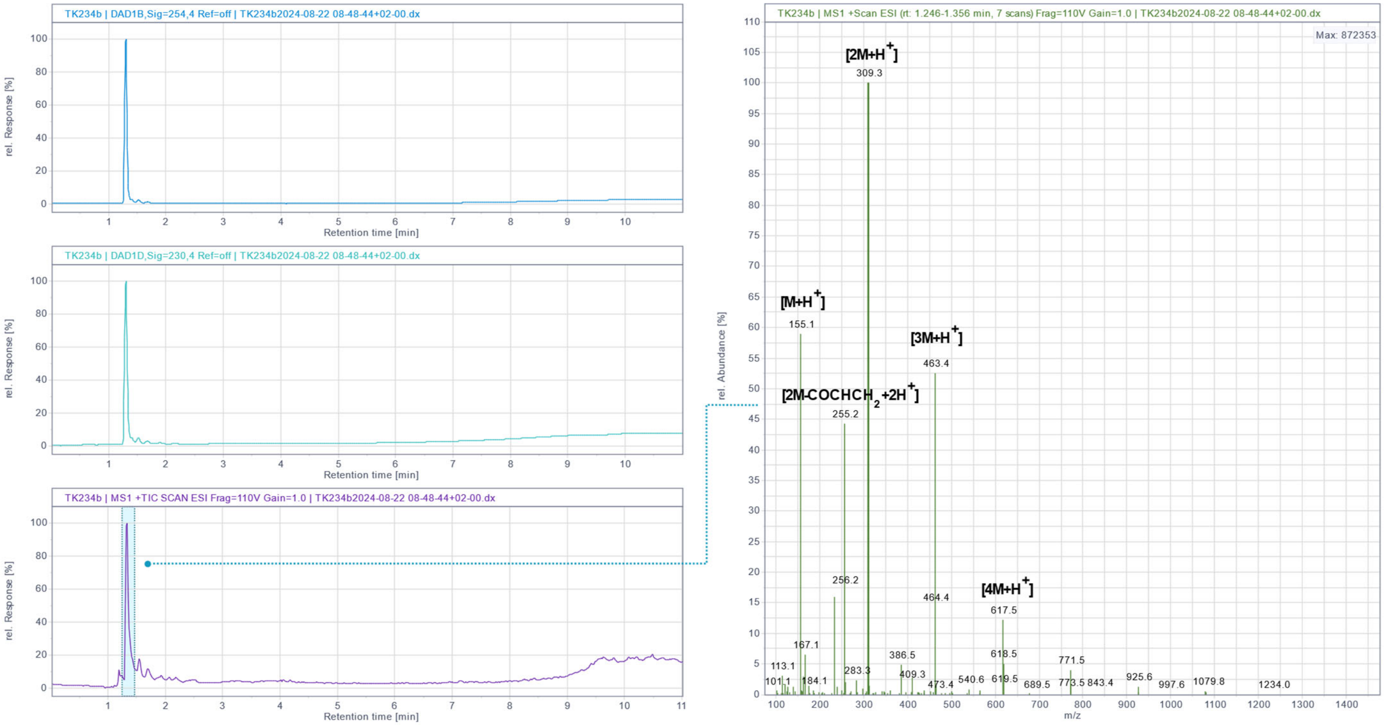

Figure S15: LC/MS Spectra of *N*-(piperidin-4-yl)acrylamide hydrochloride 3h

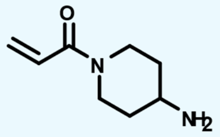

3i

$[M+H]^+$ calculated for $C_8H_{15}N_2O$: 155.1

Retention time: t_R = 1.2 min

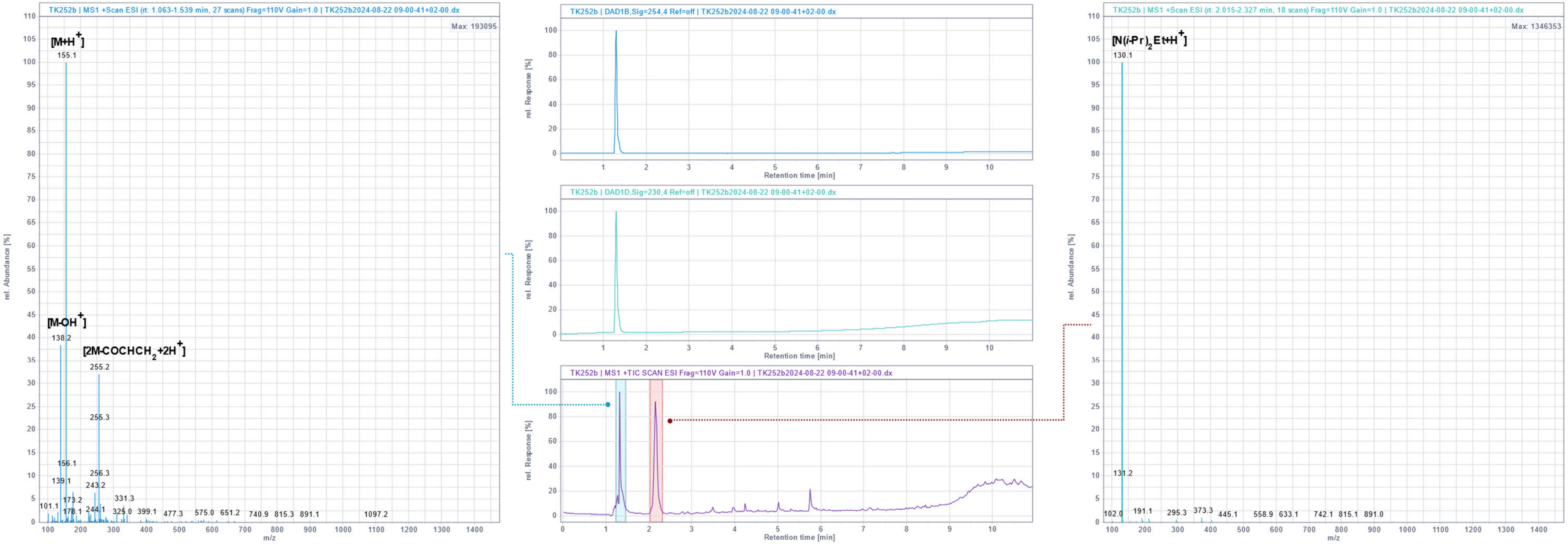

Figure S16: LC/MS Spectra of 1-(4-aminopiperidin-1-yl)prop-2-en-1-one 3i

LC/MS Spectra – GDP *7a to 7i*

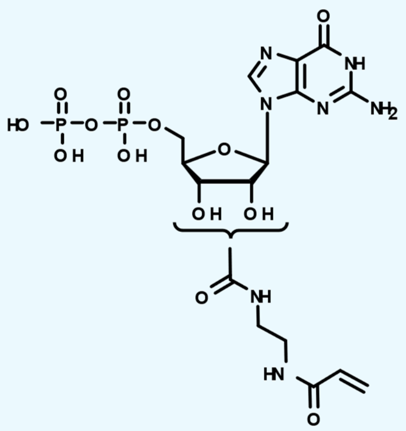

7a

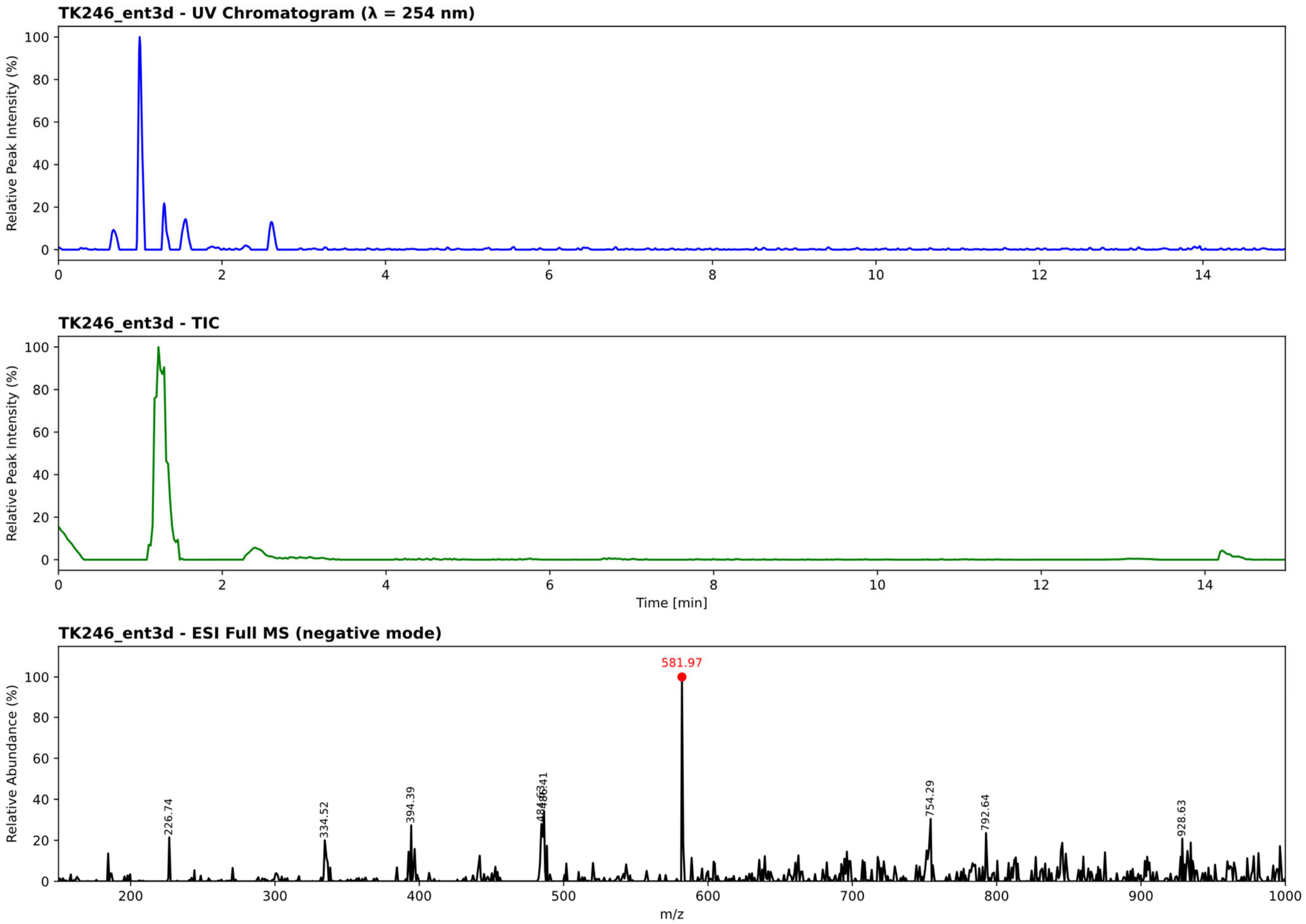

$[M-H]^-$ calculated for $C_{16}H_{22}N_7O_{13}P_2^-$: 581.97

$[M+H]^+$ calculated for $C_{16}H_{24}N_7O_{13}P_2^+$: 583.97

Figure S17: LC/MS Spectra of GDP 7a

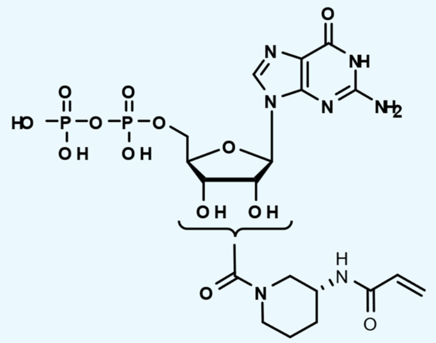

7b

$[M-H]^-$ calculated for $C_{19}H_{26}N_7O_{13}P_2^-$: 622.10

$[M+H]^+$ calculated for $C_{19}H_{28}N_7O_{13}P_2^+$: 624.10

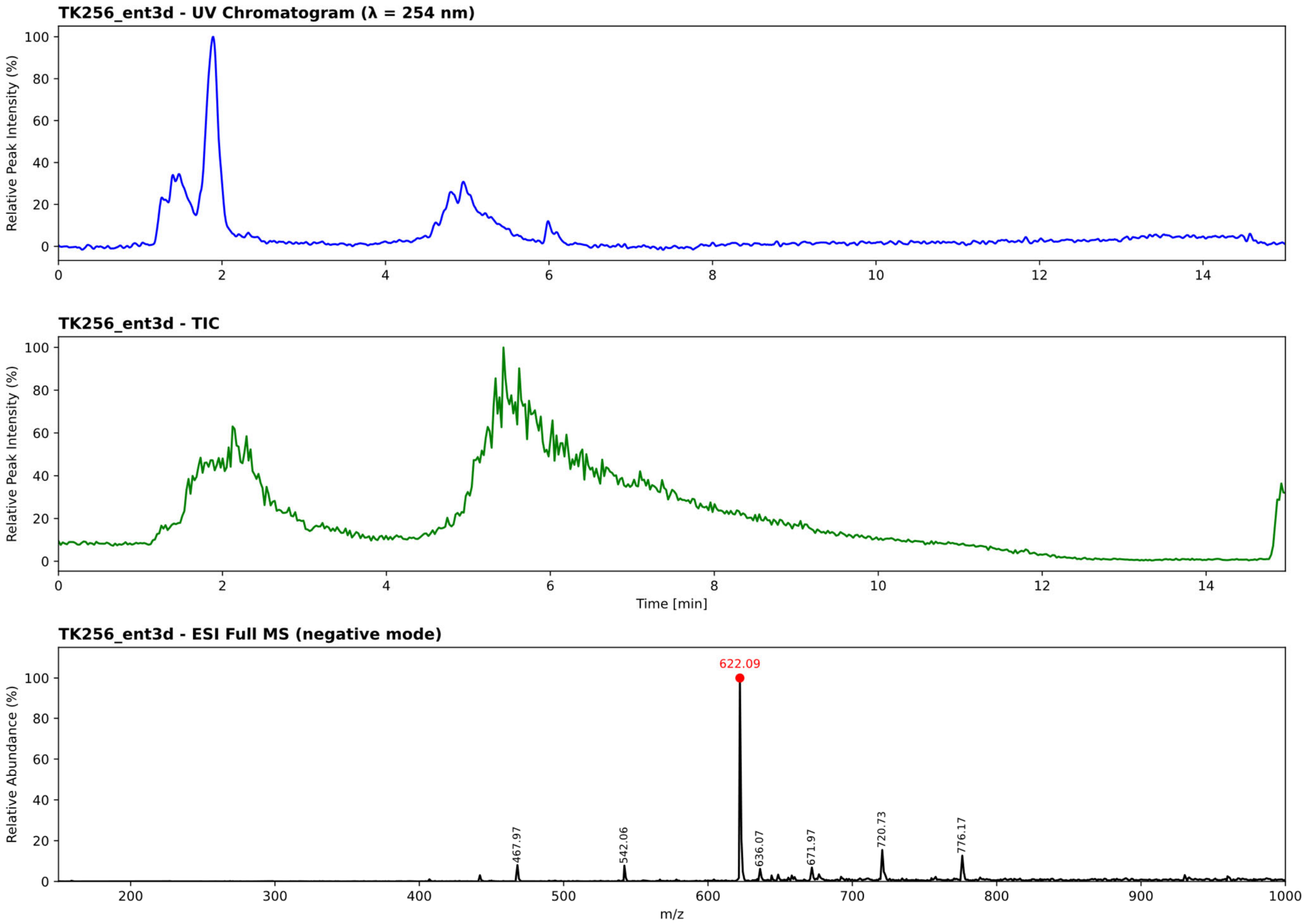

Figure S18: LC/MS Spectra of GDP 7b

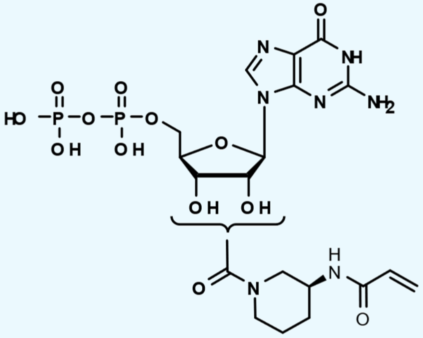

7c

[M-H]$^-$ calculated for $C_{19}H_{26}N_7O_{13}P_2^-$: 622.10

[M+H]$^+$ calculated for $C_{19}H_{28}N_7O_{13}P_2^+$: 624.10

TK268 - UV Chromatograms and TIC

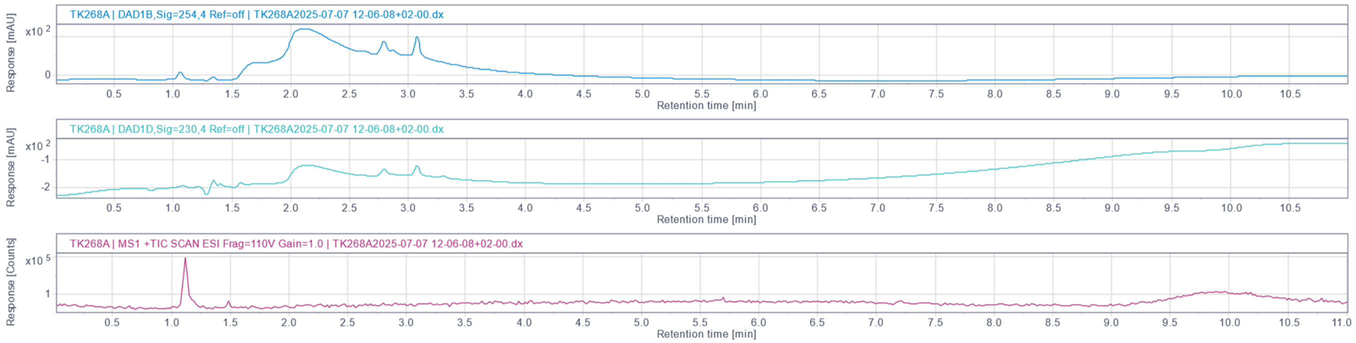

TK268 - Mass spectrum

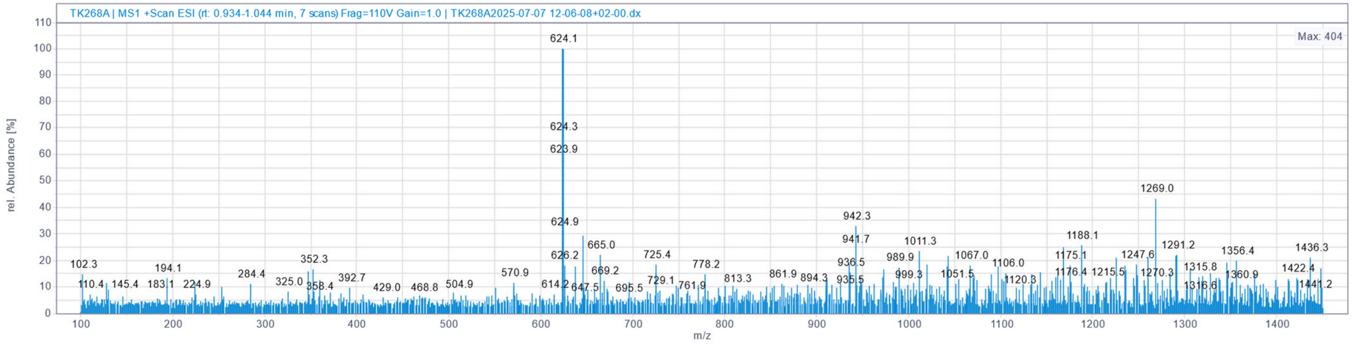

Figure S19: LC/MS Spectra of GDP 7c

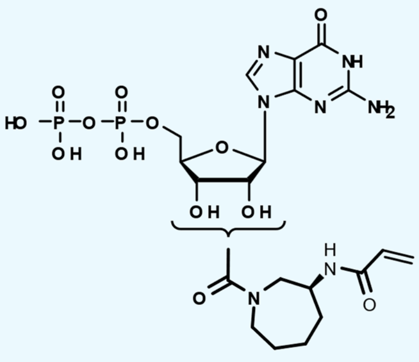

7d

[M-H]⁻ calculated for $C_{20}H_{28}N_7O_{13}P_2^-$: 636.10

[M+H]⁺ calculated for $C_{20}H_{30}N_7O_{13}P_2^+$: 638.10

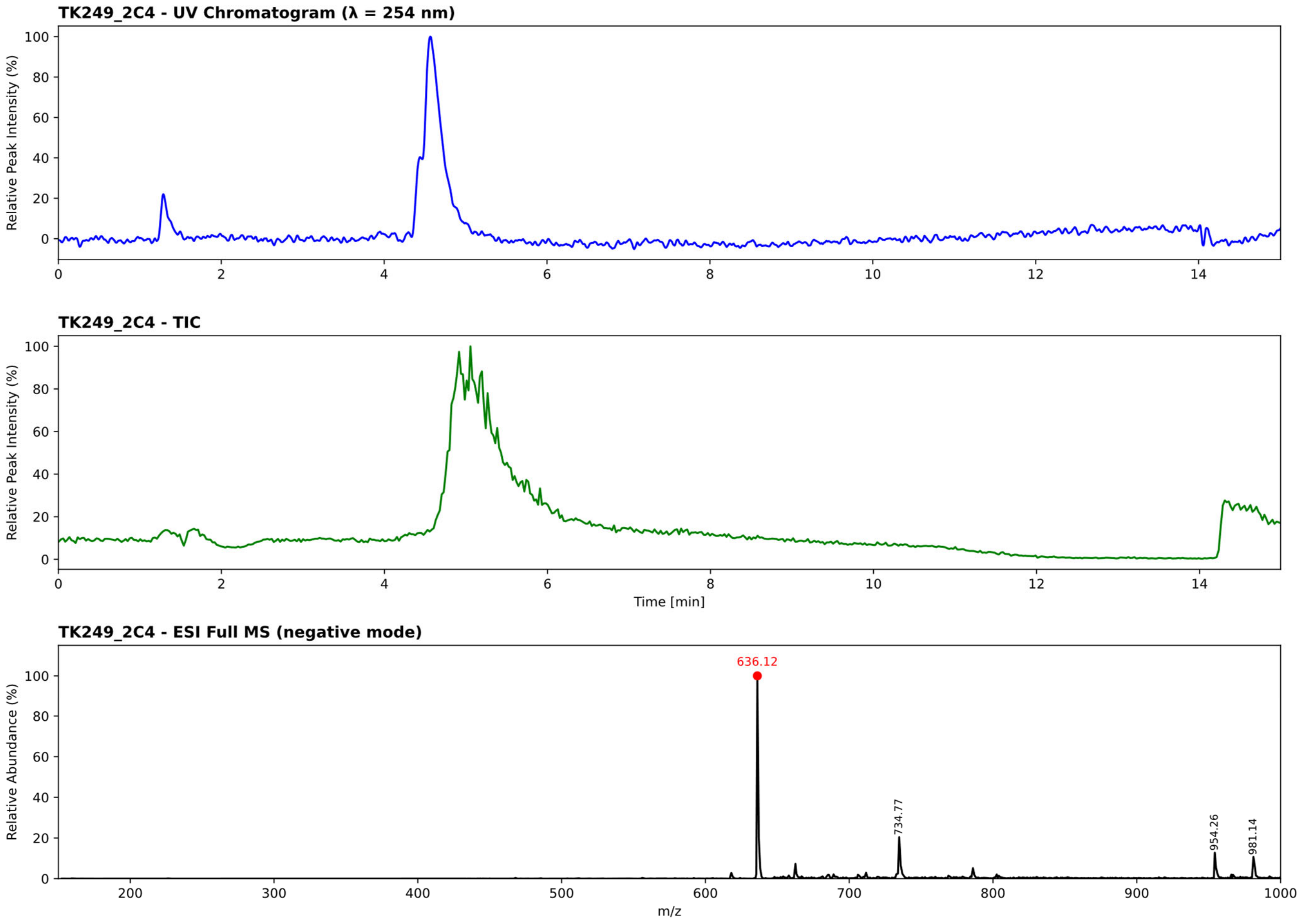

Figure S20: LC/MS Spectra of GDP 7d

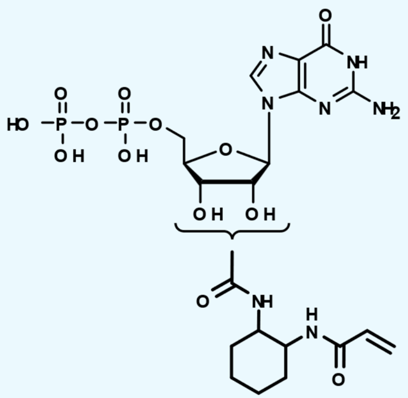

7e

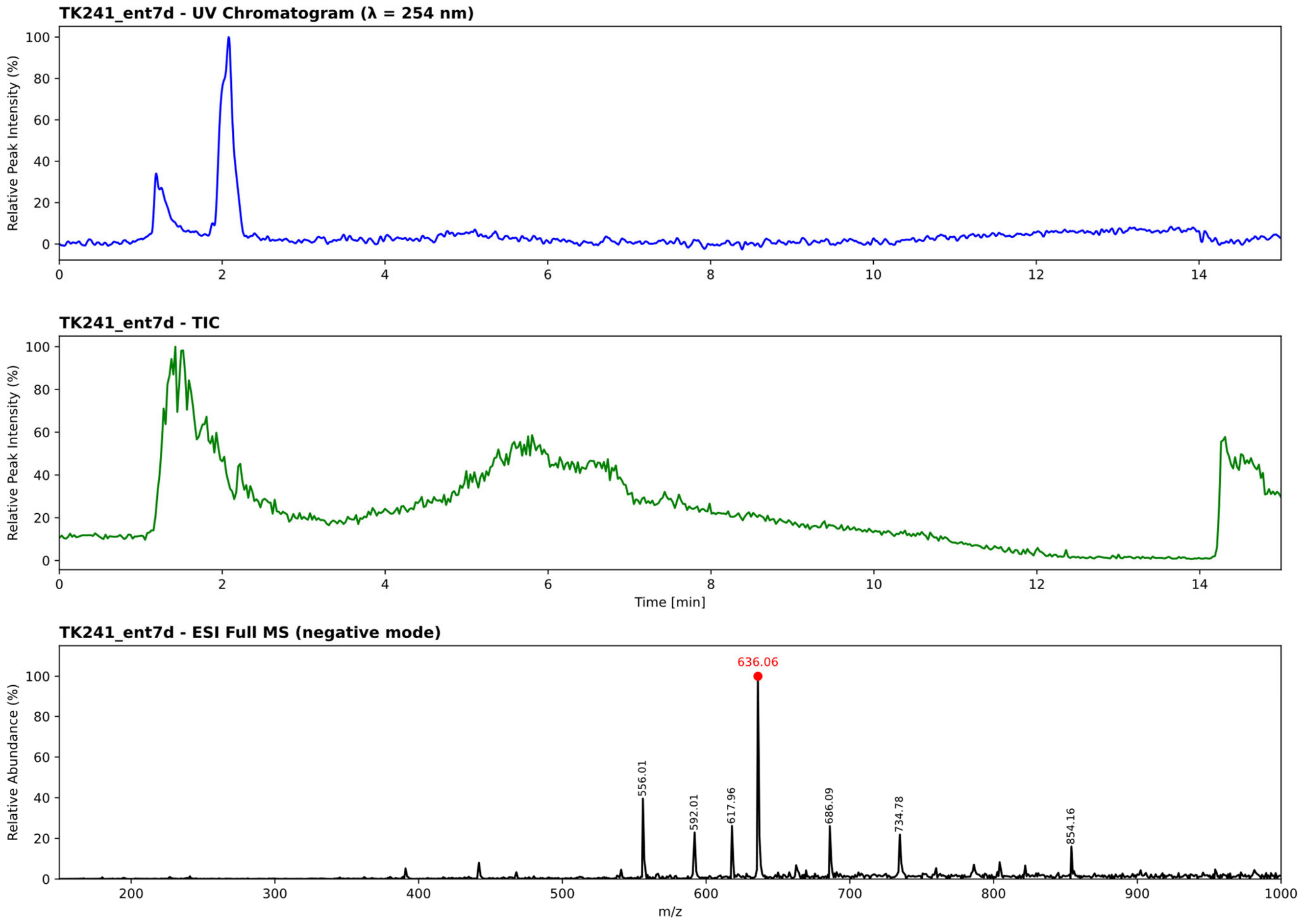

[M-H]$^-$ calculated for $C_{20}H_{28}N_7O_{13}P_2^-$: 636.04

[M+H]$^+$ calculated for $C_{20}H_{30}N_7O_{13}P_2^+$: 638.04

Figure S21: LC/MS Spectra of GDP 7e

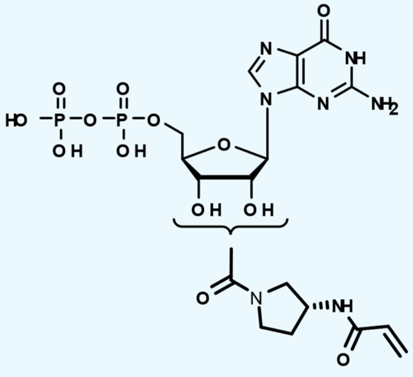

7f

[M-H]$^-$ calculated for $C_{18}H_{24}N_7O_{13}P_2^-$: 608.10

[M+H]$^+$ calculated for $C_{18}H_{26}N_7O_{13}P_2^+$: 610.10

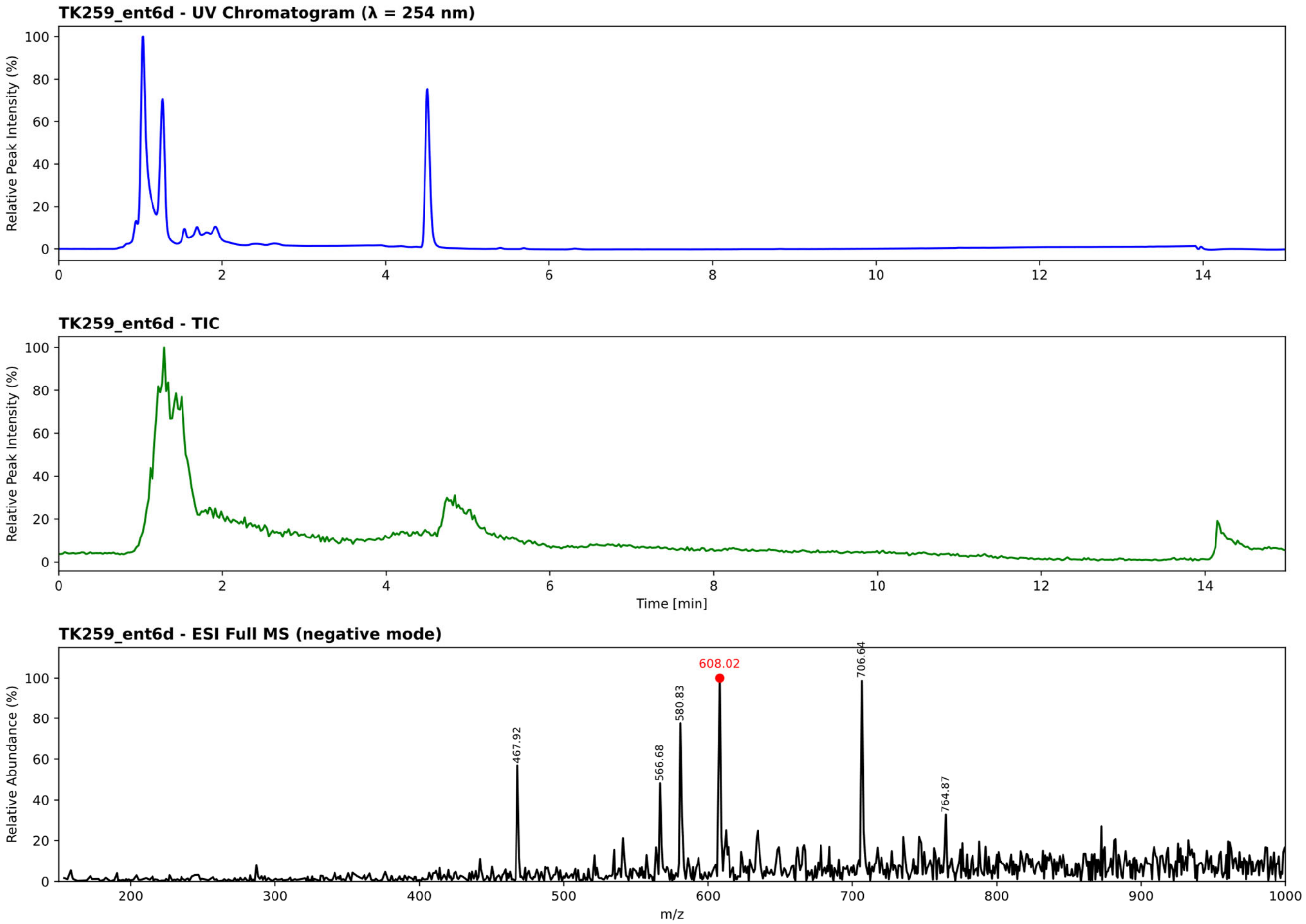

Figure S22: LC/MS Spectra of GDP 7f

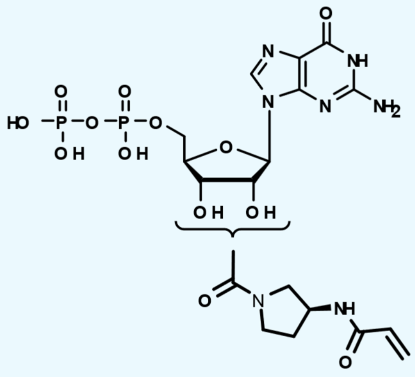

7g

[M-H]$^-$ calculated for $C_{18}H_{24}N_7O_{13}P_2^-$: 608.10

[M+H]$^+$ calculated for $C_{18}H_{26}N_7O_{13}P_2^+$: 610.10

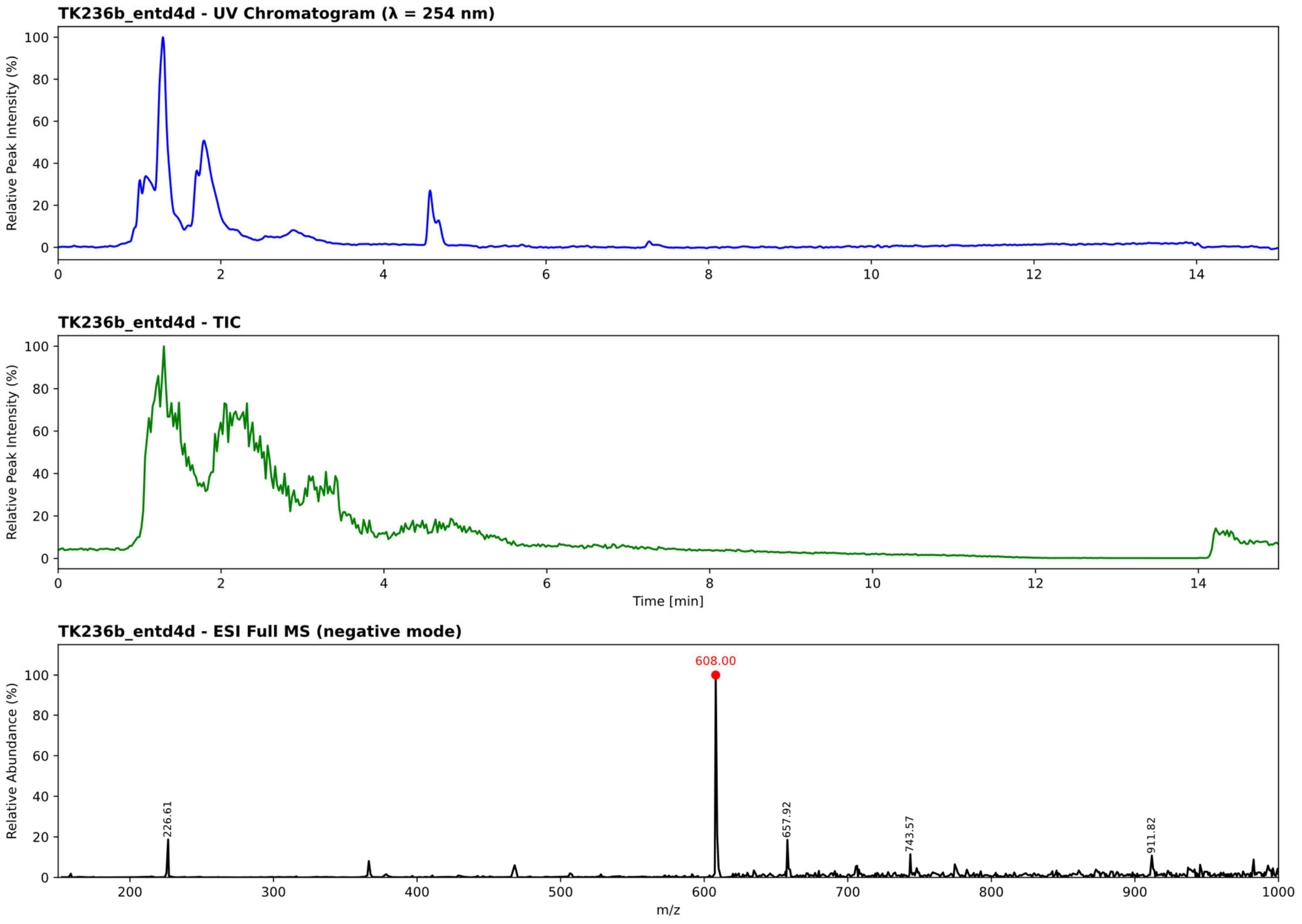

Figure S23: LC/MS Spectra of GDP 7g

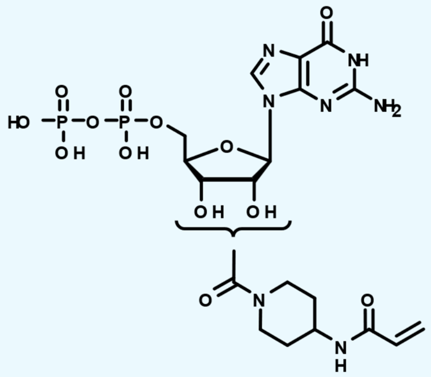

7h

[M-H]$^-$ calculated for $C_{19}H_{26}N_7O_{13}P_2^-$: 622.11

[M+H]$^+$ calculated for $C_{19}H_{28}N_7O_{13}P_2^+$: 624.11

TK242 - UV Chromatograms and TIC

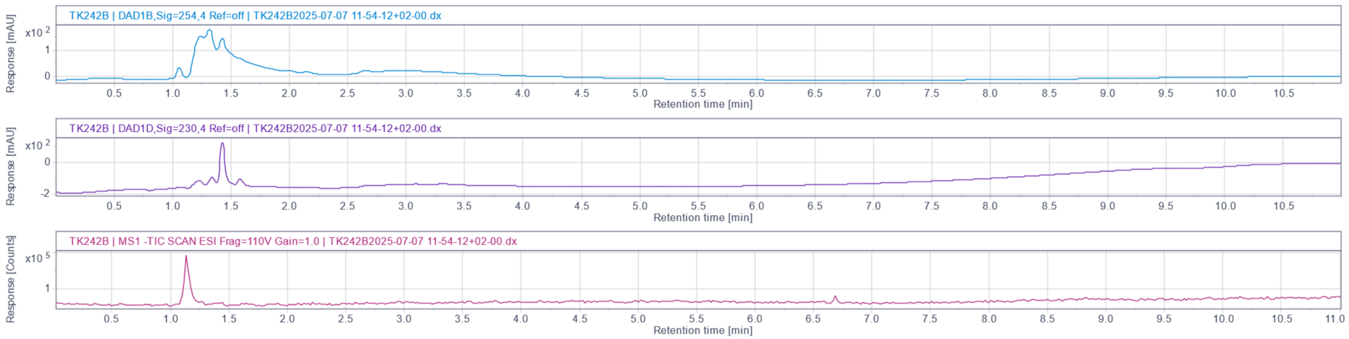

TK242 - Mass spectrum

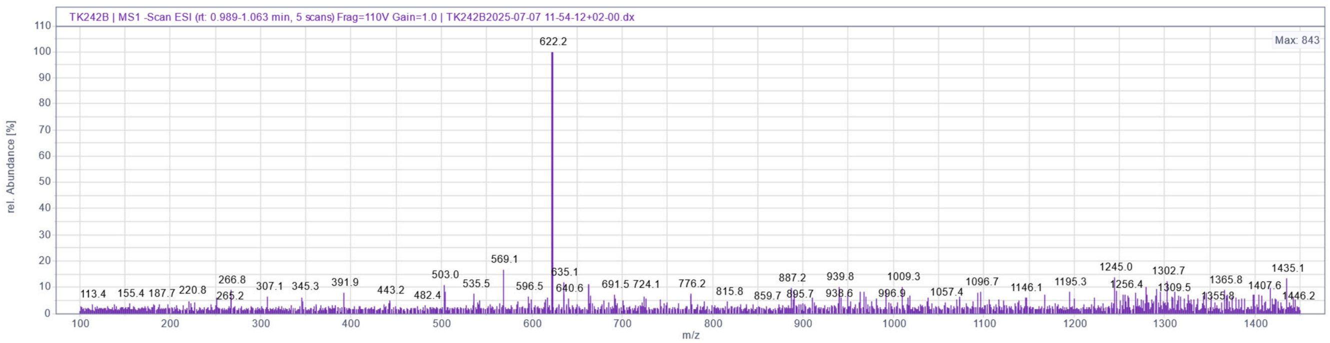

Figure S24: LC/MS Spectra of GDP 7h

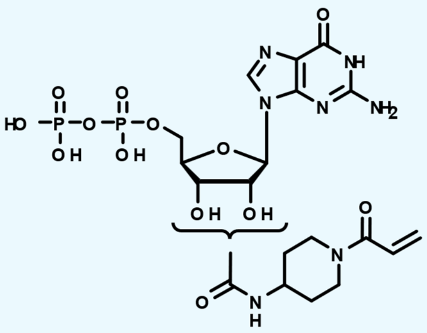

7i

[M-H]$^-$ calculated for $C_{19}H_{26}N_7O_{13}P_2^-$: 622.11

[M+H]$^+$ calculated for $C_{19}H_{28}N_7O_{13}P_2^+$: 624.11

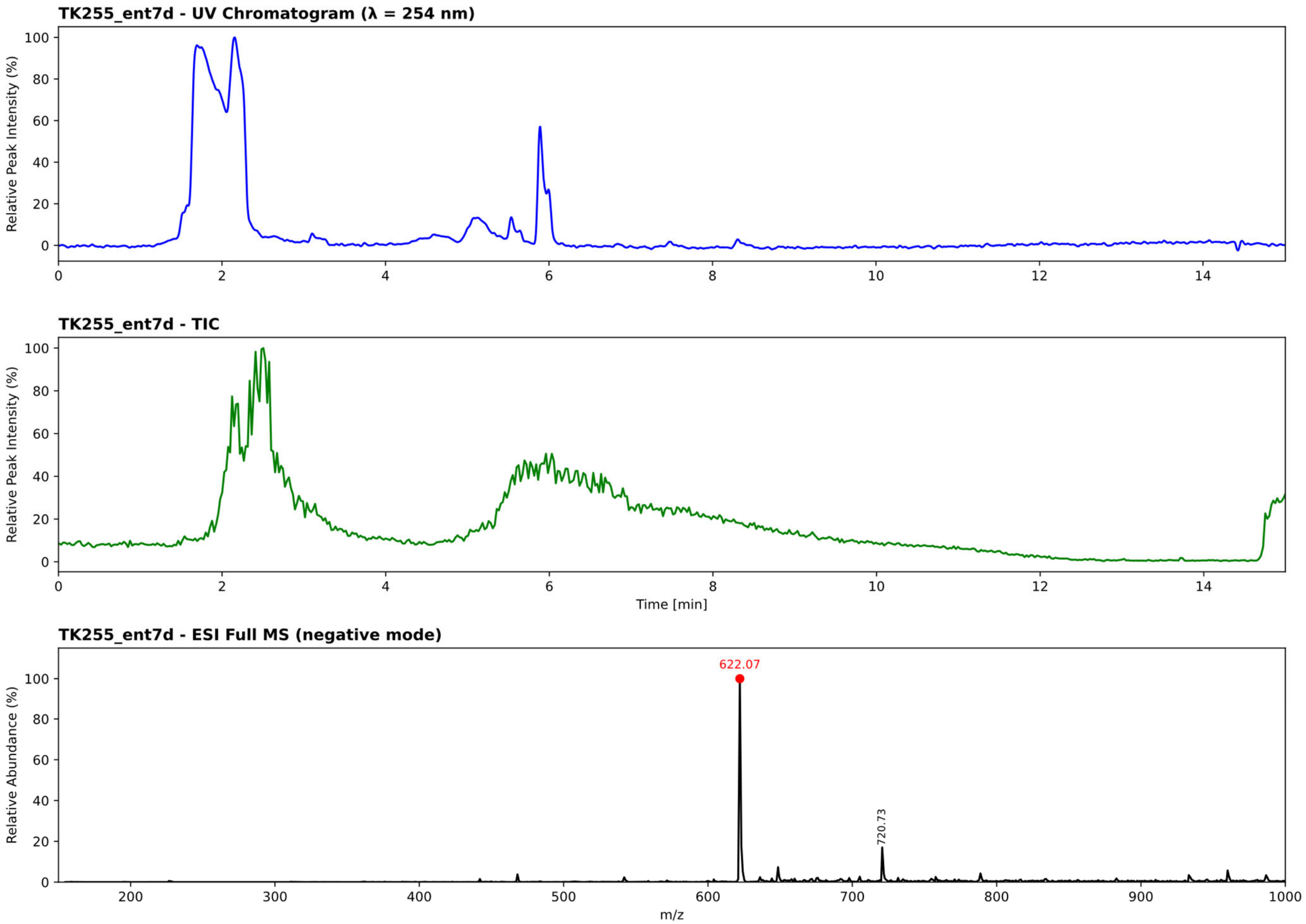

Figure S25: LC/MS Spectra of GDP 7i

HRMS Spectra – GDP *7b to 7e*

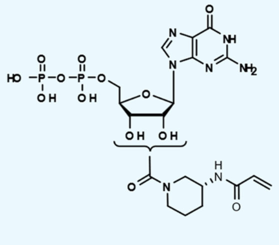

7b

[M-H]$^-$ calculated for $C_{19}H_{26}N_7O_{13}P_2^-$: 622.10

[M+H]$^+$ calculated for $C_{19}H_{28}N_7O_{13}P_2^+$: 624.10

TIC:

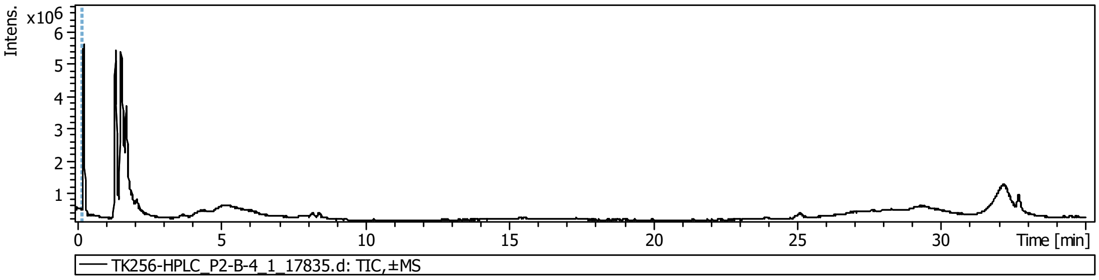

EIC:

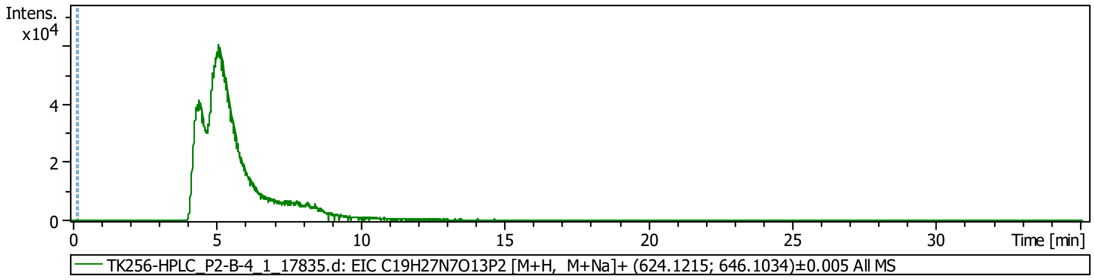

UV-Chromatogram:

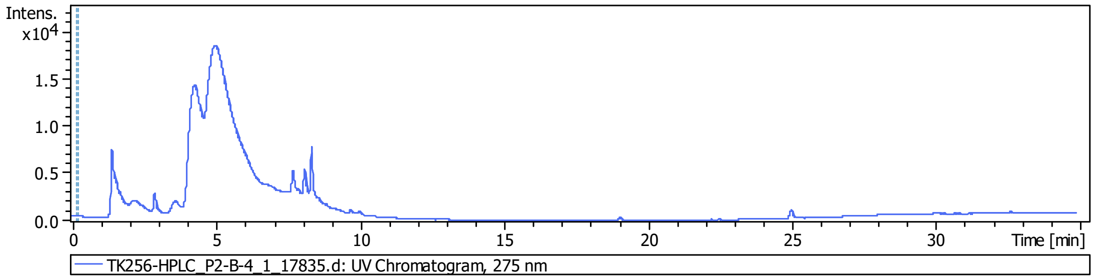

EIC und UV:

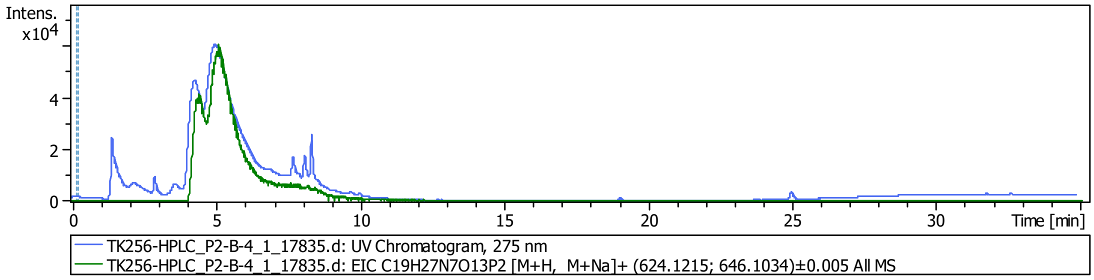

Spectra of peaks:

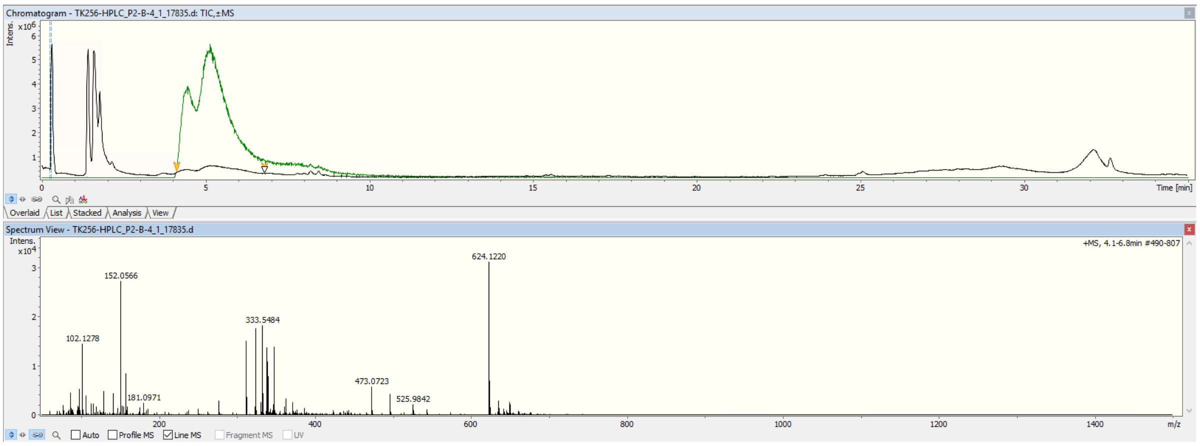

Mass spectrum

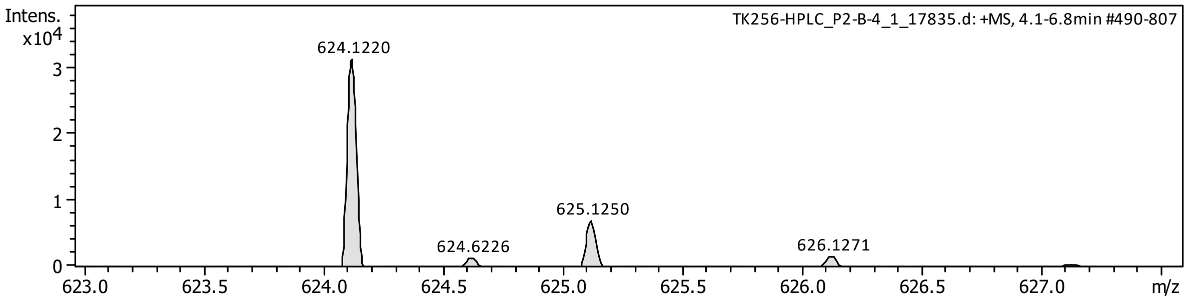

Figure S26: HRMS Spectra of GDP 7b. $[M+H]^+$ with -0,9 ppm deviation; Overlap with another mass.

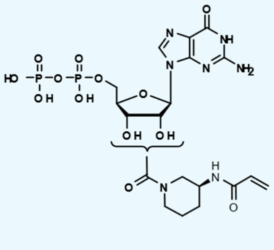

[M-H]$^-$ calculated for $C_{19}H_{26}N_7O_{13}P_2^-$: 622.10

[M+H]$^+$ calculated for $C_{19}H_{28}N_7O_{13}P_2^+$: 624.10

TIC:

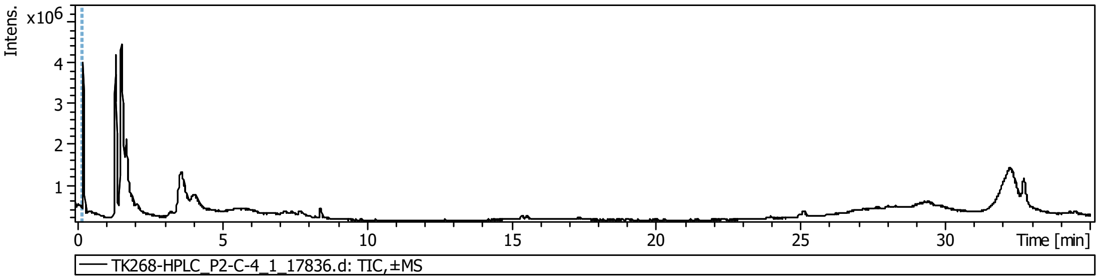

EIC:

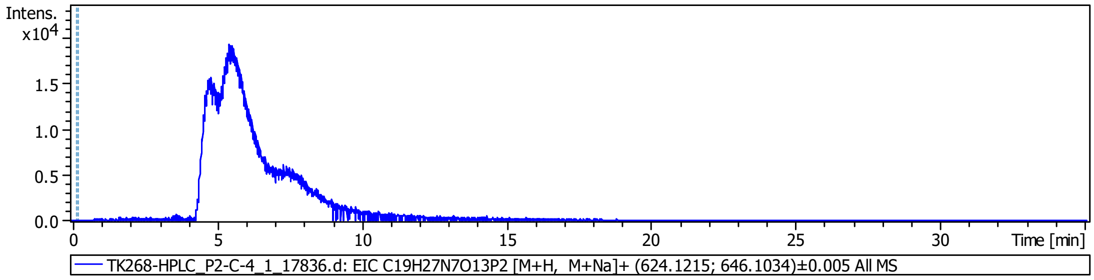

UV-Chromatogram:

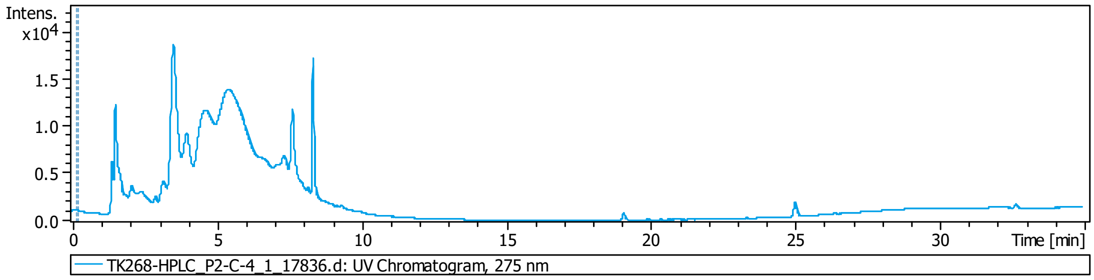

EIC und UV:

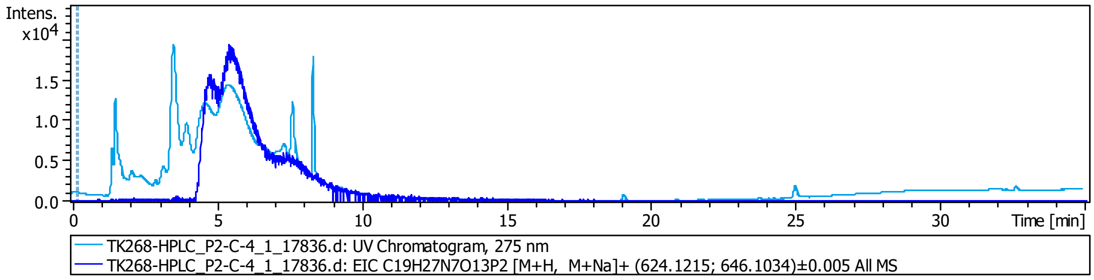

Spectra of peaks:

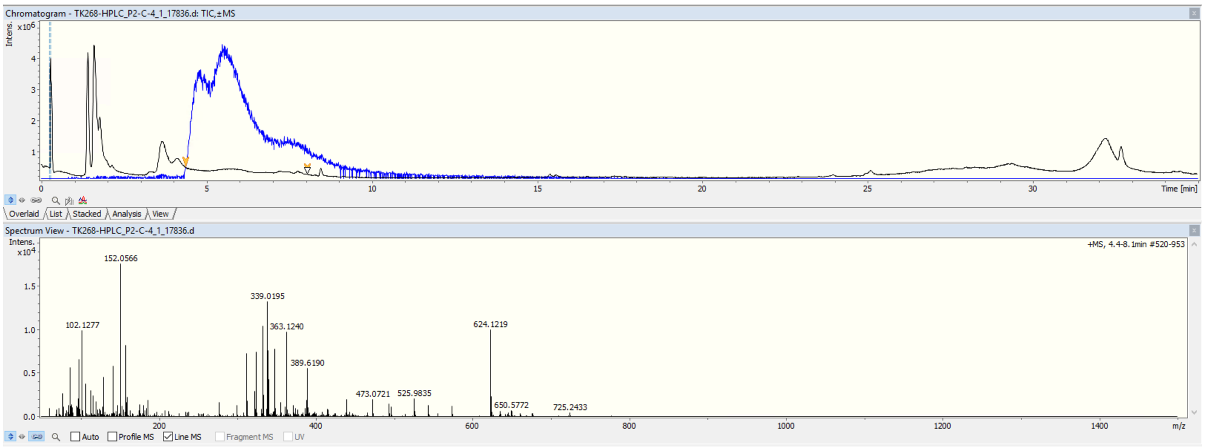

Mass spectrum

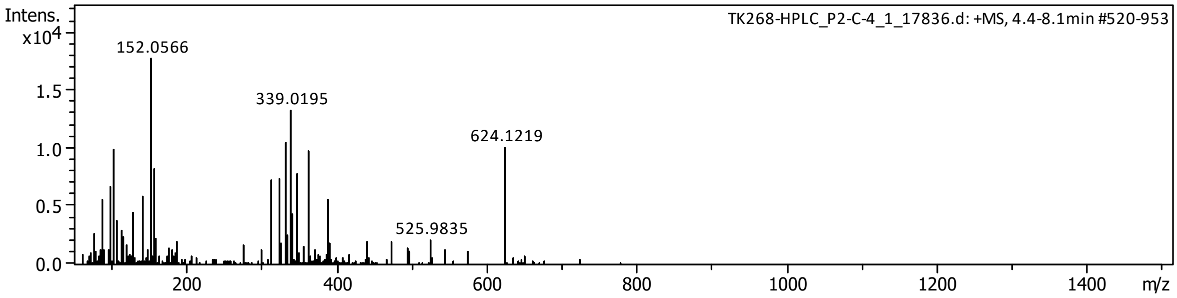

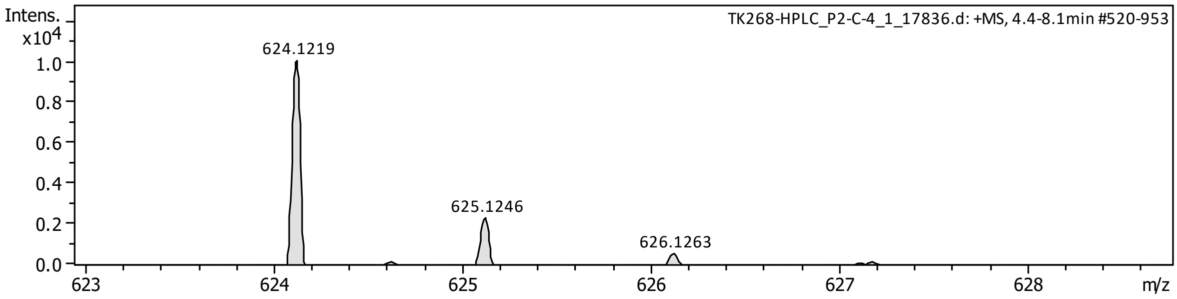

Figure S27: HRMS Spectra of GDP 7c. $[M+H]^+$ with -0,6 ppm deviation.

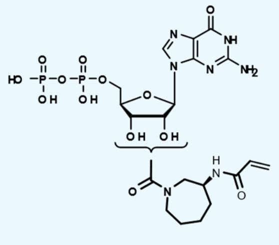

[M-H]$^-$ calculated for $C_{20}H_{28}N_7O_{13}P_2^-$: 636.10

[M+H]$^+$ calculated for $C_{20}H_{30}N_7O_{13}P_2^+$: 638.10

TIC:

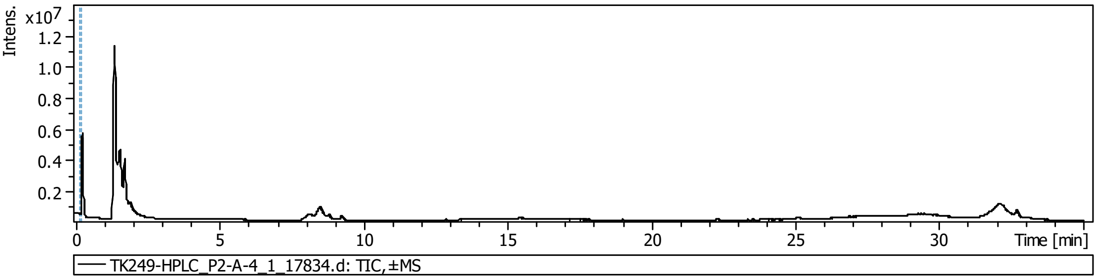

EIC:

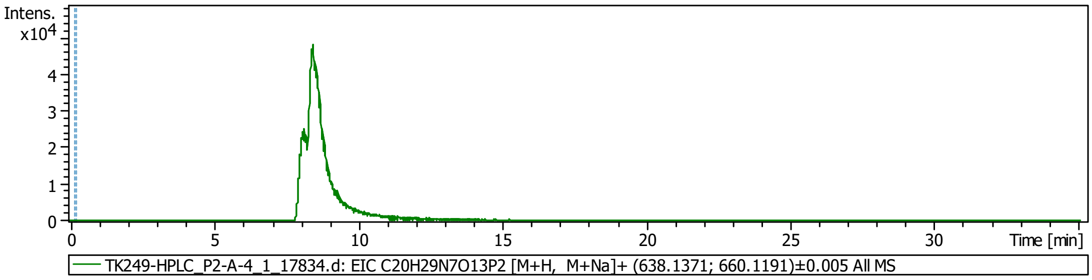

UV-Chromatogram:

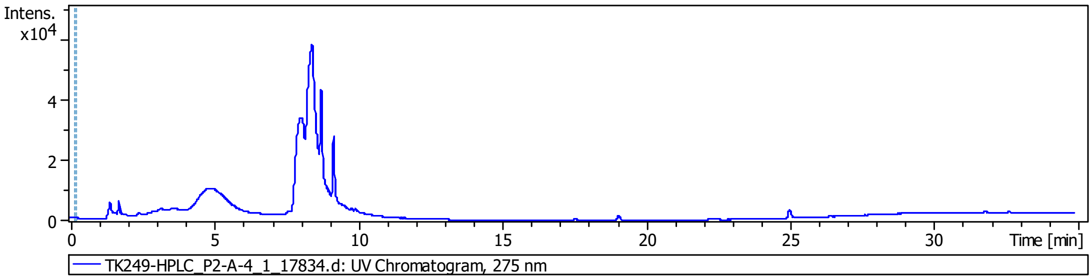

EIC und UV:

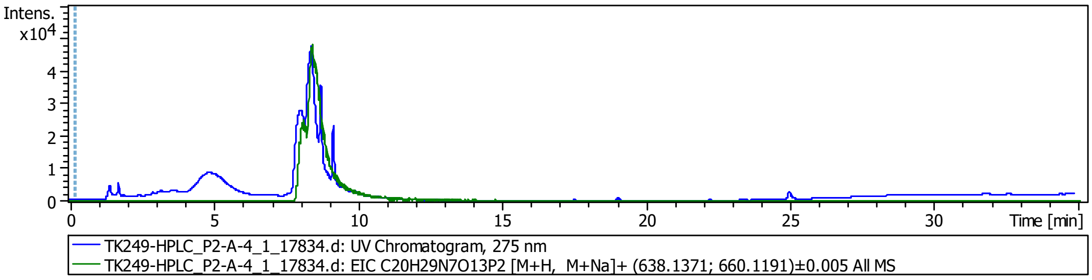

Spectra of peaks:

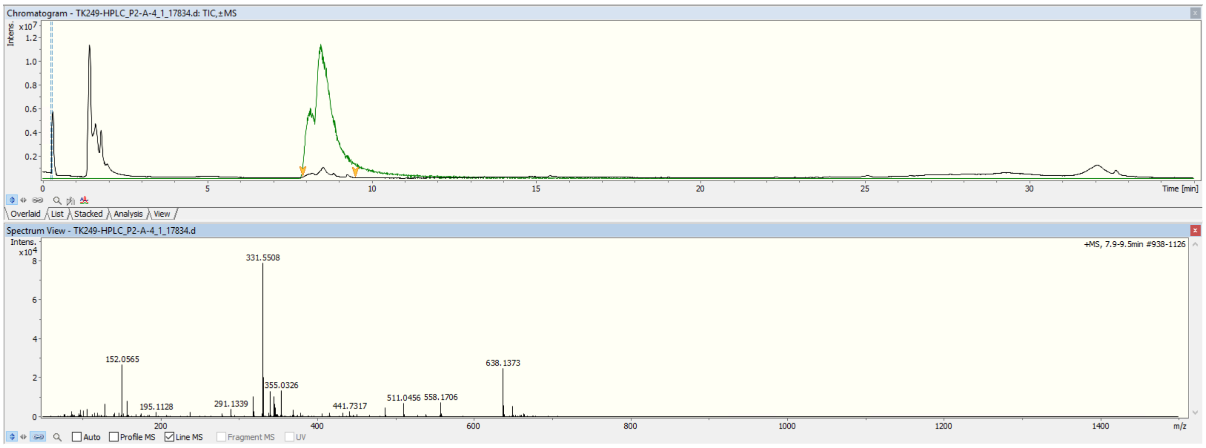

Mass spectrum

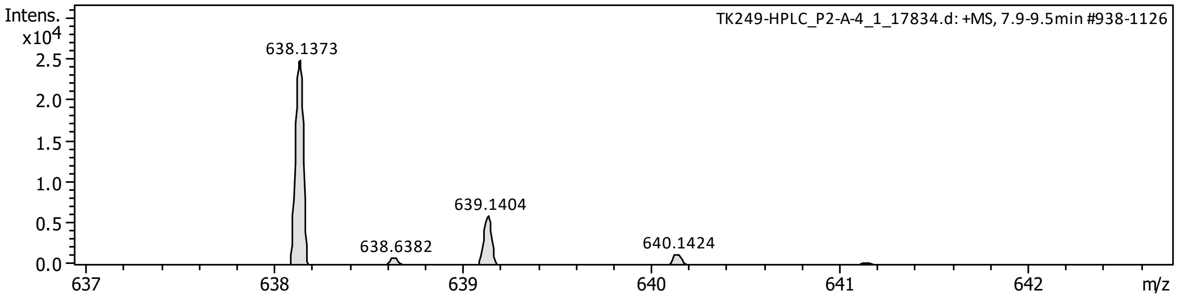

Figure S28: HRMS Spectra of GDP 7d. $[M+H]^+$ with -0,2 ppm deviation; Overlayed with another mass.

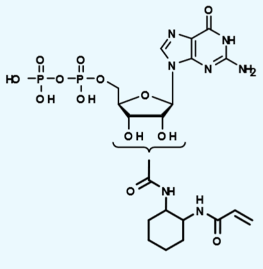

7e

[M-H]$^-$ calculated for $C_{20}H_{28}N_7O_{13}P_2^-$: 636.04

[M+H]$^+$ calculated for $C_{20}H_{30}N_7O_{13}P_2^+$: 638.04

TIC:

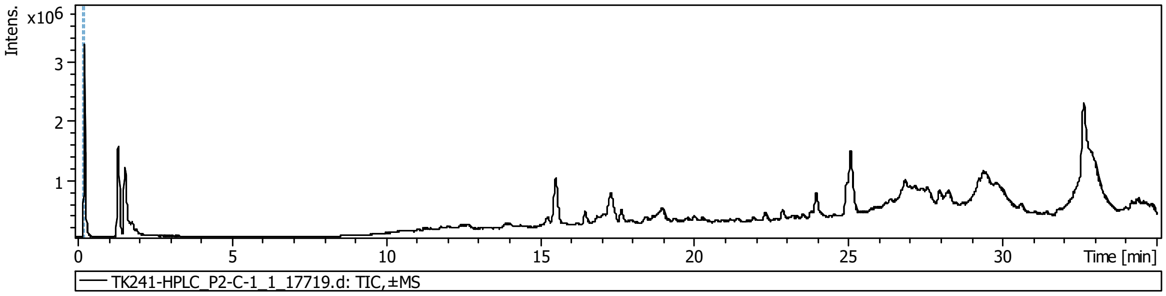

EIC:

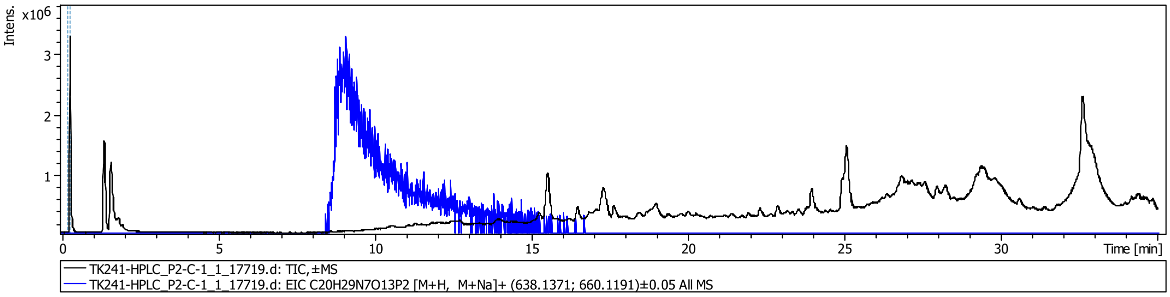

UV-Chromatogram:

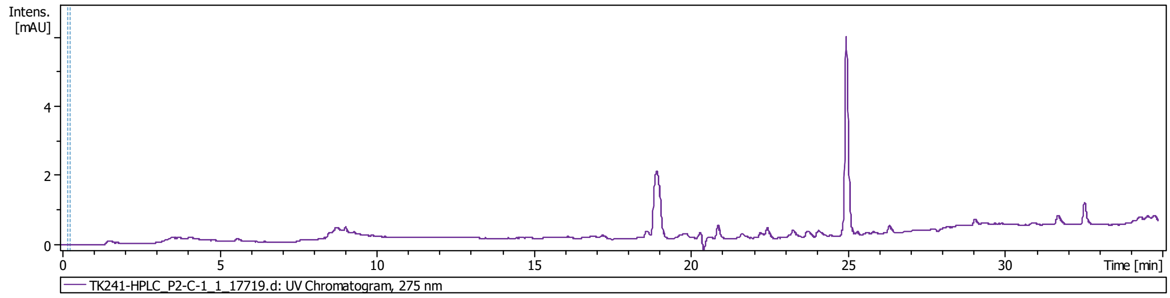

EIC und UV:

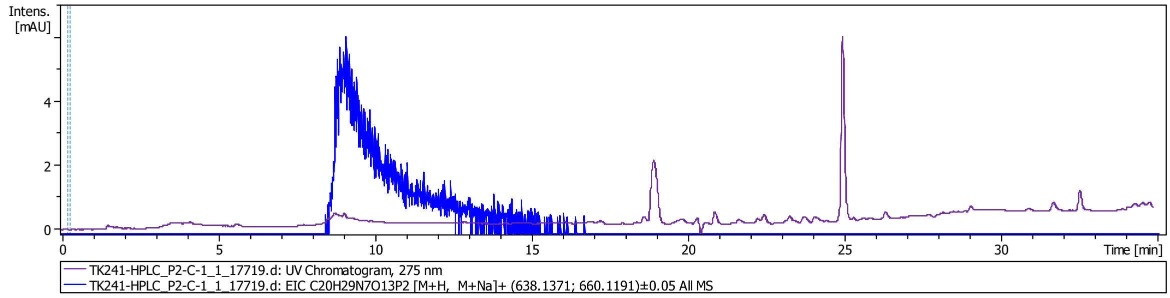

Spectra of peaks:

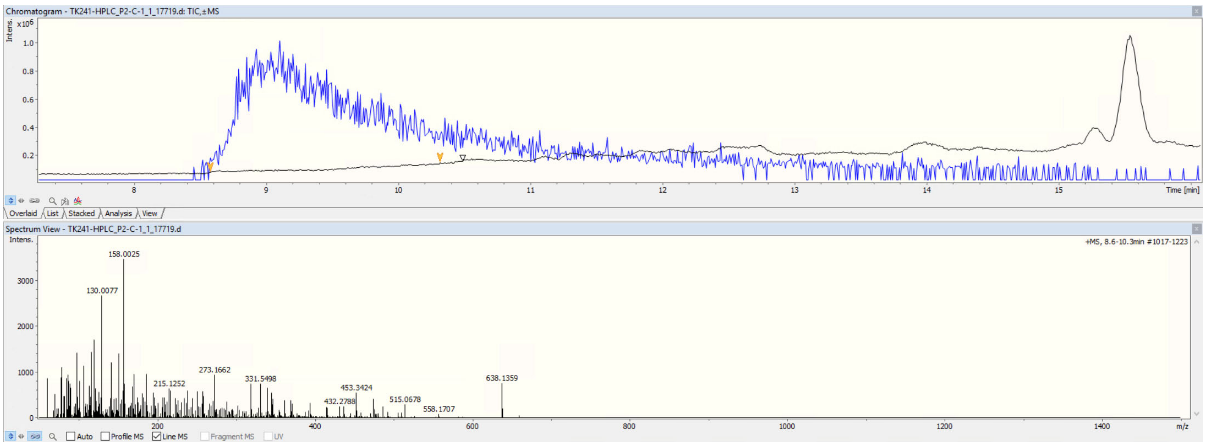

Mass Spectrum

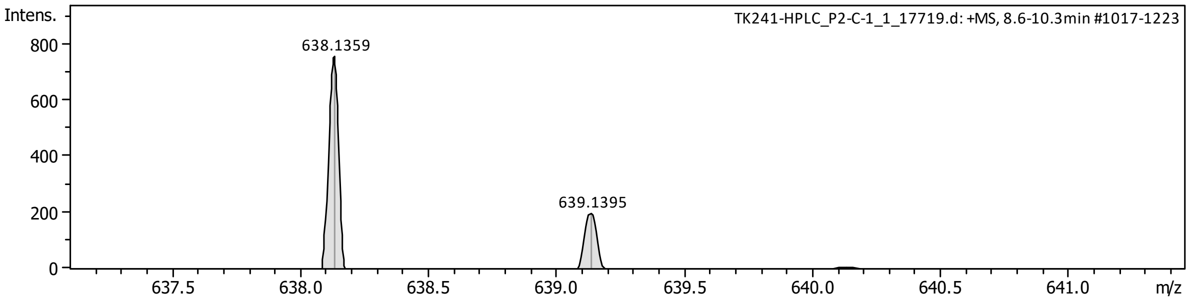

Figure S29: HRMS Spectra of GDP 7e. $[M+H]^+$ with 1,9 ppm deviation.

NMR spectra – Amine *3a to 3i*

Figure S30: ^{1}H NMR Spectrum of (*R*)-*N*-(piperidin-3-yl)acrylamide 3b

^{1}H NMR (400 MHz) in CD_3OD

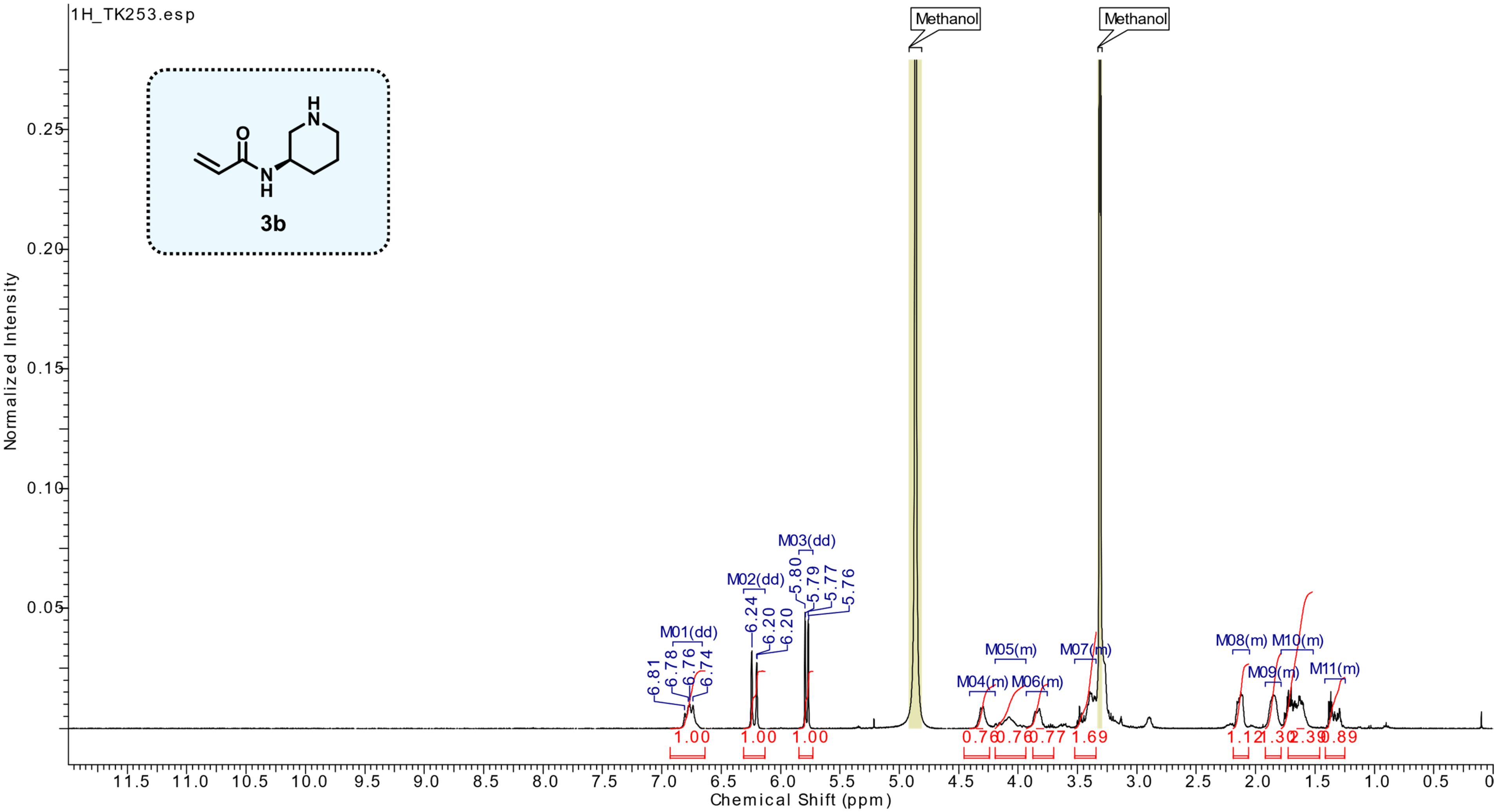

Figure S31: ^{13}C NMR Spectrum of (*R*)-*N*-(piperidin-3-yl)acrylamide 3b

*Due to the low concentration of the NMR sample, the signal-to-noise ratio was insufficient to obtain a reliable ^{13}C NMR spectrum of amine **3b**. Therefore, only the ^{13}C NMR spectrum of the Boc-protected precursor is shown. However, the ^{1}H NMR signals of amine **3b** are consistent with those of the corresponding (S)-enantiomer of amine **3c**. It can therefore be concluded with high confidence that the desired product was obtained.*

^{13}C NMR (175 MHz) in CD_3OD

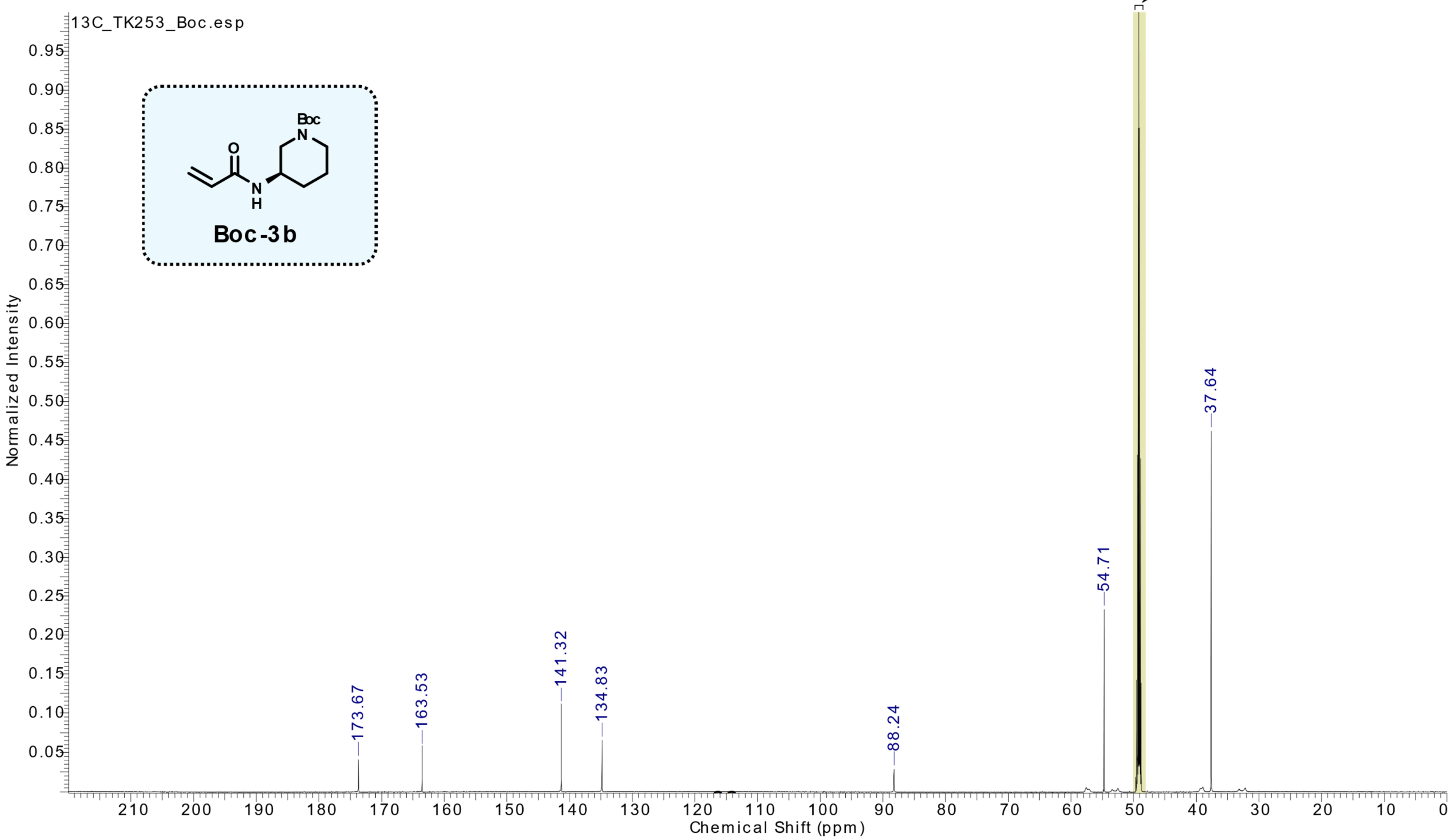

Figure S32: ^{1}H NMR Spectrum of (*S*)-*N*-(piperidin-3-yl)acrylamide 3c

^{1}H NMR (700 MHz) in CD_3OD

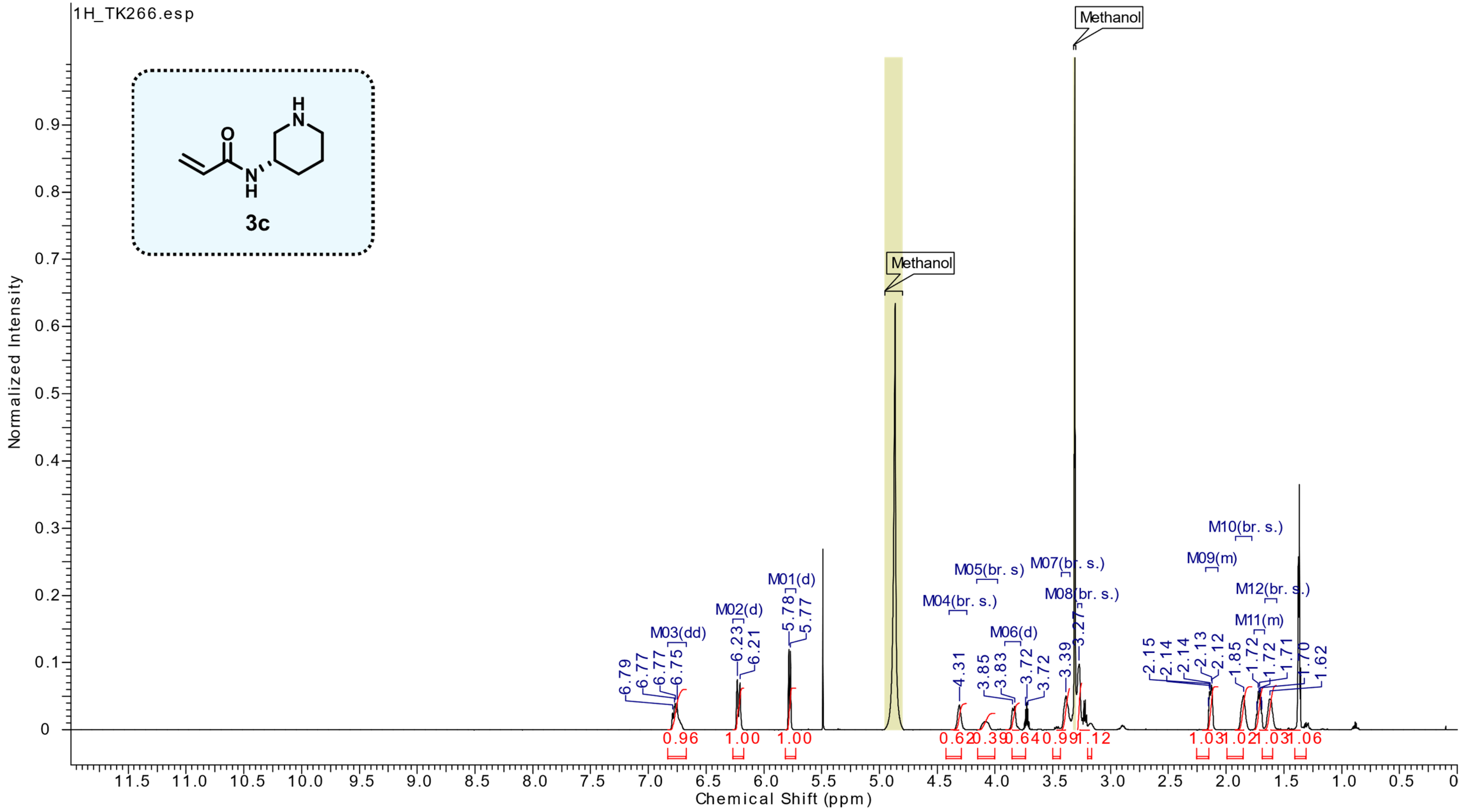

Figure S33: ^{13}C NMR Spectrum of (*S*)-*N*-(piperidin-3-yl)acrylamide 3c

^{13}C NMR (175 MHz) in CD_3OD

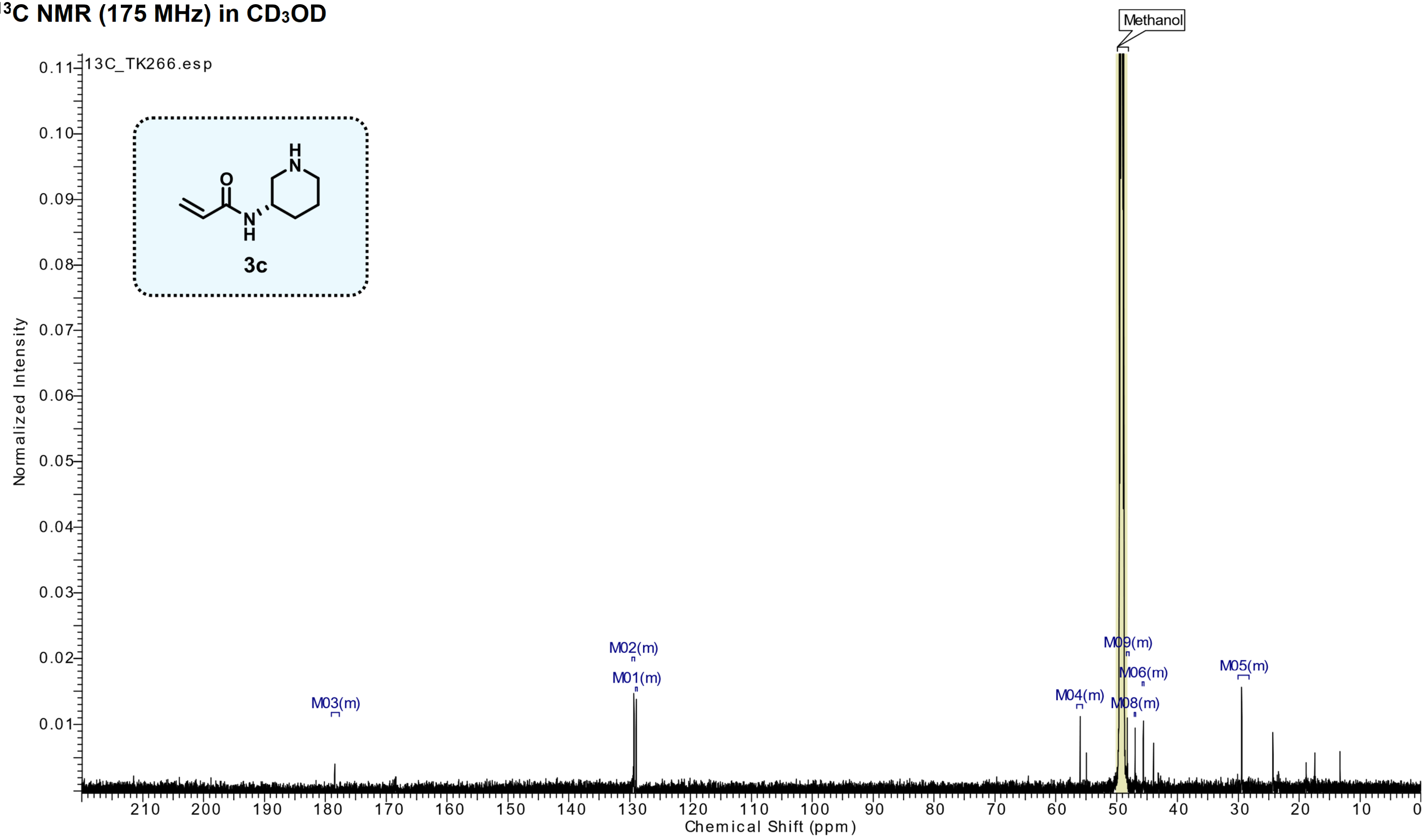

Figure S34: ^{1}H NMR Spectrum of (*R*)-*N*-(azepan-3-yl)acrylamide 3d

^{1}H NMR (600 MHz) in CD_3OD

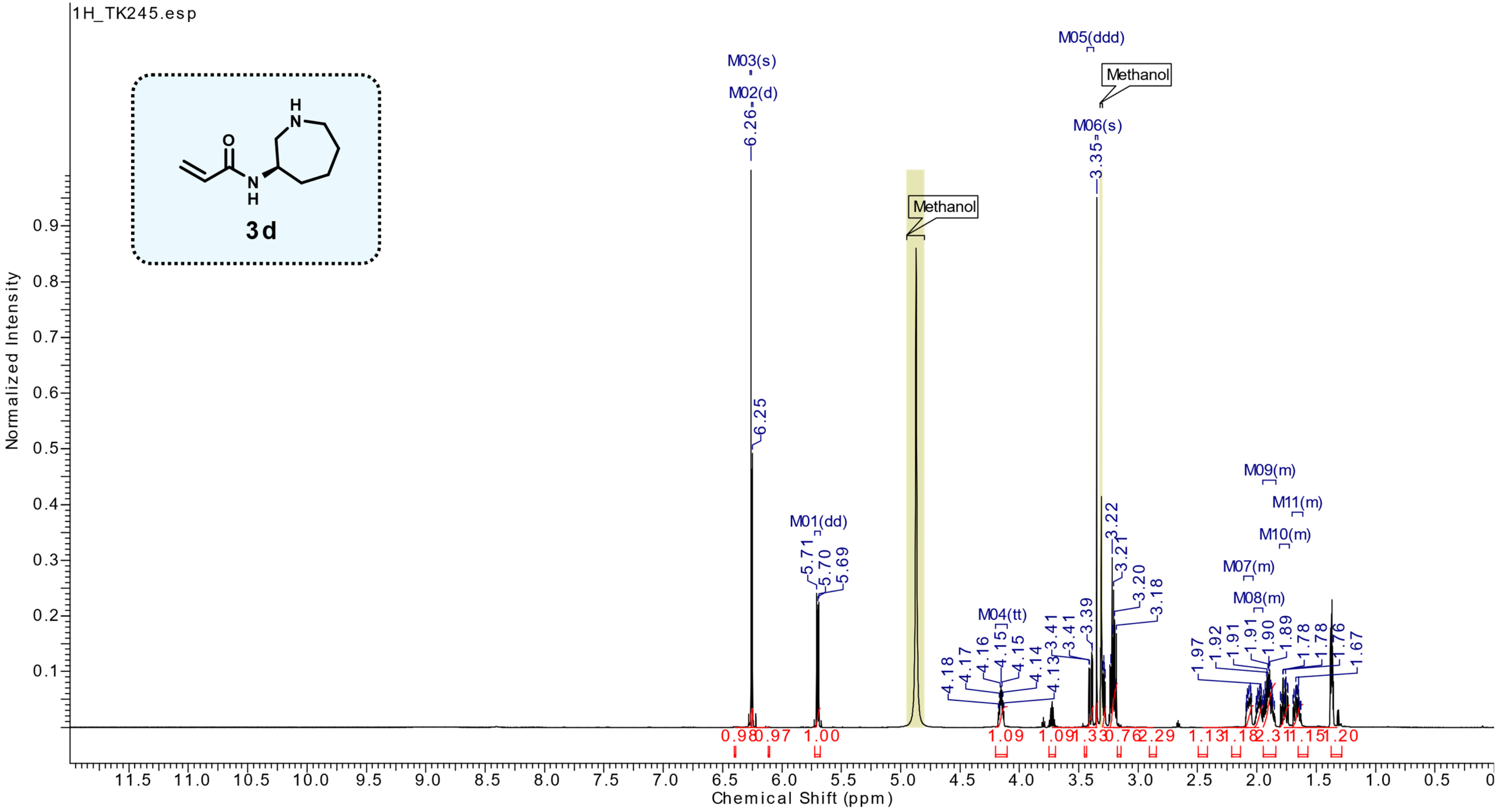

Figure S35: ^{13}C NMR Spectrum of (*R*)-*N*-(azepan-3-yl)acrylamide 3d

^{13}C NMR (150 MHz) in CD_3OD

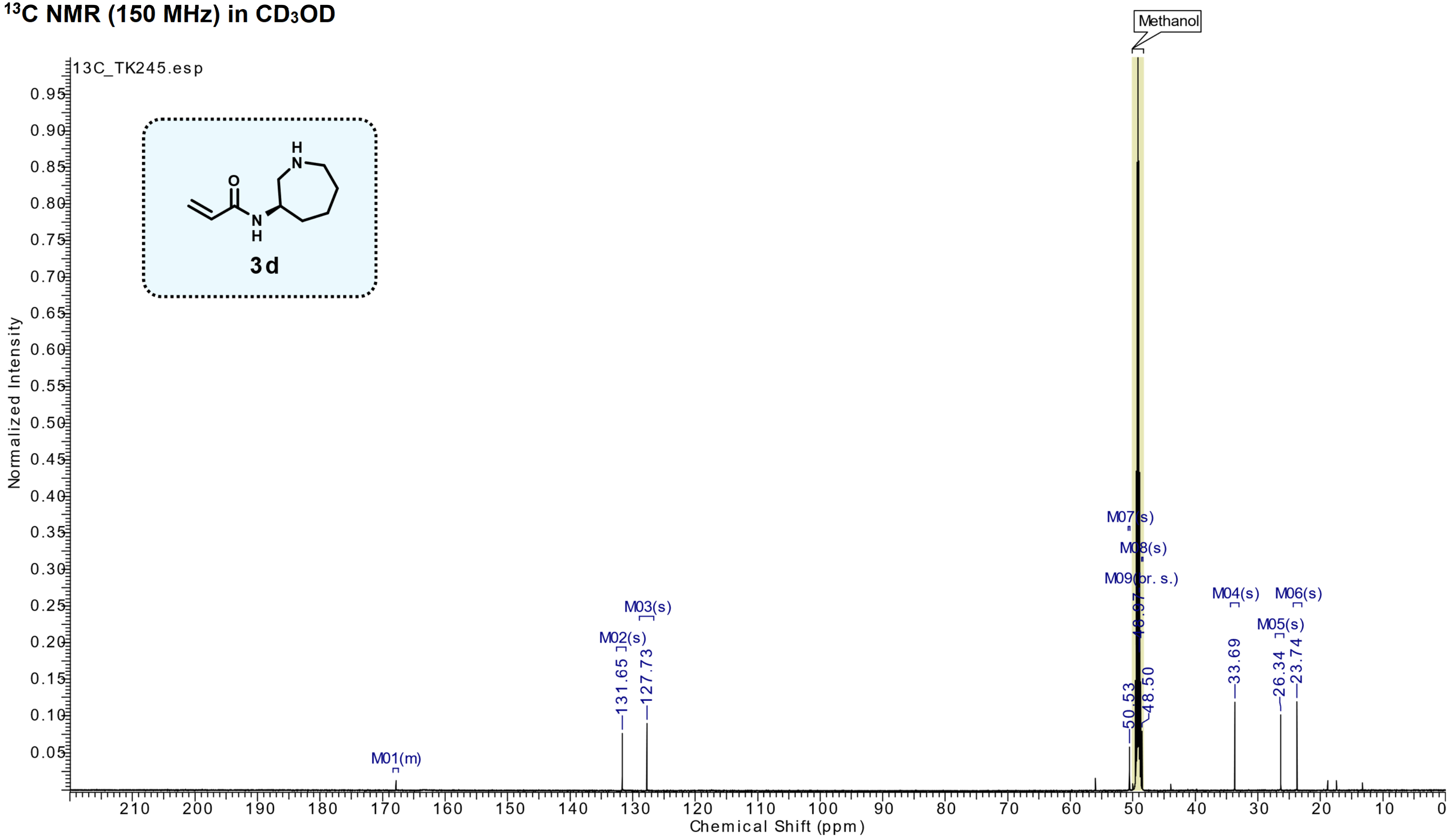

Figure S36: ^{1}H NMR Spectrum of *N*-(2-aminocyclohexyl)acrylamide 3e

^{1}H NMR (400 MHz) in CD_3OD

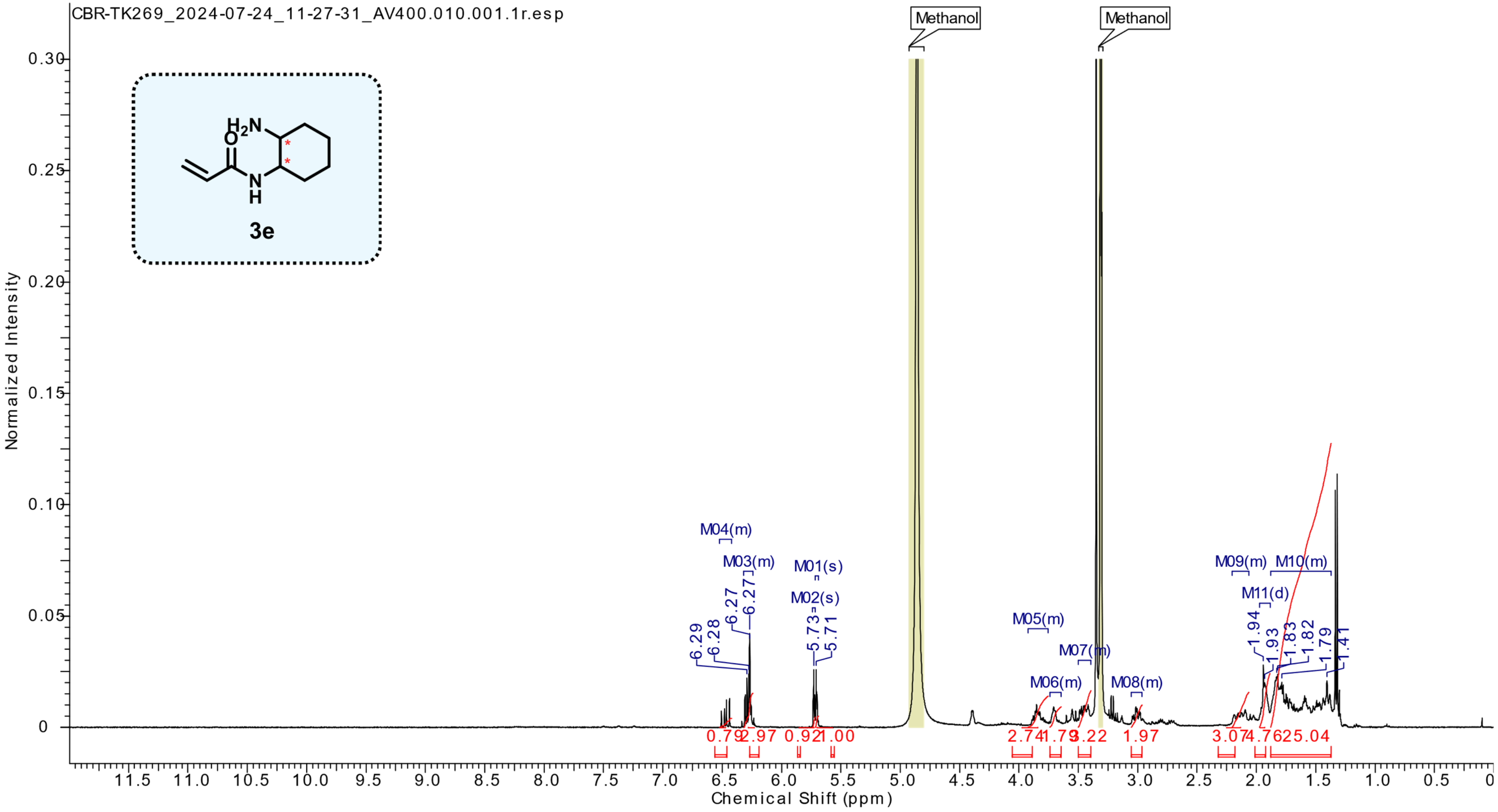

Figure S37: ^{13}C NMR Spectrum of *N*-(2-aminocyclohexyl)acrylamide 3e

^{13}C NMR (100 MHz) in CD_3OD

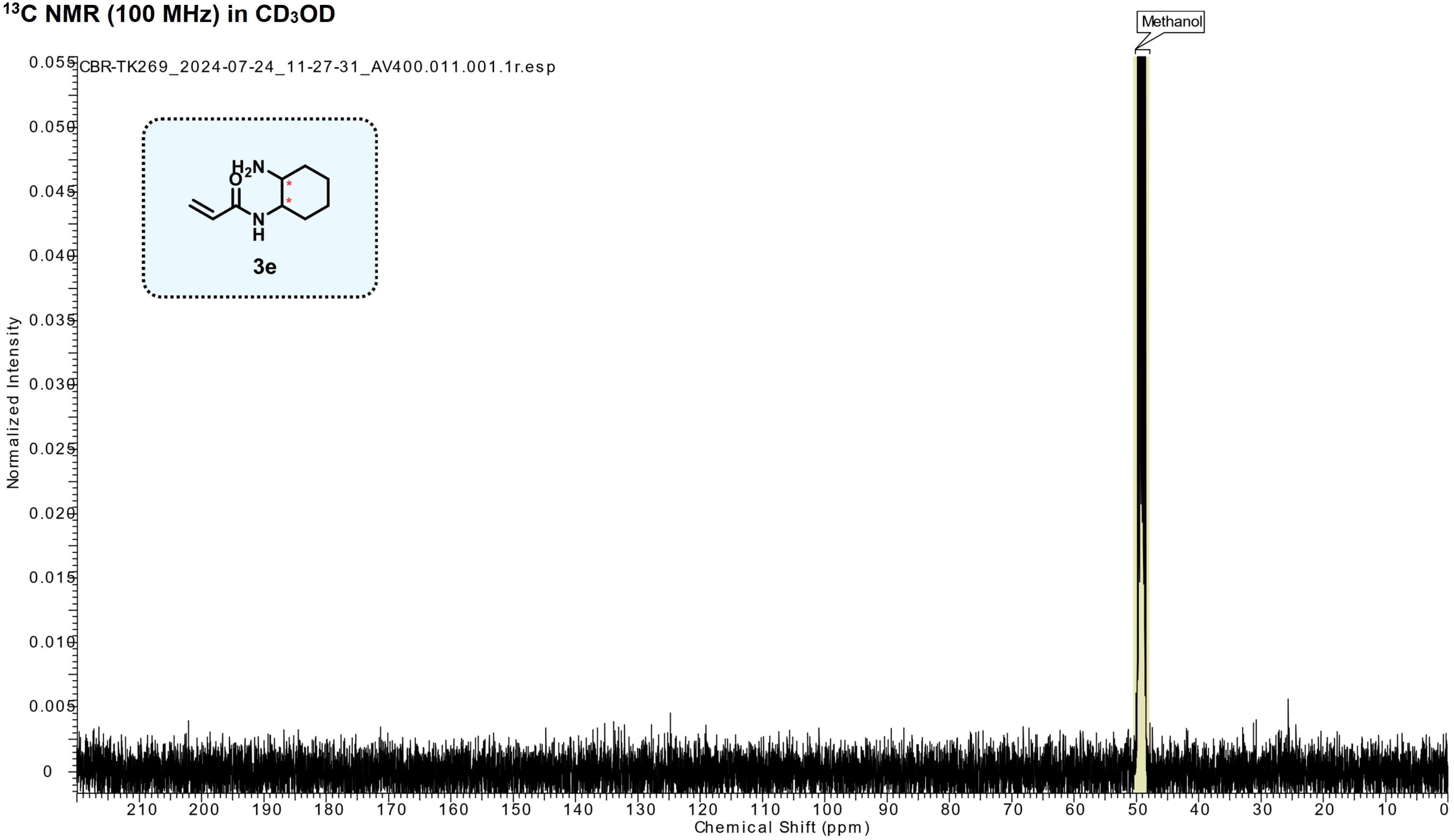

Figure S38: ^{1}H NMR Spectrum of (*R*)-*N*-(pyrrolidin-3-yl)acrylamide 3f

^{1}H NMR (600 MHz) in CD_3OD

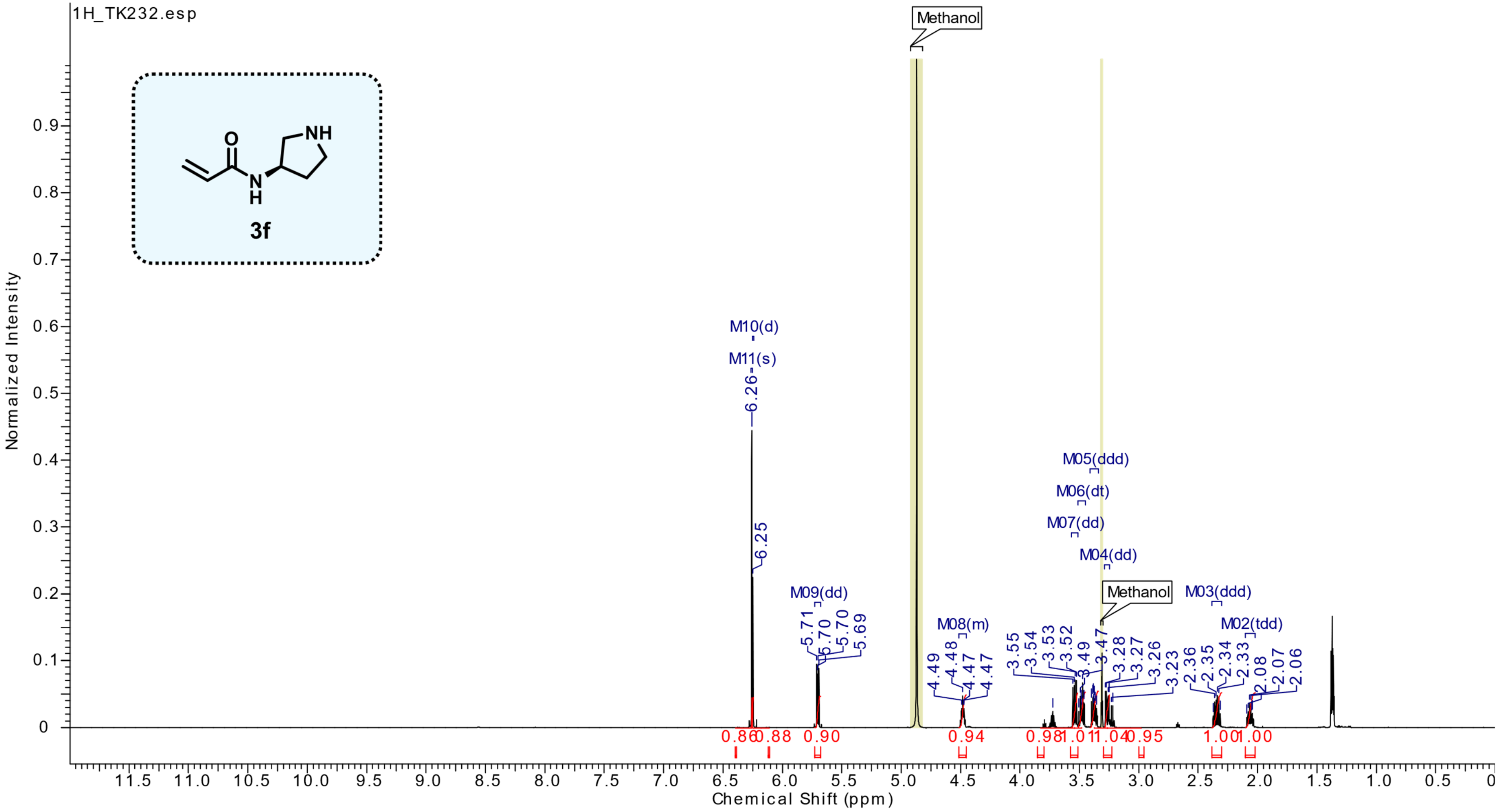

Figure S39: ^{13}C NMR Spectrum of (*R*)-*N*-(pyrrolidin-3-yl)acrylamide 3f

^{13}C NMR (150 MHz) in CD_3OD

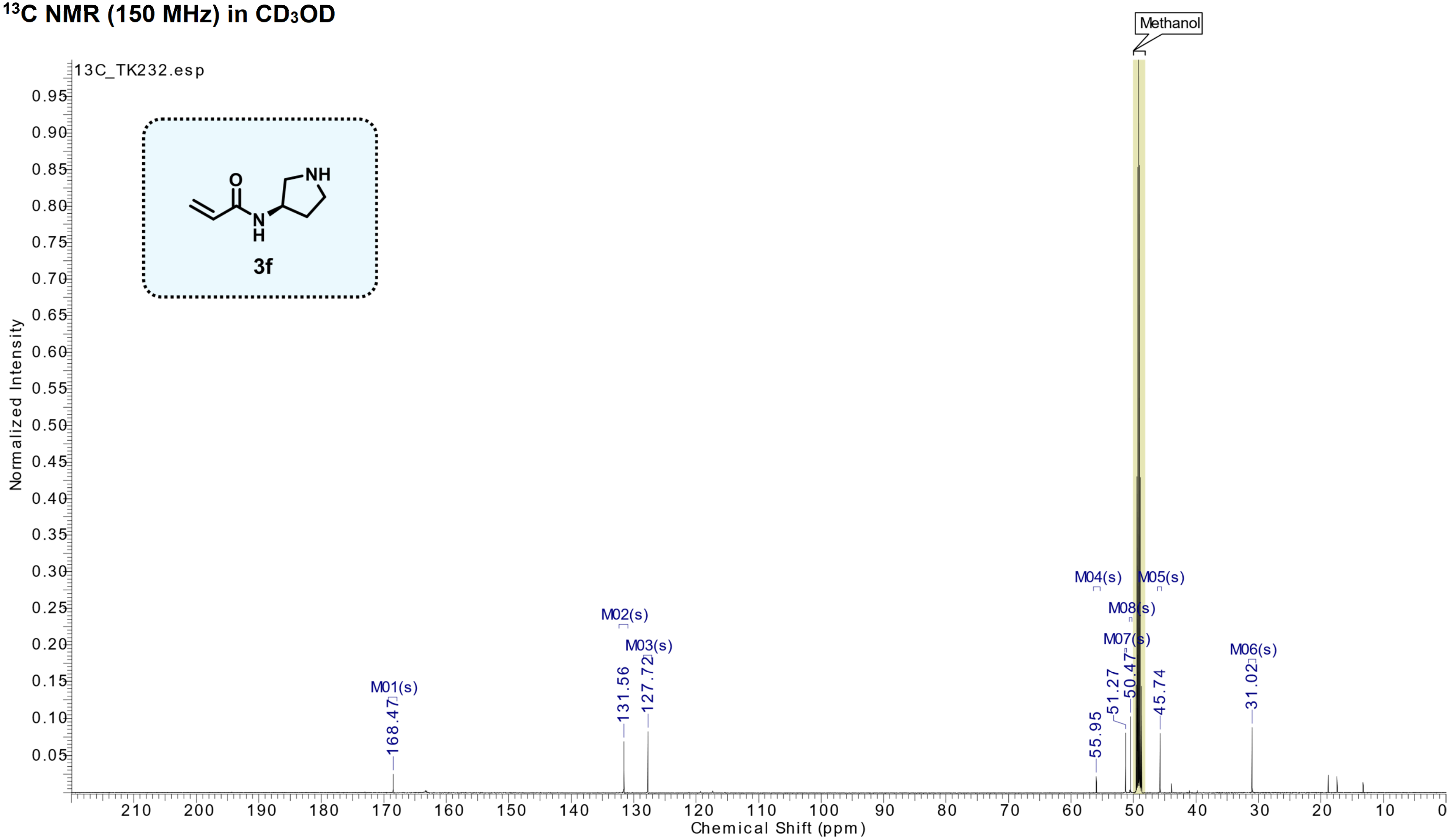

Figure S40: ^{1}H NMR Spectrum of (*S*)-*N*-(pyrrolidin-3-yl)acrylamide 3g

^{1}H NMR (600 MHz) in CD_3OD

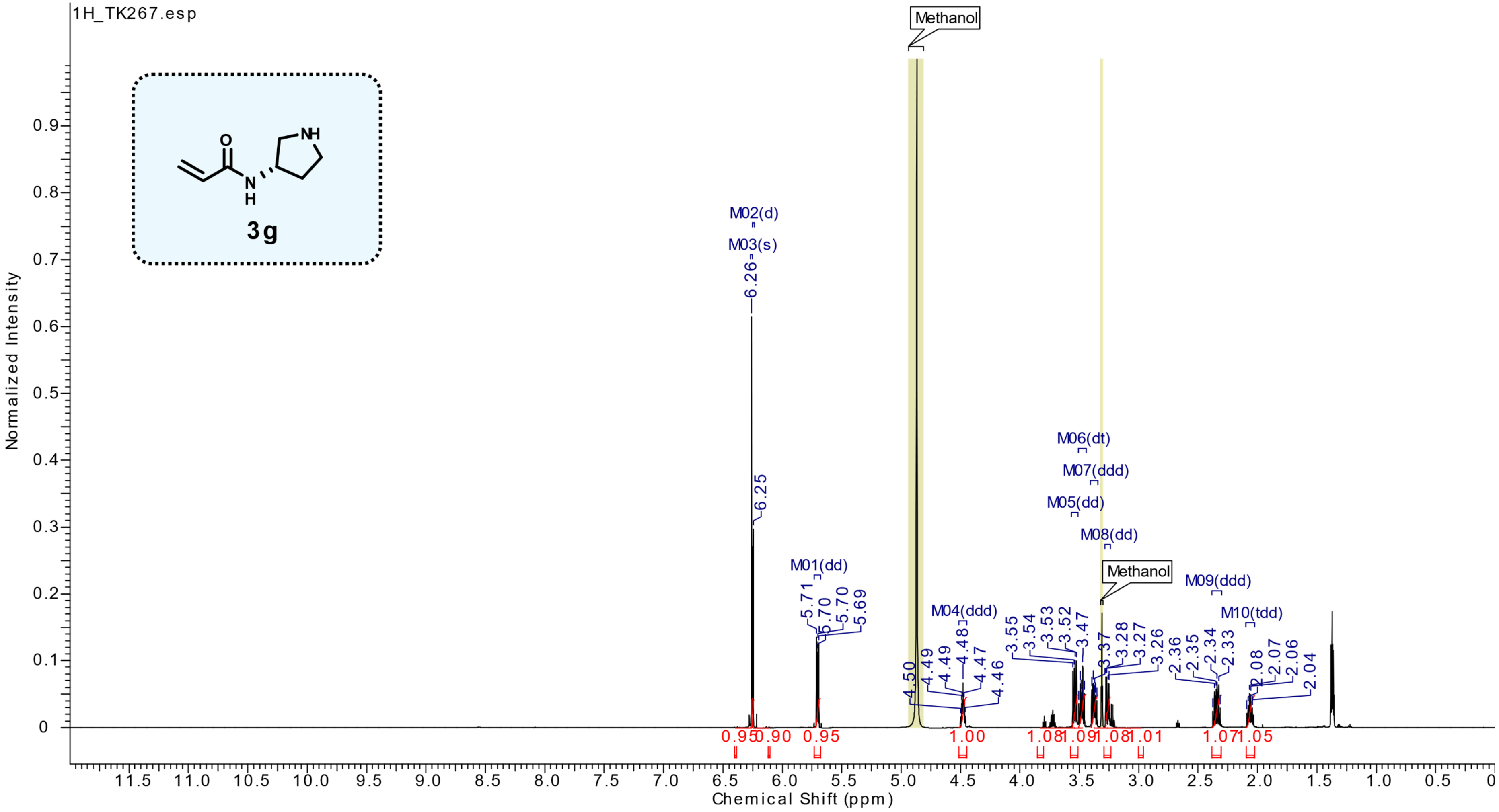

Figure S41: ^{13}C NMR Spectrum of (*S*)-*N*-(pyrrolidin-3-yl)acrylamide 3g

^{13}C NMR (150 MHz) in CD_3OD

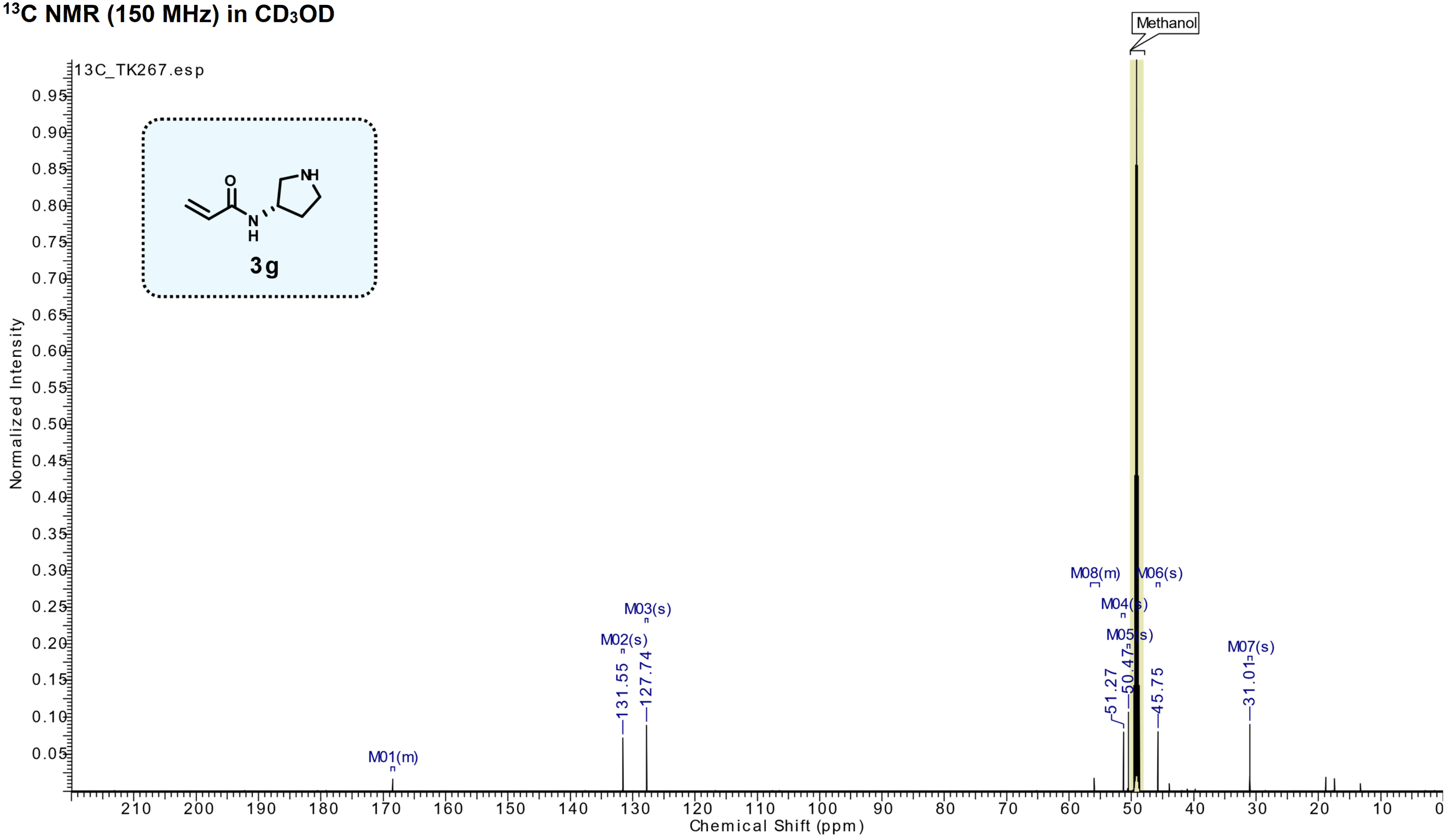

Figure S42: ¹H NMR Spectrum of *N*-(piperidin-4-yl)acrylamide hydrochloride 3h

¹H NMR (500 MHz) in DMSO-d6

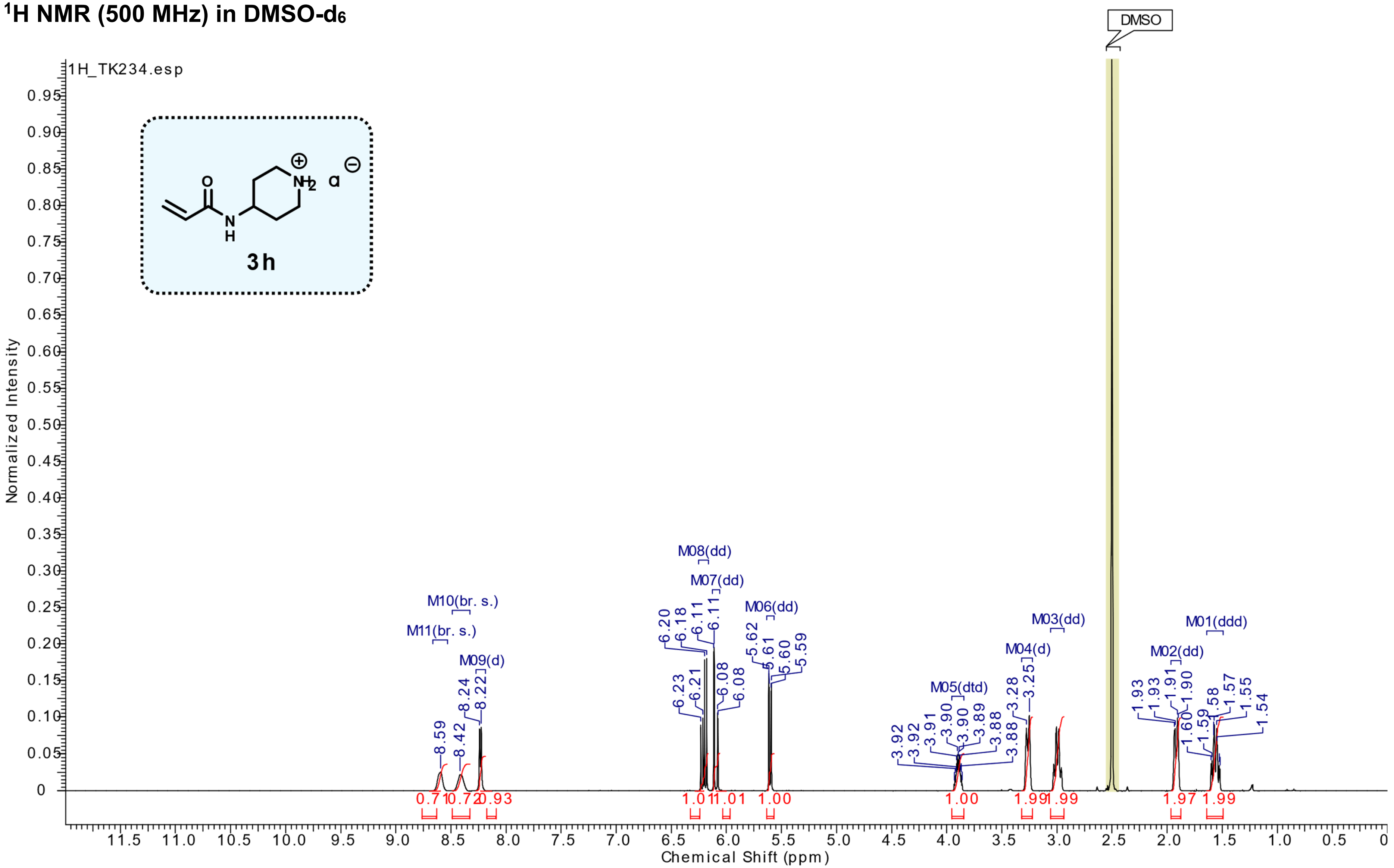

Figure S43: ^{13}C NMR Spectrum of *N*-(piperidin-4-yl)acrylamide hydrochloride 3h

^{13}C NMR (125 MHz) in DMSO-d$_6$

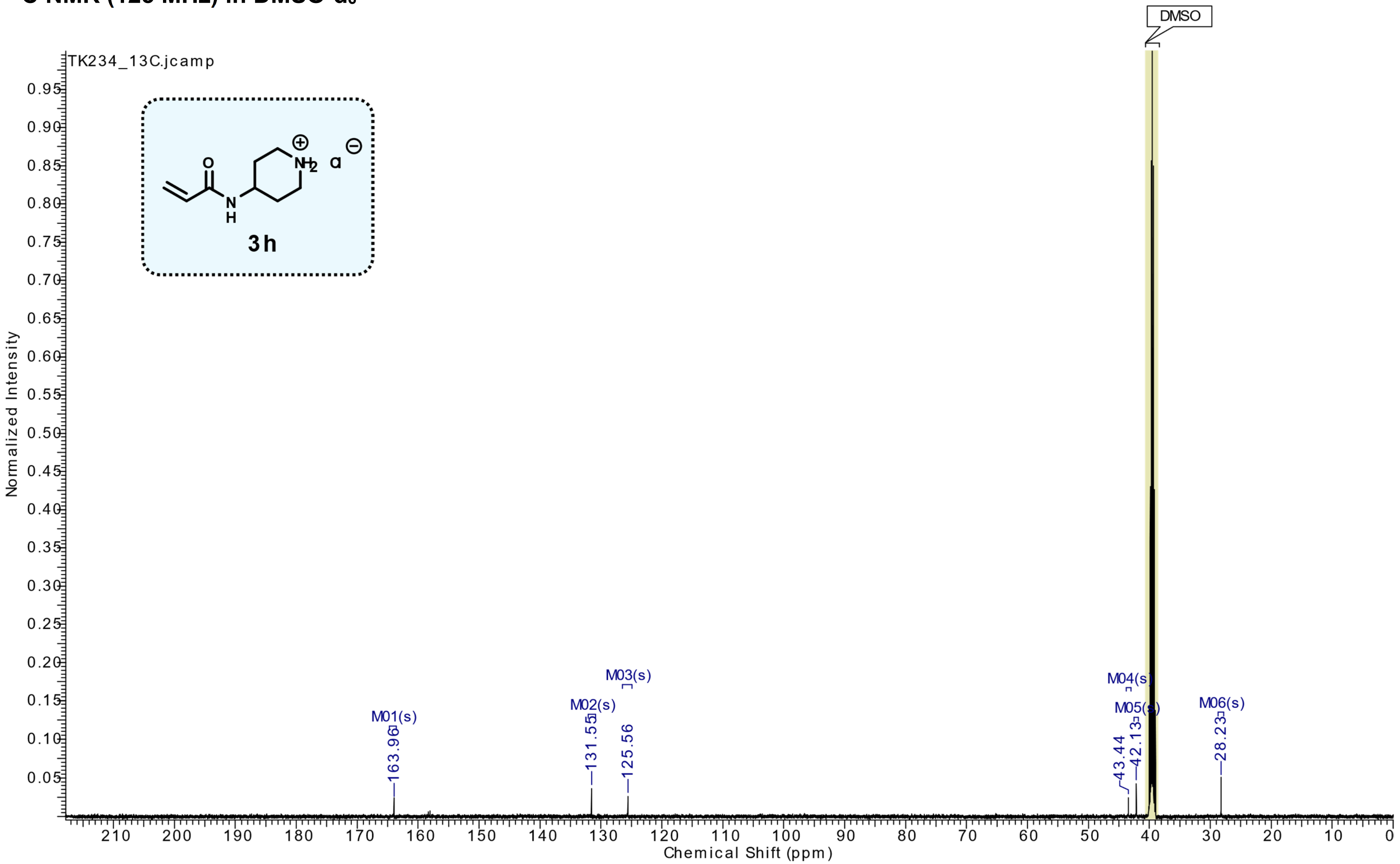

Figure S44: ^{1}H NMR Spectrum of 1-(4-aminopiperidin-1-yl)prop-2-en-1-one 3i

^{1}H NMR (600 MHz) in CD_3OD

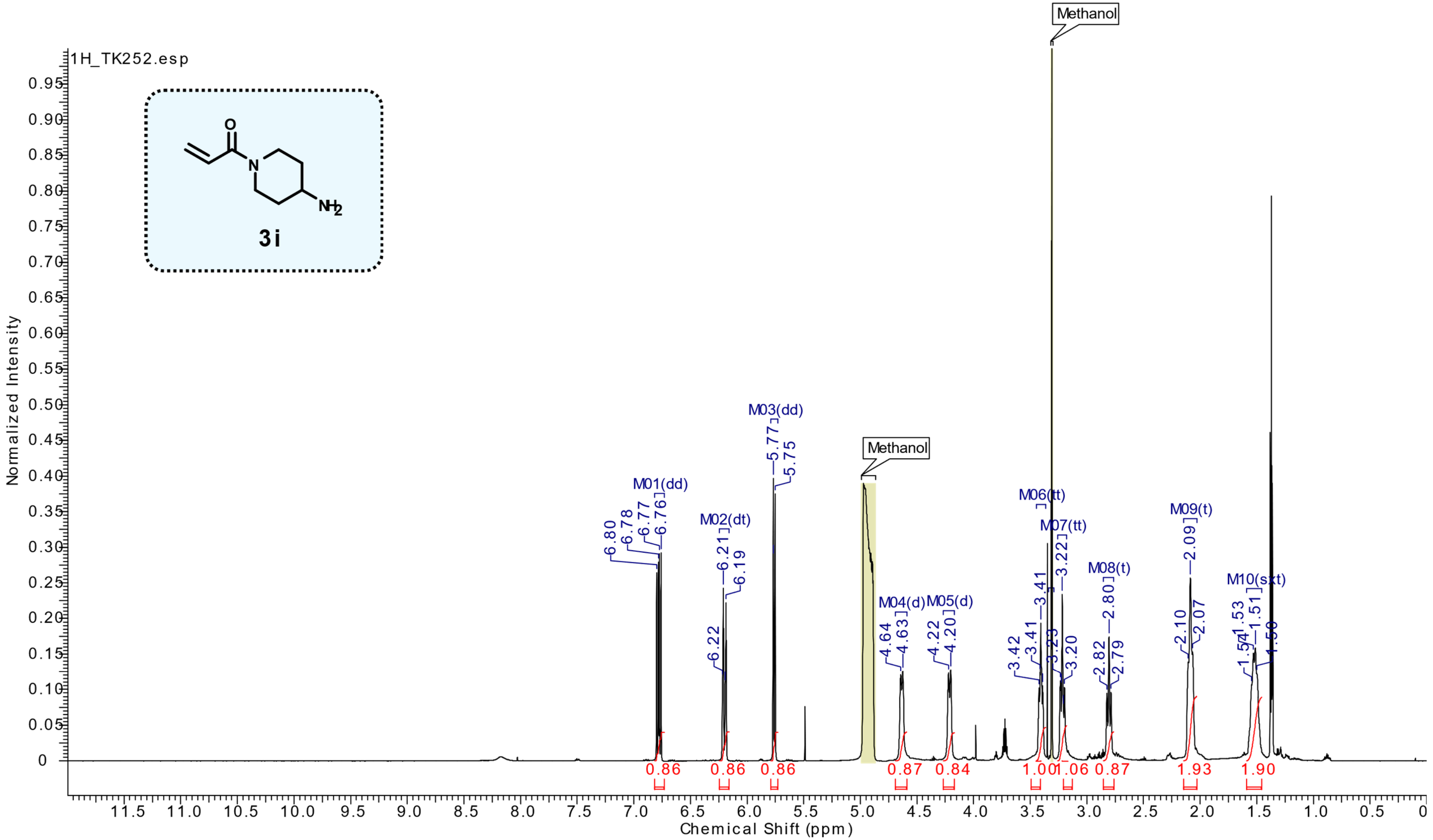

Figure S45: ^{13}C NMR Spectrum of 1-(4-aminopiperidin-1-yl)prop-2-en-1-one 3i

^{13}C NMR (150 MHz) in CD_3OD

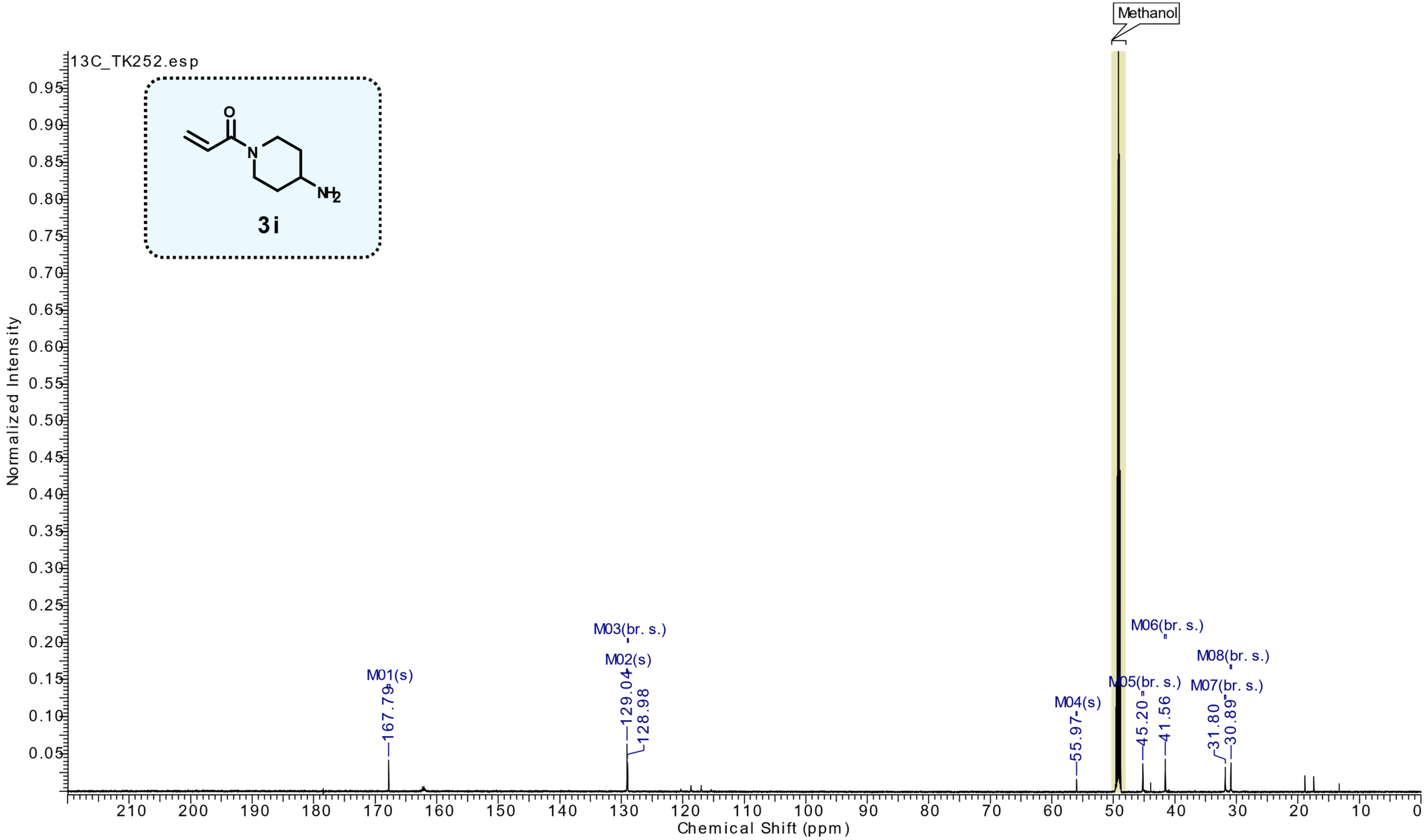

Figure S1. **Comparative analysis of modification of KRAS with the three compounds 7b–d** with the control compound eda-GDP (**7a**) was performed using the proteins $KRAS^{G13C}{}_{1–169}$ (Cys-light), $KRAS^{G12C}{}_{1–169}$ (Cys-light), and $KRAS^{wt}{}_{1–169}$ (from top to bottom). The covalent, percentage-based protein modification was plotted against the pH value.

Figure S2. Unprocessed SDS gel image for Figure 5, B.

Figure S3. A: Additional rotated view compared to Figure 6 showing that the linker is solvent-exposed. **B:** Comparison of KRAS:GDP (PDB id 4OBE; GDP is shown in green) and the 3 conformations of the linker modelled in this study. **C:** Comparison of compound 7a (PDB id 7OK3; compound 7a is shown in green) and the 3 conformations of the linker. Note the slight shift of the 2'- and 3'-OH groups likely induced by the strained linker (highlighted in red) and not observed in the panel above. **D-F:** Representation of the different modelled linker conformations of 7b within the three molecules in the asymmetric unit. The 2Fo-Fc and Fo-Fc electron density map is contoured at an r.m.s.d. of 1 and 2.8, respectively. PyMOL (version 3.1.0, W.L. DeLano, The PyMOL Molecular Graphics System) was used for generating the 3D figures.

Figure S4. Stability of GDP and Mg^{2+} during MD simulations of $KRAS^{G13C}$ non-covalently bound to compound **7a** (RMSD), built based on the 7OK3 crystal structure, demonstrating the accuracy of the CHARMM36 forcefield to represent the studied molecular systems, that is, the crystallographic geometry is well maintained during simulations. The same behavior was observed for the performed MD simulations for the remaining compounds.

Figure S5. **A**: Interaction between Mg^{2+} and nearby atoms involved in metal coordination. **B**: Radial distribution functions, g(r), for Mg^{2+} and oxygen atoms from water molecules up to 0.8 nm distance. Number of water molecules within 0.23 nm for each complex: **7a**: 2; **7b**: 2.5; **7c**: 2; **7d**: 2; **7f**: 2; **7i**: 2.8, compared to 3 water molecules on 4OBE crystal structure. PyMOL (version 3.1.0, W.L. DeLano, The PyMOL Molecular Graphics System) was used for generating the 3D figures.

Table S1. Distance between Mg^{2+} and the atoms of nearby residues involved in the coordination, for each ligand.

Figure S6. **A**-**F**: Conformational distribution of compounds **7a, b, c, d, f and i** observed in MD simulations. PyMOL (version 3.1.0, W.L. DeLano, The PyMOL Molecular Graphics System) was used for generating the 3D figures.

Table S2. Area under the curve for compounds **7a, b, c, d, f and i**, defined by a distance below to 0.5 nm Å between Cys13 and the oxygen atom from the α,β-unsaturated carbonyl group, as sampled during MD simulations.

Figure S7: Intermolecular Interaction Profile Between KRAS Protein and Ligands 7A, 7B, 7C, 7D, 7F and 7I. The figure illustrates the intermolecular interaction profile as a function of simulation time. The interactions are represented by the following colors: hydrophobic contact (green), van der Waals contact (yellow), hydrogen bond acceptor (light cyan), hydrogen bond donor (dark cyan), anionic interaction (blue), cation-π interaction (pink), and π-stacking interaction (purple). The residues marked in green highlight the P-loop region, those in orange indicate residues interacting with the phosphate group, residues in gray-green correspond to the Switch Loop 1, and those in blue represent residues interacting with GDP.

Figure S8: Comparison of two independent simulation replicas for compounds 7A-F. Each panel shows the fluctuation and the distribution of the distance between Cys13 sulfur atom and the oxygen atom from the α-β unsaturated carbonyl group. The graphics demonstrate that both replicas offer equivalent ensembles, demonstrating the statistical robustness and reproducibility of the performed simulations.

Figure S9: LC/MS Spectra of (R)-N-(piperidin-3-yl)acrylamide 3b

Figure S10: LC/MS Spectra of (S)-N-(piperidin-3-yl)acrylamide 3c

Figure S11: LC/MS Spectra of (R)-N-(azepan-3-yl)acrylamide 3d

Figure S12: LC/MS Spectra of N-(2-aminocyclohexyl)acrylamide 3e

Figure S13: LC/MS Spectra of (R)-N-(pyrrolidin-3-yl)acrylamide 3f

Figure S14: LC/MS Spectra of (S)-N-(pyrrolidin-3-yl)acrylamide 3g

Figure S15: LC/MS Spectra of N-(piperidin-4-yl)acrylamide hydrochloride 3h

Figure S16: LC/MS Spectra of 1-(4-aminopiperidin-1-yl)prop-2-en-1-one 3i

Figure S17: LC/MS Spectra of GDP 7a

Figure S18: LC/MS Spectra of GDP 7b

Figure S19: LC/MS Spectra of GDP 7c

Figure S20: LC/MS Spectra of GDP 7d

Figure S21: LC/MS Spectra of GDP 7e

Figure S22: LC/MS Spectra of GDP 7f

Figure S23: LC/MS Spectra of GDP 7g

Figure S24: LC/MS Spectra of GDP 7h

Figure S25: LC/MS Spectra of GDP 7i

Figure S26: HRMS Spectra of GDP 7b. $[M+H]^+$ with -0,9 ppm deviation; Overlap with another mass.

Figure S27: HRMS Spectra of GDP 7c. $[M+H]^+$ with -0,6 ppm deviation.

Figure S28: HRMS Spectra of GDP 7d. $[M+H]^+$ with -0,2 ppm deviation; Overlayed with another mass.

Figure S29: HRMS Spectra of GDP 7e. $[M+H]^+$ with 1,9 ppm deviation.

Figure S30: ^{1}H NMR Spectrum of (*R*)-*N*-(piperidin-3-yl)acrylamide 3b

Figure S31: ^{13}C NMR Spectrum of (*R*)-*N*-(piperidin-3-yl)acrylamide 3b

Figure S32: ^{1}H NMR Spectrum of (*S*)-*N*-(piperidin-3-yl)acrylamide 3c

Figure S33: ^{13}C NMR Spectrum of (*S*)-*N*-(piperidin-3-yl)acrylamide 3c

Figure S34: ^{1}H NMR Spectrum of (*R*)-*N*-(azepan-3-yl)acrylamide 3d

Figure S35: ^{13}C NMR Spectrum of (*R*)-*N*-(azepan-3-yl)acrylamide 3d

Figure S36: ^{1}H NMR Spectrum of *N*-(2-aminocyclohexyl)acrylamide 3e

Figure S37: ^{13}C NMR Spectrum of *N*-(2-aminocyclohexyl)acrylamide 3e

Figure S38: ^{1}H NMR Spectrum of (*R*)-*N*-(pyrrolidin-3-yl)acrylamide 3f

Figure S39: ^{13}C NMR Spectrum of (*R*)-*N*-(pyrrolidin-3-yl)acrylamide 3f

Figure S40: ^{1}H NMR Spectrum of (*S*)-*N*-(pyrrolidin-3-yl)acrylamide 3g

Figure S41: ^{13}C NMR Spectrum of (*S*)-*N*-(pyrrolidin-3-yl)acrylamide 3g

Figure S42: ^{1}H NMR Spectrum of *N*-(piperidin-4-yl)acrylamide hydrochloride 3h

Figure S43: ^{13}C NMR Spectrum of *N*-(piperidin-4-yl)acrylamide hydrochloride 3h

Figure S44: ^{1}H NMR Spectrum of 1-(4-aminopiperidin-1-yl)prop-2-en-1-one 3i

Figure S45: ^{13}C NMR Spectrum of 1-(4-aminopiperidin-1-yl)prop-2-en-1-one 3i
